# Analogues of Muraymycin Nucleoside Antibiotics with Epimeric Uridine-Derived Core Structures

**DOI:** 10.3390/molecules23112868

**Published:** 2018-11-03

**Authors:** Anatol P. Spork, Stefan Koppermann, Stephanie Schier (née Wohnig), Ruth Linder, Christian Ducho

**Affiliations:** 1 Department of Chemistry, Institute of Organic and Biomolecular Chemistry, Georg-August-University Göttingen, Tammannstr. 2, 37077 Göttingen, Germany; anatolspork@gmx.de (A.P.S.); swohnig@web.de (S.S.W.); ruth.linder@uni-saarland.de (R.L.); 2 Department of Pharmacy, Pharmaceutical and Medicinal Chemistry, Saarland University, Campus C2 3, 66123 Saarbrücken, Germany; stefan.koppermann@uni-saarland.de

**Keywords:** antibiotics, natural products, nucleoside analogues, structure–activity relationships.

## Abstract

Nucleoside analogues have found widespread application as antiviral and antitumor agents, but not yet as antibacterials. Naturally occurring uridine-derived ‘nucleoside antibiotics’ target the bacterial membrane protein MraY, an enzyme involved in peptidoglycan biosynthesis and a promising target for the development of novel antibacterial agents. Muraymycins represent a nucleoside-peptide subgroup of such MraY-inhibiting natural products. As part of detailed structure-activity relationship (SAR) studies on muraymycins and their analogues, we now report novel insights into the effects of stereochemical variations in the nucleoside core structure. Using a simplified version of the muraymycin scaffold, it was shown that some formal inversions of stereochemistry led to about one order of magnitude loss in inhibitory potency towards the target enzyme MraY. In contrast, epimers of the core motif with retained inhibitory activity were also identified. These 5′,6′-*anti*-configured analogues might serve as novel chemically tractable variations of the muraymycin scaffold for the future development of uridine-derived drug candidates.

## 1. Introduction

**Scheme 1:**
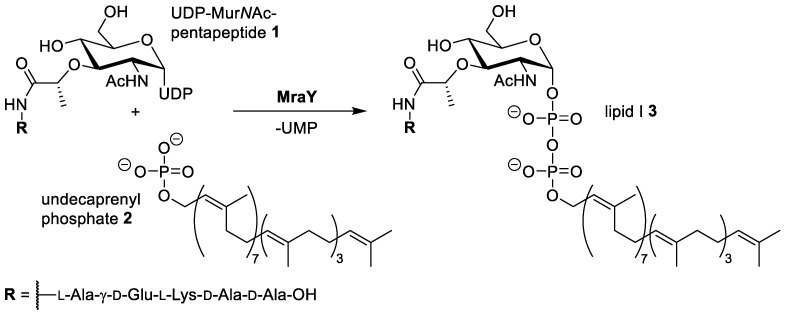
Reaction of Park’s nucleotide **1** with undecaprenyl phosphate **2** yielding lipid I **3** catalysed by translocase I (MraY). UDP = uridine diphosphate, UMP = uridine monophosphate. The exact composition of the pentapeptide moiety (residue **R**) can vary among different bacteria [11].

Infections with bacterial strains that have developed resistance against clinically used antibiotics are on the rise and represent a major challenge in healthcare [1,2]. New targets and new modes of action are needed to fight back such infections. Such novel targets can also be found in bacterial pathways that have already been addressed by many antibiotics [3]. MraY (translocase I) represents an example of a yet unexploited target, as it is part of bacterial peptidoglycan biosynthesis, i.e., one of the main pathways addressed by established antibiotics [4,5]. MraY catalyses the first membrane-associated step of peptidoglycan formation: the reaction of UDP-Mur*N*Ac pentapeptide (‘Park’s nucleotide’) **1** with the isoprenoid membrane anchor undecaprenyl phosphate **2**, thus yielding membrane-bound lipid I **3** (Scheme 1) [6,7,8,9,10,11,12].

**Figure 1:**
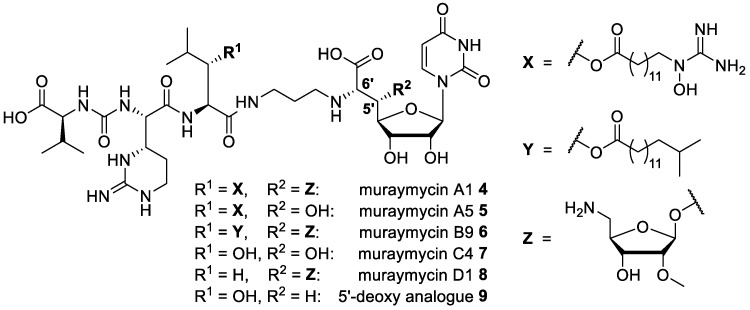
Structures of selected naturally occurring muraymycins **4**–**8** [21,23] and the previously reported synthetic 5′-deoxy analogue **9** [24].

MraY is a membrane protein with ten transmembrane helices and five cytosolic loops [13,14]. Methods for overexpression and isolation of the protein are available [15,16,17]. Several classes of natural products that inhibit MraY have been found over the years (‘nucleoside antibiotics’, e.g., muraymycins, caprazamycins, liposidomycins, capuramycins, and mureidomycins) [12,18,19,20]. We are mainly interested in muraymycins and their synthetic analogues, as they represent promising lead structures for antibacterial drug development. Muraymycins were originally discovered as a group of 19 structurally related secondary metabolites from *Streptomyces* [21,22]. Very recently, new and more active members of the muraymycins were isolated from the same strains [23]. The principle structure of muraymycins consists of a (5′*S*,6′*S*)-glycyluridine (GlyU) motif (representing a ′high-carbon′ nucleoside) and a urea peptide moiety containing the arginine-derived non-proteinogenic amino acid epicapreomycidine, with both units connected by an alkyl linker (Figure 1). Many naturally occurring muraymycins carry an *O*-aminoribosyl residue at the nucleosidic 5′-position. Muraymycins have been assigned to four subclasses with respect to the central l-leucine motif: classes A–C feature a (3*S*)-3-hydroxyl-l-leucine that is further functionalised with fatty acyl motifs in the A- and B-series. The A-series has ω-functionalised fatty acyl units bearing a terminal (*N*-hydroxy-)guanidine group (e.g., A1 **4** and A5 **5**), whereas the B-series has branched alkyl chains instead (e.g., B9 **6**). The C-series (e.g., C4 **7**) is not *O*-acylated in the 3-hydroxyl-l-leucine moiety, and the D-series (e.g., D1 **8**) is characterised by a nonhydroxylated l-leucine residue.

In 2016, Chung et al. reported an X-ray crystal structure of MraY from the extremophile *Aquifex aeolicus* in complex with muraymycin D2 (which differs from D1 **8** by the lack of the methyl ether at the amino ribose (**Z**), Figure 1) [25]. By comparison of this co-crystal structure with the previously described ligand-free *apo* enzyme structure [14], a pronounced conformational plasticity of the enzyme was revealed, with some amino acids moving up to 17 Å upon inhibitor binding [25,26]. In combination with the complex structures of the inhibitors, this makes computer-aided drug design approaches challenging and less reliable for the prediction of binding modes and structure–activity relationship (SAR) for other muraymycins and their analogues. Thus, the synthesis and biological testing of novel analogues represents a crucial approach in obtaining additional and more detailed insights into the interactions of muraymycin-type inhibitors with their bacterial target MraY.

The total synthesis of naturally occurring muraymycins is challenging with respect to several unusual structural motifs in the muraymycin scaffold (in particular, the 5′-*O*-aminoribosylated ′high-carbon′ nucleoside core, the epicapreomycidine, and the (3*S*)-3-hydroxyl-l-leucine units). Important contributions to the total synthesis of muraymycins and/or the aforementioned structural motifs were made by Ichikawa and Matsuda, Kurosu, and our group [12,24,27,28,29,30,31,32,33,34]. Several SAR studies on muraymycin analogues (including structurally simplified congeners) have already been conducted [12,24,35,36,37,38,39]. For instance, Ichikawa, Matsuda, and coworkers showed that the epicapreomycidine unit can be substituted with simpler amino acids without being deprived of antibacterial activity. This finding was solely based on antibacterial minimum inhibitory concentrations (MIC values), i.e., no in vitro data on MraY inhibition were reported for such analogues [38]. We have recently re-investigated selected naturally occurring muraymycins for their properties as MraY inhibitors. By that, we showed that the lack of the fatty acid moiety (such as in **7** or **8**) eliminates antimicrobial activity (likely due to hampered cellular uptake) but hardly affects the target interaction [40]. Regarding structural simplifications of the muraymycin scaffold, we have reported 5′-defunctionalised (‘5′-deoxy’) analogues of the uridine-derived muraymycin core unit, i.e., congeners not only lacking the aminoribosyl motif, but any substituent in the 5′-position [24,41,42]. For the 5′-deoxy analogue **9** of muraymycin C4 **7** (Figure 1), it was demonstrated that a corresponding full-length muraymycin derivative of this type can still be a fairly potent inhibitor of MraY (IC_50_ = 95 ± 19 nM for **9**) [40]. Thus, the 5′-deoxy variation might be useful to derive novel chemically more tractable lead structures for antibacterial drug development. It might also serve as a simplified scaffold for further SAR studies, for instance, on the muraymycin peptide unit.

However, it has been unknown so far if further variations of the nucleoside core structure might be feasible without hampering inhibition of the target enzyme MraY. In particular, stereochemical variations have only scarcely been studied. It was therefore the goal of this work to investigate simplified muraymycin analogues with epimeric configurations in the ′high-carbon′ uridine-derived nucleoside core. During our studies on 5′-deoxy analogue **9**, we have made the unexpected observation that 5′-hydroxylated synthetic intermediates (i.e., protected uridine-derived building blocks with the native (5′*S*,6′*S*)-*syn*-configuration) were fairly unstable and therefore prone to decomposition [24]. Based on the assumption that the intrinsic instability of these intermediates originates from their 5′,6′-*syn-*configuration, we turned to the respective 5′,6′-*anti*-configured congeners. Thus, another objective was to investigate if epimeric *anti*-configured variations ((5′*R*,6′*S*) and (5′*S*,6′*R*), respectively) might be sufficiently stable for the synthesis of full-length muraymycins and if the resultant analogues would be MraY inhibitors. Finally, we also aimed to study the interplay of stereochemical variations in the nucleoside core and an epimeric configuration in the peptide unit. We therefore decided to include d-leucine derivatives (in contrast to the native l-configuration in this position) in our studies.

**Scheme 2:**
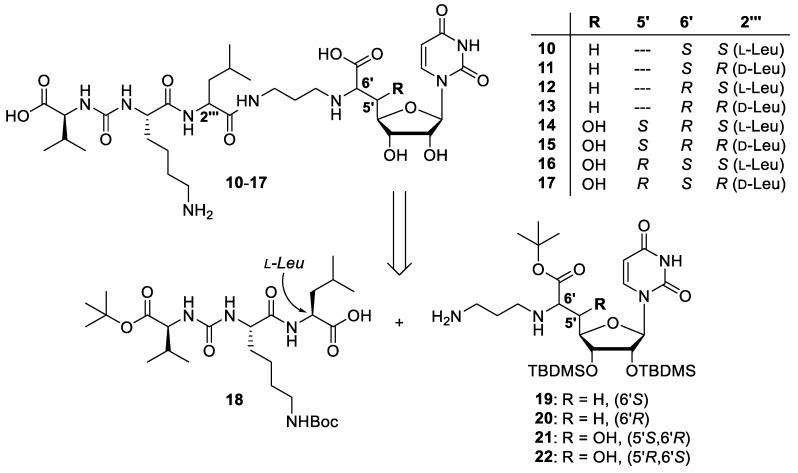
Target structures **10**–**17** of this study and retrosynthetic strategy for their preparation.

The aforementioned considerations led to the design of target structures **10**–**17** (Scheme 2). In order to limit synthetic effort, the scaffold of the previously reported 5′-deoxy analogue **9** was further simplified (l-lysine instead of the cyclic arginine derivative epicapreomycidine, nonhydroxylated l-leucine (as in muraymycin D1 **8**) in the central section), thus furnishing analogue **10** with the native (6′*S*)-configuration in the nucleoside core. Compound **11** represents the leucine (i.e., 2′′′) epimer of **10**, and target structures **12** and **13** are the 6′-epimers of **10** and **11**, respectively. Compounds **14**-**17** are *anti*-configured 5′-hydroxylated analogues with one epimeric configuration (relative to the 5′,6′-*syn*-configured natural products) in either the 5′- or the 6′-position. In the case of **15** and **17**, this variation was combined with the non-natural d-leucine (i.e., (2′′′*R*)) motif. It was our goal to prepare this series of muraymycin analogues in an efficient manner and to subject them to an established fluorescence-based in vitro assay for MraY inhibition [15,40,43]. Thus, we wanted to identify stereochemical variations of the muraymycin scaffold with potentially retained inhibitory activities towards the bacterial target enzyme MraY.

## 2. Results

### 2.1. Synthesis of Muraymycin Epimers

For our previous synthesis of 5′-deoxy muraymycin analogue **9**, we had reported a tripartite approach using protected building blocks for the uridine-derived nucleoside core, the terminal urea dipeptide motif and the central section [24]. However, for the sake of efficiency, we have decided to pursue a bipartite strategy in this work as most of the structural variations would be in the nucleoside core. Hence, such a bipartite approach was envisioned to be more convergent. The dissection of target structures **10**–**17** into two principle building blocks led to the protected urea tripeptide **18** and aminoalkylated nucleosyl amino acids **19**–**22** (Scheme 2). Peptide coupling and subsequent global acidic deprotection of the two building blocks was envisaged to afford the target compounds. It was initially unclear if the peptide coupling of **18** with either uridine derivative **19**–**22** proceeds without epimerisation. Hence, it was decided to prepare the l-leucine-containing tripeptide **18** first in order to initially elucidate the stereochemical outcome of the key coupling reaction before preparing the d-leucine-containing derivative of **18**.

**Scheme 3:**
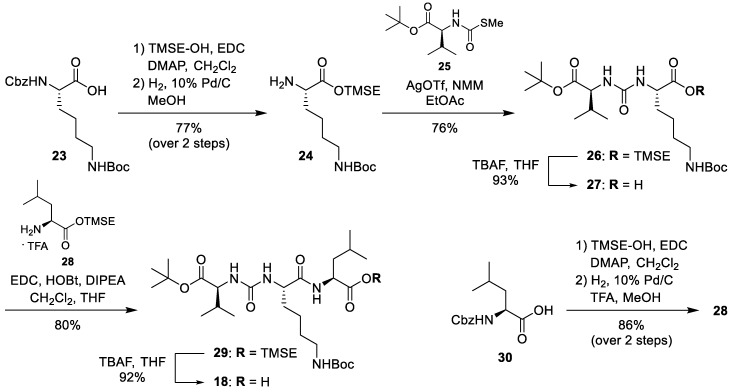
Synthesis of the urea tripeptide building block **18**. TMSE = 2-(trimethylsilyl)ethyl.

Protected urea tripeptide **18** was synthesised from corresponding amino acid building blocks (Scheme 3). Commercially available *N*^α^-Cbz-*N*^ε^-Boc-protected l-lysine **23** was converted into its 2-(trimethylsilyl)ethyl (TMSE) ester using a sequence of 1-ethyl-3-(3-dimethylaminopropyl)carbodiimide hydrochloride (EDC)-mediated esterification and *N*^α^-Cbz deprotection (77% yield over two steps). The resultant product **24** was coupled with *N*-(*S*-methyl thiocarbonyl)-l-valine-*tert*-butyl ester **25** [28] in the presence of silver triflate to give **26** in 76% yield. TMSE deprotection with tetrabutylammonium fluoride (TBAF) then afforded the free carboxylic acid **27** in 93% yield. Urea dipeptide **27** was coupled with l-leucine TMSE ester **28** (TFA salt) to give urea tripeptide ester **29** in 80% yield. Subsequent cleavage of the TMSE ester furnished the urea tripeptide building block **18** in 92% yield. The l-leucine TMSE ester **28** had been obtained in a similar manner as its l-lysine congener from *N*-Cbz-l-leucine **30** via a sequence of EDC-mediated esterification and *N*-Cbz deprotection (86% yield over 2 steps, Scheme 3).

**Scheme 4:**
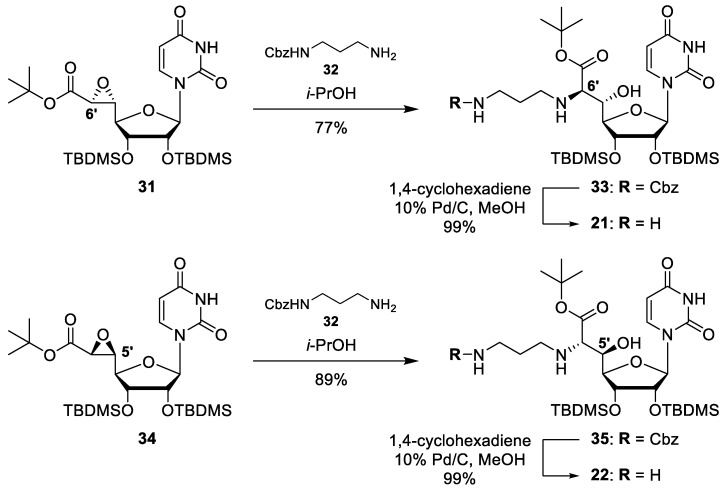
Synthesis of uridine-derived building blocks **21** and **22**.

The stereocontrolled synthesis of the aminoalkylated 5′-deoxy nucleosyl amino acids **19** and **20** has been reported before [41]. For the preparation of the 5′-hydroxylated building blocks **21** and **22**, we have employed our previously described stereocontrolled syntheses of corresponding epoxy ester precursors [44]. In the case of 6′-epimer **21**, epoxy ester **31** was obtained via stereoselective reaction of a sulphur ylide with a uridine-5′-aldehyde precursor (reactions not shown) [31,45,46,47]. Epoxide **31** was then reacted with *N*^1^-(Cbz)-1,3-diaminopropane **32** in a regioselective S_N_2-type transformation affording amino alcohol **33** in 77% yield (Scheme 4). Subsequent *N*-Cbz deprotection (99% yield) gave the 6′-*epi* building block **21**. For 5′-epimer **22**, epoxy ester **34** was obtained via Sharpless epoxidation (reactions not shown) [44,47]. Epoxide **34** was then subjected to a similar sequence of regioselective S_N_2-type ring opening (product **35**, 89% yield) and *N*-Cbz deprotection (99% yield) to afford the 5′-*epi* building block **22** (Scheme 4).

**Scheme 5:**
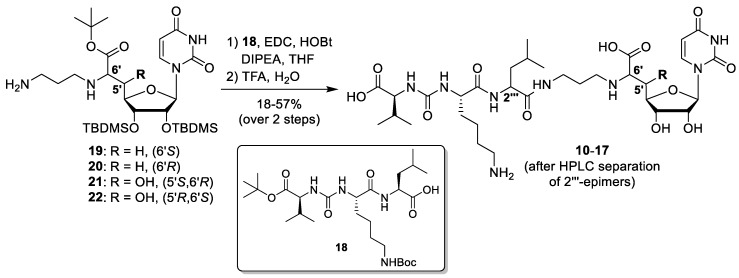
Synthesis of target structures **10**–**17**.

With all required building blocks in hand, the target structures could be prepared. The urea tripeptide building block **18** was activated with EDC in the presence of HOBt and then treated with amines **19**–**22** (Scheme 5). Corresponding coupling products were not characterised but directly employed in global acidic deprotections with 80% TFA in water. For each two-step transformation of this type, two compounds were isolated by preparative HPLC, showing identical masses and connectivities (as proven by NMR spectroscopy). It was concluded that the activated leucine unit in tripeptide **18** must have undergone epimerisation in the coupling step, which implied that the respective l-leucine and d-leucine epimers were isolated in pure form (after HPLC) from just one two-step transformation for every variation of the nucleoside core. This was unambiguously proven by acidic hydrolysis of the respective stereoisomerically pure peptidic products, subsequent derivatisation with Marfey’s reagent and HPLC analysis, which also furnished the stereochemical assignment of the leucine unit (see Supplementary Materials). Hence, it was not necessary to prepare the d-leucine epimer of **18** as all target structures (**10**–**17)** were synthesised from one urea tripeptide precursor in a highly efficient manner (Scheme 5).

### 2.2. Biological Evaluation

Target compounds **10**–**17** were tested in vitro for their inhibitory potency towards the bacterial target enzyme MraY. Therefore, a previously reported fluorescence-based assay was employed, using dansylated Park’s nucleotide and MraY from *S. aureus* (recombinantly overexpressed in *E. coli*) [15,23,40,43,48]. None of the tested muraymycin analogues showed any interfering autofluorescence at the employed wavelength (*λ*_ex_ = 355 nm). The thus obtained inhibitory activities (IC_50_ values) are listed in Table 1.

Muraymycin analogues **10**–**17** were also studied for their antibacterial activities against *E. coli*, but were not active (MIC > 50 μg/mL). This was in agreement with the results obtained for previously reported 5′-deoxy analogue **9** (which proved to be more potent as an MraY inhibitor) which was weakly active against bacterial growth [24].

## 3. Discussion

All newly synthesised muraymycin epimers **10**–**17** showed μm inhibitory activities in vitro against the bacterial target protein MraY. This is in accordance with previously reported observations that exchanging the epicapreomycidine moiety to simpler amino acids preserves inhibitor potency to a large extent, even though this finding was mainly based on antibacterial activities (MIC values) [38]. In our series of 5′-deoxy analogues, replacing epicapreomycidine for l-lysine and inserting nonhydroxylated l-leucine in the central position led to a ~26-fold loss in inhibitory potency (compounds **10** vs. **9**). However, this implied that **10** was still a reasonably potent (i.e., low μm) MraY inhibitor.

A comparison of MraY inhibitory activities of **10**–**17** (Table 1) revealed that the configuration of the leucine moiety had only a weak influence on inhibitory potencies, with the notable exception of **16** vs. **17**. In this case, the native l-configuration was preferred as **16** was ~3-fold more active than **17**. The reason for this is not fully clear, but it is possible that the non-native (5′*R*)-configuration in **16** and **17** might lead to a slightly different binding mode of these MraY inhibitors relative to **10**–**13** (5′-deoxy) as well as **14** and **15** (5′*S*). The influence of the configuration in the 6′-position was much more pronounced with the most distinct difference for the 5′-deoxy (6′*R*)-derivatives **12** and **13** (which were 15-fold and 12-fold weaker inhibitors than their (6′*S*)-counterparts **10** and **11**, respectively). This decrease in inhibitory potency of the (6′*R*)-configured analogues was largely compensated by the presence of a 5′-hydroxy group if the latter was oriented in the native (5′*S*)-configuration (analogues **14** and **15**). Interestingly, the (5′*R*,6′*S*)-analogue **16** also showed almost completely retained activity, suggesting that at least one of the two substituents at the 5′- and 6′-position needs to be in its natural orientation (in the 5′-hydroxylated series of compounds).

In conclusion, these results indicate that the effect of formal stereochemical inversions in the muraymycin nucleoside core (relative to the native scaffold) on MraY inhibition can be limited if a suitable structural motif is chosen. This is particularly the case for 5′,6′-*anti*-configured 5′-hydroxy analogues (except for d-leucine congener **17**). This series of compounds turned out to be stable and chemically tractable, in contrast to the previously reported native (5′*S*,6′*S*)-*syn*-configured motif and its intrinsic lability [24]. The synthesis of (5′*S*,6′*R*)-derivatives **14** and **15** was particularly straightforward and efficient, as their epoxy ester precursor **31** is readily accessible [44].

The primary goal of the reported study was to further elucidate MraY inhibition and not to obtain analogues with strong antibacterial potencies. It was anticipated that the target structures **10**–**17** would only show very weak antibacterial activities at best, as previously reported derivative **9** (which was a stronger MraY inhibitor) had already only been moderately active in inhibiting bacterial cell growth [24]. Indeed, **10**–**17** were devoid of activity against *E. coli* (MIC > 50 μg/mL). The comparison with **9** as well as with naturally occurring muraymycins [40] indicates that a low-nM (or stronger) inhibitory potency of this compound class against MraY appears to be a prerequisite for reasonable antibacterial activity. It needs to be taken into account that the polar muraymycin scaffold surely hampers cellular uptake, thus making it a decisive bottleneck for antibacterial potency. However, the novel variations of the muraymycin structure identified in this work will be highly useful for future SAR studies and the development of muraymycin analogues with improved MraY inhibition. In a second step, one can then aim to improve bacterial cellular uptake, for instance, by lipophilisation of the polar muraymycin scaffold. Such a strategy is envisioned to finally provide antibacterially active muraymycin analogues with improved chemical tractability as antimicrobial drug candidates.

## 4. Materials and Methods

### 4.1. Synthesis of Muraymycin Epimers

General methods: All chemicals were purchased from standard suppliers. Reactions involving oxygen and/or moisture sensitive reagents were carried out under an atmosphere of argon using anhydrous solvents. Anhydrous solvents were obtained in the following manner. THF was dried over sodium/benzophenone and distilled, CH_2_Cl_2_ was dried over CaH_2_ and distilled, MeOH was dried over activated molecular sieves (3 Å) and degassed. The obtained solvents were stored over molecular sieves (4 Å; in case of MeOH 3 Å). All other solvents were of technical quality and distilled prior to use, and deionised water was used throughout. Column chromatography was carried out on silica gel 60 (0.040–0.063 mm, 230–400 mesh ASTM, VWR, Darmstadt, Germany) under flash conditions except where indicated. TLC was performed on aluminium plates precoated with silica gel 60 F_254_ (VWR, Darmstadt, Germany). Visualisation of the spots was carried out using UV light (254 nm) and/or staining under heating (H_2_SO_4_ staining solution: 4 g vanillin, 25 mL conc. H_2_SO_4_, 80 mL AcOH, and 680 mL MeOH; KMnO_4_ staining solution: 1 g KMnO_4_, 6 g K_2_CO_3_, and 1.5 mL 1.25 M NaOH solution, all dissolved in 100 mL H_2_O; ninhydrin staining solution: 0.3 g ninhydrin, 3 mL AcOH, and 100 mL 1-butanol). Analytical HPLC was performed on a VWR-Hitachi system equipped with an L-2300 pump, an L-2200 autosampler, an L-2300 column oven (24 °C), an L-2455 Diode Array Detector (DAD), and a LiChroCart^TM^ column (4 × 125 mm) containing reversed phase silica gel Purospher^TM^ RP18e (5 μm) purchased from VWR (Darmstadt, Germany). The exact HPLC method is given in the Supplementary Materials. Preparative HPLC was carried out on a Jasco system equipped with a DG-2080-53 degasser, two PU-2080 Plus pumps, a UV-2075 Plus UV/Vis detector (detection at 260 nm), and a column (21 × 250 mm) containing reversed phase silica gel Nucleodur™ 100-5 C18ec (5 μm) purchased from Macherey-Nagel (Düren, Germany). Method: eluent A water (+0.1% TFA), eluent B 80:20 MeCN:water (+0.1% TFA); 0–30 min gradient of B (10–60%), 30–32 min gradient of B (60–100%), 32–40 min 100% B, 40–42 min gradient of B (100–5%), 42–51 min 5% B; flow 10 mL/min. 300 MHz- and 600 MHz-^1^H, and 75 MHz- and 126 MHz-^13^C, as well as 282 MHz-^19^F NMR spectra were recorded on Varian MERCURY 300, UNITY 300, and INOVA 600 spectrometers (Varian, Palo Alto, CA, USA). All ^13^C and ^19^F NMR spectra were ^1^H-decoupled. All spectra were recorded at room temperature except of samples in DMSO-*d*_6_ and D_2_O (standard 35 °C) and where indicated otherwise and were referenced internally to solvent reference frequencies wherever possible. Chemical shifts (δ) are quoted in ppm and coupling constants (*J*) are reported in Hz. Assignment of signals was carried out using H,H-COSY, HSQC, and HMBC spectra obtained on the spectrometers mentioned above. The numbering of atoms of muraymycin target structure is depicted in the Supplementary Material (Figure S20). Mass spectra of small molecules were measured on a Finnigan LCQ ion-trap mass spectrometer or on a Bruker microTOF spectrometer (Bremen, Germany). For ESI measurements in the negative mode, solutions of the compounds in pure MeOH were used, whereas for measurements in the positive mode, solutions in MeOH containing 0.1% formic acid were used. High resolution spectra were measured on a Bruker 7 Tesla Fourier transform ion cyclotron resonance (FTICR) mass spectrometer (Bruker, Bremen, Germany). Melting points (mp) were measured on a Büchi instrument (Büchi, Essen, Germany) and are not corrected. Optical rotations were recorded on a PerkinElmer polarimeter 241 (Perkin-Elmer, Hamburg, Germany) with a Na source using a 10 cm cell. Solutions of the compounds (~10 mg) in CHCl_3_, MeOH, or water (1 mL) were used, and concentrations are given in g/100 mL. Infrared spectroscopy (IR) was performed on a Jasco (Pfungstadt, Germany) FT/IR-4100 spectrometer equipped with an integrated ATR unit (GladiATR™, PIKE Technologies, Madison WI, USA). Wavenumbers (ν) are quoted in cm^−1^. UV spectroscopy of small molecules was carried out on a PerkinElmer (Hamburg, Germany) Lambda 2 spectrometer. Measurements were performed with solutions of ~0.1 mg of the compound in 10 mL MeCN, MeOH, or water and in the range of 190 to 500 nm. Wavelengths of maximum absorption (λ_max_) are reported in nm with the corresponding logarithmic molar extinction coefficient (log ε, ε/dm^3^ mol^−1^ cm^−1^) given in parenthesis.

5′-deoxy-l-Leu muraymycin analogue (**10**) and 5′-deoxy-d-Leu muraymycin analogue (**11**): To a solution of the urea tripeptide **18** (10 mg, 0.018 mmol) in THF (2.5 mL), 1-hydroxybenzotriazole (HOBt, 2.4 mg, 0.018 mmol), 1-ethyl-3-(3-dimethylaminopropyl)carbodiimide hydrochloride (ECD, 3.5 mg, 0.018 mmol), and *N*,*N*-diisopropylethylamine (DIPEA, 3.1 μL, 0.018 mmol) were added and the mixture was stirred at room temperature for 30 min. It was then added to a solution of amine **19** (15 mg, 0.023 mmol) in THF (2 mL) and stirred at room temperature for 20 h. EtOAc (30 mL) was added and the solution was washed with sat. NaHCO_3_ (30 mL). The organic layer was dried over Na_2_SO_4_ and the solvent was removed under reduced pressure. The protected analogues of **10** and **11** were obtained after column chromatography (98:2–95:5, CH_2_Cl_2_-MeOH) as colourless solids. This material was dissolved in TFA (80% in water, 2.7 mL) and stirred at room temperature for 24 h. Water (10 mL) was added and the solvent was removed under reduced pressure. The muraymycin analogues **10** (10 mg, 57%) and **11** (3.1 mg, 17%) were separated by preparative HPLC (**10**: *t*_R_ = 17.9 min, **11**: *t*_R_ = 16.5 min) and obtained as colourless solids. **10**: ^1^H NMR (600 MHz, D_2_O, 35 °C): δ [ppm] = 0.99 (d, *J* = 6.0 Hz, 3H, 5′′′-H_a_), 1.04 (d, *J* = 6.0 Hz, 3H, 5′′′-H_b_), 1.05 (d, *J* = 7.1 Hz, 3H, 4′′′′′-H_a_), 1.08 (d, *J* = 6.6 Hz, 3H, 4′′′′′-H_b_), 1.51–1.60 (m, 2H, 4′′′′-H), 1.66–1.83 (m, 6H, 3′′′-H, 4′′′-H, 3′′′′-H_a_, 5′′′′-H), 1.88–1.94 (m, 1H, 3′′′′-H_b_), 2.04 (dddd, *J* = 7.4, 7.2, 6.6, 6.4 Hz, 2H, 2′′-H), 2.28 (dqq, *J* = 7.1, 6.6, 6.2 Hz, 1H, 3′′′′′-H), 2.43 (ddd, *J* = 15.0, 9.7, 6.2 Hz, 1H, 5′-H_a_), 2.59 (ddd, *J* = 15.0, 6.7, 2.3 Hz, 1H, 5′-H_b_), 3.12 (dd, *J* = 7.6, 7.6 Hz, 2H, 6′′′′-H), 3.22 (dd, *J* = 7.4, 7.2 Hz, 2H, 1′′-H), 3.37 (ddd, *J* = 14.1, 6.6, 6.4 Hz, 1H, 3′′-H_a_), 3.44 (ddd, *J* = 14.1, 6.6, 6.4 Hz, 1H, 3′′-H_b_), 4.10 (dd, *J* = 6.7, 6.2 Hz, 1H, 6′-H), 4.20–4.23 (m, 2H, 3′-H, 2′′′′′-H), 4.24 (dd, *J* = 8.7, 5.4 Hz, 1H, 2′′′′-H), 4.29 (ddd, *J* = 9.7, 6.0, 2.3 Hz, 1H, 4′-H), 4.38 (dd, *J* = 10.8, 4.6 Hz, 1H, 2′′′-H), 4.56 (dd, *J* = 5.8, 4.0 Hz, 1H, 2′-H), 5.87 (d, *J* = 4.0 Hz, 1H, 1′-H), 6.01 (d, *J* = 8.1 Hz, 1H, 5-H), 7.76 (d, *J* = 8.1 Hz, 1H, 6-H). ^13^C NMR (126 MHz, D_2_O, 35 °C): δ [ppm] = 19.69 (C_a_-4′′′′′), 21.18 (C_b_-4′′′′′), 23.37 (C_a_-5′′′), 24.71 (C-4′′′′), 24.79 (C_b_-5′′′), 27.09 (C-4′′′), 28.30 (C-2′′), 28.93 (C-5′′′′), 32.64 (C-3′′′′′), 33.41 (C-3′′′′), 35.29 (C-5′), 38.64 (C-3′′), 41.92 (C-6′′′′), 42.23 (C-3′′′), 46.93 (C-1′′), 55.20 (C-2′′′), 56.75 (C-2′′′′), 61.50 (C-2′′′′′), 61.73 (C-6′), 75.18 (C-2′), 75.55 (C-3′), 82.46 (C-4′), 94.41 (C-1′), 104.84 (C-5), 118.93 (q, ^1^J_CF_ = 292.3 Hz, TFA-CF_3_), 145.32 (C-6), 153.89 (C-2), 161.95 (NC(=O)N), 165.36 (q, ^2^J_CF_ = 35.3 Hz, TFA-COO), 168.58 (C-4), 173.96 (C-7′), 177.32 (C-1′′′), 177.79 (C-1′′′′), 179.05 (C-1′′′′′). ^19^F NMR (282 MHz, D_2_O, 35 °C): δ [ppm] = −72.86 (TFA-CF_3_). MS (ESI^-^): *m*/*z* = 741.4 [M − H]^−^. HRMS (ESI)^−^: calcd.: 741.3788 [M − H]^−^, found: 741.3792. IR (ATR): *ν* = 1638, 1551, 1198, 1182, 1131, 799, 720, 551, 519. UV (H_2_O): *λ*_max_ (log ε) = 260 (4.01). Optical rotation: [α]_D_^25^ = −1.1 (c = 0.46, H_2_O). m.p. = 208 °C. **11**: ^1^H NMR (600 MHz, D_2_O, 35 °C): δ [ppm] = 0.98 (d, *J* = 6.1 Hz, 3H, 5′′′-H_a_), 1.04 (d, *J* = 5.9 Hz, 3H, 5′′′-H_b_), 1.04 (d, *J* = 6.9 Hz, 3H, 4′′′′′-H_a_), 1.08 (d, *J* = 6.7 Hz, 3H, 4′′′′′-H_b_), 1.50–1.63 (m, 2H, 4′′′′-H), 1.66–1.85 (m, 6H, 3′′′-H, 4′′′-H, 3′′′′-H_a_, 5′′′′-H), 1.87–1.93 (m, 1H, 3′′′′-H_b_), 1.97–2.07 (m, 2H, 2′′-H), 2.27 (dqq, *J* = 6.9, 6.7, 5.7 Hz, 1H, 3′′′′′-H), 2.36 (ddd, *J* = 15.1, 10.0 Hz, 6.6 Hz, 1H, 5′-H_a_), 2.54 (ddd, *J* = 15.1, 6.5 Hz, 3.0 Hz, 1H, 5′-H_b_), 3.11 (dd, *J* = 7.6, 7.6 Hz, 2H, 6′′′′-H), 3.16 (ddd, *J* = 13.2, 8.8, 7.9 Hz, 1H, 1′′-H_a_), 3.21 (ddd, *J* = 13.2, 8.0, 6.5 Hz, 1H, 1′′-H_b_), 3.38 (ddd, *J* = 14.2, 6.5, 6.4 Hz, 1H, 3′′-H_a_), 3.41 (ddd, *J* = 14.2, 7.0, 6.5 Hz, 1H, 3′′-H_b_), 3.93 (dd, *J* = 6.6, 6.5 Hz, 1H, 6′-H), 4.17 (d, *J* = 5.7 Hz, 1H, 2′′′′′-H), 4.20 (dd, *J* = 6.4, 5.9 Hz, 1H, 3′-H), 4.24 (dd, *J* = 8.2, 6.0 Hz, 1H, 2′′′′-H), 4.28 (ddd, *J* = 10.0, 6.4, 3.0 Hz, 1H, 4′-H), 4.35 (dd, *J* = 10.4, 4.1 Hz, 1H, 2′′′-H), 4.54 (dd, *J* = 5.9, 3.8 Hz, 1H, 2′-H), 5.88 (d, *J* = 3.8 Hz, 1H, 1′-H), 6.01 (d, *J* = 8.1 Hz, 1H, 5-H), 7.77 (d, *J* = 8.1 Hz, 1H, 6-H). ^13^C NMR (126 MHz, D_2_O, 35 °C): δ [ppm] = 19.74 (C_a_-4′′′′′), 21.23 (C_b_-4′′′′′), 22.99 (C_a_-5′′′), 24.78 (C-4′′′′), 24.94 (C_b_-5′′′), 27.13 (C-4′′′), 28.33 (C-2′′), 28.93 (C-5′′′′), 32.65 (C-3′′′′′), 33.50 (C-3′′′′), 35.77 (C-5′), 38.53 (C-3′′), 41.90 (C-6′′′′), 42.18 (C-3′′′), 46.87 (C-1′′), 55.21 (C-2′′′), 56.84 (C-2′′′′), 61.77 (C-2′′′′′), 62.91 (C-6′), 75.22 (C-2′), 75.51 (C-3′), 82.94 (C-4′), 94.32 (C-1′), 104.87 (C-5), 118.93 (q, ^1^J_CF_ = 292.3 Hz, TFA-CF_3_), 145.32 (C-6), 153.91 (C-2), 161.82 (NC(=O)N), 165.41 (q, ^2^J_CF_ = 34.0 Hz, TFA-COO), 168.59 (C-4), 174.75 (C-7′), 177.53 (C-1′′′), 178.10 (C-1′′′′), 179.42 (C-1′′′′′). ^19^F NMR (282 MHz, D_2_O, 35 °C): δ [ppm] = −72.87 (TFA-CF_3_). MS (ESI^+^): m/z = 743.5 [M + H]^+^. HRMS (ESI^+^): calcd.: 743.3934 [M + H]^+^, found: 743.3926. IR (ATR): *ν* [cm^−1^] = 1641, 1552, 1200, 1182, 1131, 799, 720, 550, 517. UV (H_2_O): *λ*_max_ (log ε) = 261 (4.07). Optical rotation: [α]_D_^25^ = +31.4 (c = 0.14, H_2_O). m.p. = 211 °C. 

5′-deoxy-6′-*epi*-l-Leu muraymycin analogue (**12**) and 5′-deoxy-6′-*epi*-d-Leu muraymycin analogue (**13**): To a solution of the urea tripeptide **18** (10 mg, 0.018 mmol) in THF (2.5 mL), HOBt (2.4 mg, 0.018 mmol), ECD (3.5 mg, 0.018 mmol), and DIPEA (3.1 μL, 0.018 mmol) were added and the mixture was stirred at room temperature for 30 min. It was then added to a solution of amine **20** (15 mg, 0.023 mmol) in THF (2 mL) and stirred at room temperature for 20 h. EtOAc (30 mL) was added and the solution was washed with sat. NaHCO_3_ (30 mL). The organic layer was dried over Na_2_SO_4_ and the solvent was removed under reduced pressure. The protected analogues of **12** and **13** were obtained after column chromatography (98:2–95:5, CH_2_Cl_2_-MeOH) as colourless solids. This material was dissolved in TFA (80% in water, 2.7 mL) and stirred at room temperature for 24 h. Water (10 mL) was added and the solvent was removed under reduced pressure. The muraymycin analogues **12** (10 mg, 57%) and **13** (3.3 mg, 19%) were separated by preparative HPLC (**12**: *t*_R_ = 17.8 min, **13**: *t*_R_ = 16.4 min) and obtained as colourless solids. **12**: ^1^H NMR (600 MHz, D_2_O, 35 °C): δ [ppm] = 0.99 (d, *J* = 5.8 Hz, 3H, 5′′′-H_a_), 1.04 (d, *J* = 5.4 Hz, 3H, 5′′′-H_b_), 1.05 (d, *J* = 6.7 Hz, 3H, 4′′′′′-H_a_), 1.08 (d, *J* = 6.8 Hz, 3H, 4′′′′′-H_b_), 1.51–1.61 (m, 2H, 4′′′′-H), 1.66–1.84 (m, 6H, 3′′′-H, 4′′′-H, 3′′′′-H_a_, 5′′′′-H), 1.88–1.94 (m, 1H, 3′′′′-H_b_), 1.96–2.04 (m, 2H, 2′′-H), 2.26 (dqq, *J* = 6.8, 6.7, 6.1 Hz, 1H, 3′′′′′-H), 2.47 (ddd, *J* = 15.5, 10.1, 4.8 Hz, 1H, 5′-H_a_), 2.59 (ddd, *J* = 15.5, 5.3, 2.9 Hz, 1H, 5′-H_b_), 3.12 (dd, *J* = 7.6, 7.5 Hz, 2H, 6′′′′-H), 3.16 (ddd, *J* = 12.2, 8.8, 7.6 Hz, 1H, 1′′-H_a_), 3.21 (ddd, *J* = 12.2, 8.6, 7.3 Hz, 1H, 1′′-H_b_), 3.35 (ddd, *J* = 14.3, 8.0, 6.7 Hz, 1H, 3′′-H_a_), 3.40 (ddd, *J* = 14.3, 7.5, 6.8 Hz, 1H, 3′′-H_b_), 4.08 (dd, *J* = 5.3, 4.8 Hz, 1H, 6′-H), 4.16 (ddd, *J* = 10.1, 7.9, 2.9 Hz, 1H, 4′-H), 4.21–4.25 (m, 3H, 3′-H, 2′′′′-H, 2′′′′′-H), 4.37 (dd, *J* = 9.7, 4.7 Hz, 1H, 2′′′-H), 4.56 (dd, *J* = 5.6, 3.8 Hz, 1H, 2′-H), 5.84 (d, *J* = 3.8 Hz, 1H, 1′-H), 6.00 (d, *J* = 8.1 Hz, 1H, 5-H), 7.78 (d, *J* = 8.1 Hz, 1H, 6-H). ^13^C NMR (126 MHz, D_2_O, 35 °C): δ [ppm] = 19.68 (C_a_-4′′′′′), 21.18 (C_b_-4′′′′′), 23.37 (C_a_-5′′′), 24.73 (C-4′′′′), 24.79 (C_b_-5′′′), 27.09 (C-4′′′), 28.21 (C-2′′), 28.94 (C-5′′′′), 32.66 (C-3′′′′′), 33.42 (C-3′′′′), 34.63 (C-5′), 38.65 (C-3′′), 41.92 (C-6′′′′), 42.23 (C-3′′′), 47.29 (C-1′′), 55.21 (C-2′′′), 56.77 (C-2′′′′), 61.51 (C-2′′′′′), 62.56 (C-6′), 75.00 (C-2′), 75.39 (C-3′), 82.26 (C-4′), 94.93 (C-1′), 104.85 (C-5), 118.93 (q, ^1^J_CF_ = 292.3 Hz, TFA-CF_3_), 145.71 (C-6), 153.92 (C-2), 161.94 (NC(=O)N), 165.37 (q, ^2^J_CF_ = 35.3 Hz, TFA-COO), 168.53 (C-4), 174.09 (C-7′), 177.31 (C-1′′′), 177.82 (C-1′′′′), 179.08 (C-1′′′′′). ^19^F NMR (282 MHz, D_2_O, 35 °C): δ [ppm] = −72.86 (TFA-CF_3_). MS (ESI^+^): *m*/*z* = 743.5 [M + H]^+^. HRMS (ESI^+^): calcd.: 743.3934 [M + H]^+^, found: 743.3926. IR (ATR): *ν* [cm^−1^] = 1633, 1551, 1198, 1182, 1131, 1054, 799, 720, 551. UV (H_2_O): *λ*_max_ (log ε) = 261 (4.00). Optical rotation: [α]_D_^25^ = −3.8 (c = 0.45, H_2_O). m.p. = 208 °C. **13**: ^1^H NMR (600 MHz, D_2_O, 35 °C): δ [ppm] = 0.99 (d, *J* = 5.6 Hz, 3H, 5′′′-H_a_), 1.05 (d, *J* = 6.1 Hz, 3H, 5′′′-H_b_), 1.05 (d, *J* = 7.1 Hz, 3H, 4′′′′′-H_a_), 1.08 (d, *J* = 6.6 Hz, 3H, 4′′′′′-H_b_), 1.49–1.63 (m, 2H, 4′′′′-H), 1.68–1.93 (m, 7H, 3′′′-H, 4′′′-H, 3′′′′-H, 5′′′′-H), 1.97–2.02 (m, 2H, 2′′-H), 2.27 (dqq, *J* = 7.1, 6.6, 5.8 Hz, 1H, 3′′′′′-H), 2.47 (ddd, *J* = 15.3, 10.1, 4.7 Hz, 1H, 5′-H_a_), 2.59 (ddd, *J* = 15.3, 5.3, 2.8 Hz, 1H, 5′-H_b_), 3.11 (dd, *J* = 8.0, 7.8 Hz, 2H, 6′′′′-H), 3.14 (ddd, *J* = 12.7, 7.7, 7.3 Hz, 1H, 1′′-H_a_), 3.20 (ddd, *J* = 12.3, 7.2, 7.0 Hz, 1H, 1′′-H_b_), 3.38 (dd, *J* = 6.7, 6.6 Hz, 2H, 3′′-H), 4.03 (dd, *J* = 5.3, 4.7 Hz, 1H, 6′-H), 4.16 (ddd, *J* = 10.1, 7.1, 2.8 Hz, 1H, 4′-H), 4.18 (d, *J* = 5.8 Hz, 1H, 2′′′′′-H), 4.24 (dd, *J* = 9.2, 6.3 Hz, 1H, 3′-H), 4.24 (dd, *J* = 5.9, 2.1 Hz, 1H, 2′′′′-H), 4.36 (dd, *J* = 10.3, 4.0 Hz, 1H, 2′′′-H), 4.55 (dd, *J* = 9.2, 3.8 Hz, 1H, 2′-H), 5.85 (d, *J* = 3.8 Hz, 1H, 1′-H), 6.00 (d, *J* = 8.1 Hz, 1H, 5-H), 7.79 (d, *J* = 8.1 Hz, 1H, 6-H). ^13^C NMR (126 MHz, D_2_O, 35 °C): δ [ppm] = 19.72 (C_a_ -4′′′′′), 21.19 (C_b_-4′′′′′), 23.00 (C_a_-5′′′), 24.79 (C-4′′′′), 24.93 (C_b_-5′′′), 27.14 (C-4′′′), 28.28 (C-2′′), 28.94 (C-5′′′′), 32.61 (C-3′′′′′), 33.48 (C-3′′′′), 34.72 (C-5′), 38.58 (C-3′′), 41.90 (C-6′′′′), 42.16 (C-3′′′), 47.30 (C-1′′), 55.21 (C-2′′′), 56.87 (C-2′′′′), 61.63 (C-2′′′′′), 62.96 (C-6′), 75.00 (C-2′), 75.41 (C-3′), 82.35 (C-4′), 94.78 (C-1′), 104.88 (C-5), 118.94 (q, ^1^J_CF_ = 291.1 Hz, TFA-CF_3_), 145.67 (C-6), 153.96 (C-2), 161.81 (NC(=O)N), 165.40 (q, ^2^J_CF_ = 35.3 Hz, TFA-COO), 168.55 (C-4), 174.33 (C-7′), 177.55 (C-1′′′), 178.10 (C-1′′′′), 179.23 (C-1′′′′′). ^19^F NMR (282 MHz, D_2_O, 35 °C): δ [ppm] = −72.87 (TFA-CF_3_). MS (ESI^+^): *m*/*z* = 743.5 [M + H]^+^. HRMS (ESI^+^): calcd.: 743.3934 [M + H]^+^, found: 743.3928. IR (ATR): *ν* [cm^−1^] = 1633, 1552, 1198, 1181, 1130, 1053, 720, 550, 518. UV (H_2_O): *λ*_max_ (log ε) = 260 (4.01). Optical rotation: [α]_D_^25^ = +28.7 (c = 0.15, H_2_O). m.p. = 209 °C. 

6′-*epi*-l-Leu muraymycin analogue (**14**) and 6′-*epi*-d-Leu muraymycin analogue (**15**): To a solution of the urea tripeptide **18** (13 mg, 0.024 mmol) in THF (3 mL), HOBt (3.2 mg, 0.024 mmol), EDC (4.6 mg, 0.024 mmol), and DIPEA (4.2 μL, 0.024 mmol) were added and the mixture was stirred at room temperature for 45 min. It was then added to a solution of amine **21** (20 mg, 0.030 mmol) in THF (2 mL) and stirred at room temperature for 18 h. EtOAc (30 mL) was added and the solution was washed with sat. NaHCO_3_ (30 mL). The organic layer was dried over Na_2_SO_4_ and the solvent was removed under reduced pressure. The protected analogues of **14** and **15** were obtained after column chromatography (98:2–96:4, CH_2_Cl_2_-MeOH) as colourless solids. This material was dissolved in TFA (80% in water, 3.6 mL) and stirred at room temperature for 24 h. Water (10 mL) was added and the solvent was removed under reduced pressure. The muraymycin analogues **14** (14 mg, 59%) and **15** (5.6 mg, 24%) were separated by preparative HPLC (**14**: *t*_R_ = 17.5 min, **15**: *t*_R_ = 16.1 min) and obtained as colourless solids. **14**: ^1^H NMR (600 MHz, D_2_O, 35 °C): δ [ppm] = 0.99 (d, *J* = 5.8 Hz, 3H, 5′′′-H_a_), 1.04 (d, *J* = 5.7 Hz, 3H, 5′′′-H_b_), 1.05 (d, *J* = 6.6 Hz, 3H, 4′′′′′-H_a_), 1.09 (d, *J* = 6.8 Hz, 3H, 4′′′′′-H_b_), 1.51–1.60 (m, 2H, 4′′′′-H), 1.66–1.85 (m, 6H, 3′′′-H, 4′′′-H, 3′′′′-H_a_, 5′′′′-H), 1.89–1.94 (m, 1H, 3′′′′-H_b_), 2.04 (dddd, *J* = 7.4, 7.3, 6.7, 6.5 Hz, 2H, 2′′-H), 2.28 (dqq, *J* = 6.8, 6.6, 6.4 Hz, 1H, 3′′′′′-H), 3.12 (dd, *J* = 7.7, 7.6 Hz, 2H, 6′′′′-H), 3.18 (ddd, *J* = 12.9, 7.4, 7.3 Hz, 1H, 1′′-H_a_), 3.25 (ddd, *J* = 12.9, 7.4, 7.3 Hz, 1H, 1′′-H_b_), 3.38 (ddd, *J* = 14.3, 6.5, 6.5 Hz, 1H, 3′′-H_a_), 3.45 (ddd, *J* = 14.3, 6.7, 6.7 Hz, 1H, 3′′-H_b_), 4.10 (d, *J* = 4.5 Hz, 1H, 6′-H), 4.22–4.25 (m, 3H, 4′-H, 2′′′′-H, 2′′′′′-H), 4.37 (dd, *J* = 9.6, 4.9 Hz, 1H, 2′′′-H), 4.46 (dd, *J* = 5.8, 5.3 Hz, 1H, 3′-H), 4.48 (dd, *J* = 5.3, 4.0 Hz, 1H, 2′-H), 4.58 (dd, *J* = 4.5, 1.1 Hz, 1H, 5′-H), 5.99 (d, *J* = 8.1 Hz, 1H, 5-H), 6.00 (d, *J* = 4.0 Hz, 1H, 1′-H), 8.14 (d, *J* = 8.1 Hz, 1H, 6-H). ^13^C NMR (126 MHz, D_2_O, 35 °C): δ [ppm] = 19.69 (C_a_-4′′′′′), 21.18 (C_b_-4′′′′′), 23.33 (C_a_-5′′′), 24.72 (C-4′′′′), 24.80 (C_b_-5′′′), 27.08 (C-4′′′), 27.81 (C-2′′), 28.93 (C-5′′′′), 32.69 (C-3′′′′′), 33.35 (C-3′′′′), 38.32 (C-3′′), 41.92 (C-6′′′′), 42.15 (C-3′′′), 48.22 (C-1′′), 55.23 (C-2′′′), 56.87 (C-2′′′′), 61.51 (C-2′′′′′), 68.51 (C-6′), 69.43 (C-5′), 72.48 (C-3′), 75.64 (C-2′), 85.67 (C-4′), 92.87 (C-1′), 104.74 (C-5), 118.93 (q, ^1^J_CF_ = 292.3 Hz, TFA-CF_3_), 145.06 (C-6), 154.07 (C-2), 161.96 (NC(=O)N), 165.37 (q, ^2^J_CF_ = 35.3 Hz, TFA-COO), 168.55 (C-4), 172.02 (C-7′), 177.43 (C-1′′′), 177.93 (C-1′′′′), 179.06 (C-1′′′′′). ^19^F NMR (282 MHz, D_2_O, 35 °C): δ [ppm] = −72.86 (TFA-CF_3_). MS (ESI^+^): *m*/*z* = 759.5 [M + H]^+^. HRMS (ESI^+^): calcd.: 759.3883 [M + H]^+^, found: 759.3886. IR (ATR): *ν* [cm^-1^] = 1632, 1549, 1198, 1182, 1128, 1049, 720, 560, 520. UV (H_2_O): *λ*_max_ (log ε) = 262 (3.94). Optical rotation: [α]_D_^25^ = +5.4 (c = 0.54, H_2_O). m.p. = 215 °C. **15**: ^1^H NMR (600 MHz, D_2_O, 35 °C): δ [ppm] = 0.98 (d, *J* = 5.3 Hz, 3H, 5′′′-H_a_), 1.04 (d, *J* = 5.3 Hz, 3H, 5′′′-H_b_), 1.04 (d, *J* = 6.5 Hz, 3H, 4′′′′′-H_a_), 1.08 (d, *J* = 6.8 Hz, 3H, 4′′′′′-H_b_), 1.49–1.54 (m, 1H, 4′′′′-H_a_), 1.56–1.62 (m, 1H, 4′′′′-H_b_), 1.66–1.92 (m, 7H, 3′′′-H, 4′′′-H, 3′′′′-H, 5′′′′-H), 1.98–2.07 (m, 2H, 2′′-H), 2.27 (dqq, *J* = 6.8, 6.5, 4.8 Hz, 1H, 3′′′′′-H), 3.11 (dd, *J* = 7.6, 7.6 Hz, 2H, 6′′′′-H), 3.18 (ddd, *J* = 13.7, 7.1, 6.0 Hz, 1H, 1′′-H_a_), 3.24 (ddd, *J* = 13.7, 7.3, 7.2 Hz, 1H, 1′′-H_b_), 3.37–3.43 (m, 2H, 3′′-H), 4.14 (d, *J* = 5.4 Hz, 1H, 6′-H), 4.19 (d, *J* = 4.8 Hz, 1H, 2′′′′′-H), 4.22 (dd, *J* = 6.1, 4.9 Hz, 1H, 2′′′′-H), 4.23 (dd, *J* = 5.8, 1.8 Hz, 1H, 4′-H), 4.35 (dd, *J* = 10.3, 4.1 Hz, 1H, 2′′′-H), 4.45 (dd, *J* = 5.8, 5.3 Hz, 1H, 3′-H), 4.48 (dd, *J* = 5.3, 4.1 Hz, 1H, 2′-H), 4.58 (dd, *J* = 5.4, 1.8 Hz, 1H, 5′-H), 5.98 (d, *J* = 8.1 Hz, 1H, 5-H), 5.99 (d, *J* = 4.1 Hz, 1H, 1′-H), 8.12 (d, *J* = 8.1 Hz, 1H, 6-H). ^13^C NMR (126 MHz, D_2_O, 35 °C): δ [ppm] = 19.69 (C_a_-4′′′′′), 21.18 (C_b_-4′′′′′), 23.00 (C_a_-5′′′), 24.79 (C-4′′′′), 24.93 (C_b_-5′′′), 27.13 (C-4′′′), 27.87 (C-2′′), 28.94 (C-5′′′′), 32.61 (C-3′′′′′), 33.47 (C-3′′′′), 38.23 (C-3′′), 41.90 (C-6′′′′), 42.14 (C-3′′′), 48.20 (C-1′′), 55.22 (C-2′′′), 56.88 (C-2′′′′), 61.53 (C-2′′′′′), 68.51 (C-6′), 69.43 (C-5′), 72.51 (C-3′), 75.58 (C-2′), 85.77 (C-4′), 92.84 (C-1′), 104.79 (C-5), 118.93 (q, ^1^J_CF_ = 292.3 Hz, TFA-CF_3_), 145.10 (C-6), 154.12 (C-2), 161.80 (NC(=O)N), 165.39 (q, ^2^J_CF_ = 36.5 Hz, TFA-COO), 168.57 (C-4), 171.97 (C-7′), 177.63 (C-1′′′), 178.09 (C-1′′′′), 179.16 (C-1′′′′′). ^19^F NMR (282 MHz, D_2_O, 35 °C): δ [ppm] = −72.87 (TFA-CF_3_). MS (ESI^+^): *m*/*z* = 759.5 [M + H]^+^. HRMS (ESI^+^): calcd.: 759.3883 [M+H]^+^, found: 759.3884. IR (ATR): *ν* [cm^−1^] = 1686, 1637, 1552, 1200, 1131, 721, 636, 568, 554. UV (H_2_O): *λ*_max_ (log ε) = 261 (4.08). Optical rotation: [α]_D_^25^ = +84.5 (c = 0.22, H_2_O). m.p. = 210 °C.

5′-*epi*-l-Leu muraymycin analogue (**16**) and 5′-*epi*-d-Leu muraymycin analogue (**17**): To a solution of the urea tripeptide **18** (6.7 mg, 0.012 mmol) in THF (1.5 mL), HOBt (1.6 mg, 0.012 mmol), ECD (2.3 mg, 0.012 mmol), and DIPEA (2.1 μL, 0.012 mmol) were added and the mixture was stirred at room temperature for 45 min. It was then added to a solution of amine **22** (10 mg, 0.015 mmol) in THF (3 mL) and stirred at room temperature for 18 h. EtOAc (30 mL) was added and the solution was washed with sat. NaHCO_3_ (30 mL). The organic layer was dried over Na_2_SO_4_ and the solvent was removed under reduced pressure. The protected analogues of **16** and **17** were obtained after column chromatography (98:2–96:4, CH_2_Cl_2_-MeOH) as colourless solids. This material was dissolved in TFA (80% in water, 1.8 mL) and stirred at room temperature for 24 h. Water (10 mL) was added and the solvent was removed under reduced pressure. The muraymycin analogues **16** (6.4 mg, 54%) and **17** (3.2 mg, 27%) were separated by preparative HPLC (**16**: *t*_R_ = 17.9 min, **17**: *t*_R_ = 16.3 min) and obtained as colourless solids. **16**: ^1^H NMR (600 MHz, D_2_O, 35 °C): δ [ppm] = 0.98 (d, *J* = 5.7 Hz, 3H, 5′′′-H_a_), 1.03 (d, *J* = 5.5 Hz, 3H, 5′′′-H_b_), 1.04 (d, *J* = 6.4 Hz, 3H, 4′′′′′-H_a_), 1.08 (d, *J* = 7.0 Hz, 3H, 4′′′′′-H_b_), 1.50–1.60 (m, 2H, 4′′′′-H), 1.66–1.83 (m, 6H, 3′′′-H, 4′′′-H, 3′′′′-H_a_, 5′′′′-H), 1.87–1.93 (m, 1H, 3′′′′-H_b_), 2.07 (dddd, *J* = 7.7, 7.6, 6.6, 6.5 Hz, 2H, 2′′-H), 2.28 (dqq, *J* = 7.0, 6.4, 5.7 Hz, 1H, 3′′′′′-H), 3.11 (dd, *J* = 7.7, 7.7 Hz, 2H, 6′′′′-H), 3.25 (dd, *J* = 7.7, 7.6 Hz, 2H, 1′′-H), 3.39 (ddd, *J* = 14.2, 6.6, 6.5 Hz, 1H, 3′′-H_a_), 3.48 (ddd, *J* = 14.2, 6.6, 6.5 Hz, 1H, 3′′-H_b_), 4.14 (d, *J* = 2.9 Hz, 1H, 6′-H), 4.22 (d, *J* = 5.7 Hz, 1H, 2′′′′′-H), 4.24 (dd, *J* = 8.7, 5.5 Hz, 1H, 2′′′′-H), 4.30 (dd, *J* = 8.4, 3.5 Hz, 1H, 4′-H), 4.39 (dd, *J* = 9.5, 4.7 Hz, 1H, 2′′′-H), 4.46 (dd, *J* = 8.4, 2.9 Hz, 1H, 5′-H), 4.49 (dd, *J* = 5.5, 3.5 Hz, 1H, 3′-H), 4.74 (dd, *J* = 5.9, 5.5 Hz, 1H, 2′-H), 5.84 (d, *J* = 5.9 Hz, 1H, 1′-H), 6.00 (d, *J* = 8.0 Hz, 1H, 5-H), 7.78 (d, *J* = 8.0 Hz, 1H, 6-H). ^13^C NMR (126 MHz, D_2_O, 35 °C): δ [ppm] = 19.68 (C_a_-4′′′′′), 21.18 (C_b_-4′′′′′), 23.39 (C_a_-5′′′), 24.69 (C-4′′′′), 24.76 (C_b_-5′′′), 27.09 (C-4′′′), 28.12 (C-2′′), 28.93 (C-5′′′′), 32.66 (C-3′′′′′), 33.45 (C-3′′′′), 38.74 (C-3′′), 41.92 (C-6′′′′), 42.23 (C-3′′′), 47.50 (C-1′′), 55.25 (C-2′′′), 56.73 (C-2′′′′), 61.48 (C-2′′′′′), 65.70 (C-6′), 71.73 (C-5′), 73.67 (C-3′), 74.33 (C-2′), 86.08 (C-4′), 94.17 (C-1′), 104.86 (C-5), 118.93 (q, ^1^J_CF_ = 291.1 Hz, TFA-CF_3_), 146.11 (C-6), 153.90 (C-2), 161.92 (NC(=O)N), 165.38 (q, ^2^J_CF_ = 35.3 Hz, TFA-COO), 168.59 (C-4), 171.19 (C-7′), 177.37 (C-1′′′), 177.76 (C-1′′′′), 179.05 (C-1′′′′′). ^19^F NMR (282 MHz, D_2_O, 35 °C): δ [ppm] = −72.88 (TFA-CF_3_). MS (ESI^+^): *m*/*z* = 759.5 [M + H]^+^. HRMS (ESI^+^): calcd.: 759.3883 [M + H]^+^, found: 759.3884. IR (ATR): *ν* [cm^−1^] = 1660, 1633, 1551, 1197, 1184, 1132, 720, 547, 511. UV (H_2_O): *λ*_max_ (log ε) = 260 (3.98). Optical rotation: [α]_D_^25^ = -10.6 (c = 0.16, H_2_O). m.p. = 214 °C. **17**: ^1^H NMR (600 MHz, D_2_O, 35 °C): δ [ppm] = 0.98 (d, *J* = 5.3 Hz, 3H, 5′′′-H_a_), 1.00 (d, *J* = 6.8 Hz, 3H, 5′′′-H_b_), 1.04 (d, *J* = 6.0 Hz, 3H, 4′′′′′-H_a_), 1.05 (d, *J* = 6.8 Hz, 3H, 4′′′′′-H_b_), 1.51–1.63 (m, 2H, 4′′′′-H), 1.69–1.87 (m, 6H, 3′′′-H, 4′′′-H, 3′′′′-H_a_, 5′′′′-H), 1.88–1.94 (m, 1H, 3′′′′-H_b_), 2.07 (dddd, *J* = 7.3, 7.2, 6.6, 6.3 Hz, 2H, 2′′-H), 2.21 (dqq, *J* = 6.8, 6.0, 5.9 Hz, 1H, 3′′′′′-H), 3.12 (dd, *J* = 7.6, 7.4 Hz, 2H, 6′′′′-H), 3.20–3.26 (m, 2H, 1′′-H), 3.39 (ddd, *J* = 14.3, 6.6, 6.3 Hz, 1H, 3′′-H_a_), 3.47 (ddd, *J* = 14.3, 6.6, 6.3 Hz, 1H, 3′′-H_b_), 3.95 (d, *J* = 3.0 Hz, 1H, 6′-H), 4.06 (d, *J* = 5.9 Hz, 1H, 2′′′′′-H), 4.23 (dd, *J* = 7.3, 7.1 Hz, 1H, 2′′′′-H), 4.29 (dd, *J* = 7.6, 4.4 Hz, 1H, 4′-H), 4.38 (dd, *J* = 9.5, 4.1 Hz, 1H, 2′′′-H), 4.44 (dd, *J* = 7.6, 3.0 Hz, 1H, 5′-H), 4.49 (dd, *J* = 5.3, 4.4 Hz, 1H, 3′-H), 4.65 (dd, *J* = 5.6, 5.3 Hz, 1H, 2′-H), 5.89 (d, *J* = 5.6 Hz, 1H, 1′-H), 6.01 (d, *J* = 8.1 Hz, 1H, 5-H), 7.83 (d, *J* = 8.1 Hz, 1H, 6-H). ^13^C NMR (126 MHz, D_2_O, 35 °C): δ [ppm] = 19.77 (C_a_-4′′′′′), 21.56 (C_b_-4′′′′′), 22.98 (C_a_-5′′′), 24.79 (C-4′′′′), 24.95 (C_b_-5′′′), 27.12 (C-4′′′), 28.13 (C-2′′), 28.98 (C-5′′′′), 33.04 (C-3′′′′′), 33.38 (C-3′′′′), 38.77 (C-3′′), 41.92 (C-6′′′′), 42.17 (C-3′′′), 47.46 (C-1′′), 55.19 (C-2′′′), 56.84 (C-2′′′′), 63.09 (C-2′′′′′), 66.68 (C-6′), 71.79 (C-5′), 73.11 (C-3′), 74.83 (C-2′), 86.09 (C-4′), 94.45 (C-1′), 104.88 (C-5), 118.89 (q, ^1^J_CF_ = 291.1 Hz, TFA-CF_3_), 145.66 (C-6), 154.01 (C-2), 161.81 (NC(=O)N), 165.39 (q, ^2^J_CF_ = 37.8 Hz, TFA-COO), 168.63 (C-4), 171.91 (C-7′), 177.01 (C-1′′′), 177.54 (C-1′′′′), 178.33 (C-1′′′′′). ^19^F NMR (282 MHz, D_2_O, 35 °C): δ [ppm] = –72.88 (TFA-CF_3_). MS (ESI^+^): *m*/*z* = 759.5 [M + H]^+^. HRMS (ESI^+^): calcd.: 759.3883 [M + H]^+^, found: 759.3888. IR (ATR): *ν* [cm^−1^] = 1667, 1634, 1552, 1201, 1132, 1056, 800, 721, 547. UV (H_2_O): *λ*_max_ (log ε) = 260 (3.95). optical rotation: [α]_D_^25^ = +13.1 (c = 0.13, H_2_O). m.p. = 218 °C.

Protected urea tripeptide (**18**): To a solution of **29** (50 mg, 0.076 mmol) in THF (4 mL), tetrabutylammonium fluoride (TBAF, 1 m in THF, 91 μL, 0.091 mmol) was added at 0 °C and the mixture was stirred at 0 °C for 1 h. More TBAF (1 m in THF, 30 μL, 0.030 mmol) was added and the mixture was stirred at room temperature for 3 h. The solvent was removed under reduced pressure. The urea tripeptide **18** was obtained after column chromatography (95:4:1, CH_2_Cl_2_-MeOH-HOAc) as colourless solid (39 mg, 92%). ^1^H NMR (600 MHz, DMSO-d_6_, 35 °C): δ [ppm] = 0.83 (d, *J* = 6.9 Hz, 3H, 4-H_a_), 0.83 (d, *J* = 6.5 Hz, 3H, 5′′-H_a_), 0.85 (d, *J* = 6.8 Hz, 3H, 4-H_b_), 0.88 (d, *J* = 6.6 Hz, 3H, 5′′-H_b_), 1.22–1.28 (m, 2 H, 4′-H), 1.35–1.44 (m, 3H, 3′-H_a_, 5′′-H), 1.38 (s, 9H, OC(CH_3_)_3_), 1.40 (s, 9H, OC(CH_3_)_3_), 1.52 (ddd, *J* = 9.1, 5.3, 5.0 Hz, 2H, 3′′-H), 1.53–1.58 (m, 1H, 3′-H_b_), 1.57.1.65 (m, 1H, 4′′-H), 1.96 (dqq, *J* = 6.9, 6.8, 5.1 Hz, 1H, 3-H), 2.84–2.88 (m, 2H, 6′-NH), 3.92 (dd, *J* = 8.6, 5.1 Hz, 1H, 2-H), 4.14 (ddd, *J* = 8.2, 7.7, 5.3 Hz, 1H, 2′-H), 4.20 (ddd, *J* = 9.1, 8.0, 5.6 Hz, 1H, 2′′-H), 6.25 (d, *J* = 8.6 Hz, 1H, 2-NH), 6.27 (d, *J* = 7.7 Hz, 1H, 2′-NH), 6.66 (dd, *J* = 6.0, 5.8 Hz, 1H, 6′-NH), 8.01 (d, *J* = 8.0 Hz, 1H, 2′′-NH). ^13^C NMR (126 MHz, DMSO-d_6_, 35 °C): δ [ppm] = 17.59 (C_a_-4), 18.92 (C_b_-4), 21.33 (C_a_-5′′), 22.24 (C-4′), 24.79 (C_b_-5′′), 24.17 (C-4′′), 27.64 (OC(CH_3_)_3_), 28.20 (OC(CH_3_)_3_), 29.30 (C-5′), 30.43 (C-3), 32.97 (C-3′), 39.76 (C-6′), 39.92 (C-3′′), 50.09 (C-2′′), 52.41 (C-2′), 58.10 (C-2), 77.13 (OC(CH_3_)_3_), 80.04 (OC(CH_3_)_3_), 155.25 (NC(=O)O), 157.09 (NC(=O)N), 171.36 (C-1), 172.09 (C-1′), 173.64 (C-1′′). MS (ESI^+^): *m*/*z* = 581.3 [M + Na]^+^. HRMS (ESI^+^): calcd.: 581.3521 [M + Na]^+^, found: 581.3522. IR (ATR): *ν* [cm^−1^] = 1719, 1688, 1633, 1546, 1391, 1366, 1250, 1156, 665. Optical rotation: [α]_D_^25^ = −3.3 (c = 0.24, CHCl_3_). m.p. = 73 °C. TLC: R*_f_* = 0.25 (94:5:1, CH_2_Cl_2_-MeOH-AcOH).

6′-*epi* nucleoside building block (**21**): To a solution of **33** (45 mg, 0.057 mmol) in MeOH (4 mL), Pd/C (10%, 10 mg, 9.4 μmol) and 1,4-cyclohexadiene (54 μL, 0.57 mmol) were added and the mixture was stirred at room temperature for 2 h. More Pd/C (10%, 5 mg, 5 μmol) and 1,4-cyclohexadiene (54 μL, 0.57 mmol) were added and the mixture was stirred at room temperature for 1 h. The mixture was filtered and the residue was washed with MeOH (3 × 4 mL). The solvent of the combined filtrates was removed under reduced pressure to give **21** as a colourless solid (37 mg, 99%). ^1^H NMR (600 MHz, CD_3_OD): δ [ppm] = 0.05 (s, 3H, SiCH_3_), 0.07 (s, 3H, SiCH_3_), 0.12 (s, 3H, SiCH_3_), 0.14 (s, 3H, SiCH_3_), 0.88 (s, 9H, SiC(CH_3_)_3_), 0.94 (s, 9H, SiC(CH_3_)_3_), 1.49 (s, 9H, OC(CH_3_)_3_), 1.66–1.75 (m, 2H, 2′′-H), 2.63 (ddd, *J* = 12.3, 6.2, 5.9 Hz, 1H, 1′′-H_a_), 2.76 (ddd, *J* = 12.3, 7.2, 5.6 Hz, 1H, 1′′-H_b_), 2.94–2.96 (m, 2H, 3′′-H), 3.38 (d, *J* = 7.4 Hz, 1 H, 6′-H), 3.87 (dd, *J* = 7.4, 1.2 Hz, 1H, 5′-H), 4.17 (dd, *J* = 4.2, 3.7 Hz, 1H, 3′-H), 4.20 (dd, *J* = 3.7, 1.2 Hz, 1H, 4′-H), 4.36 (dd, *J* = 5.3, 4.2 Hz, 1H, 2′-H), 5.72 (d, *J* = 8.1 Hz, 1H, 5-H), 5.88 (d, *J* = 5.3 Hz, 1H, 1′-H), 8.18 (d, *J* = 8.1 Hz, 1H, 6-H). ^13^C NMR (126 MHz, CD_3_OD): δ [ppm] = −4.58 (SiCH_3_), −4.49 (SiCH_3_), −4.49 (SiCH_3_), −4.22 (SiCH_3_), 18.76 (SiC(CH_3_)_3_), 18.85 (SiC(CH_3_)_3_), 26.24 (SiC(CH_3_)_3_), 26.31 (SiC(CH_3_)_3_), 28.29 (OC(CH_3_)_3_), 29.82 (C-2′′), 40.38 (C-3′′), 46.92 (C-1′′), 65.99 (C-6′), 71.28 (C-5′), 74.41 (C-3′), 76.01 (C-2′), 82.79 (OC(CH_3_)_3_), 86.15 (C-4′), 90.00 (C-1′), 102.72 (C-5), 142.68 (C-6), 152.45 (C-2), 166.11 (C-4), 174.08 (C-7′). MS (ESI^+^): *m*/*z* = 659.4 [M + H]^+^. HRMS (ESI^+^): calcd.: 659.3866 [M + H]^+^, found: 659.3867. IR (ATR): *ν* [cm^−1^] = 1686, 1253, 1153, 1113, 1051, 869, 834, 812, 773. UV (MeOH): *λ*_max_ (log ε) = 207 (3.98), 262 (3.98). Optical rotation: [α]_D_^25^ = +24.0 (c = 0.30, MeOH). m.p. = 115 °C.

5′-*epi* nucleoside building block (**22**): To a solution of **34** (5.0 mg, 6.3 μmol) in MeOH (4 mL), Pd/C (10%, 5 mg, 5 μmol), and 1,4-cyclohexadiene (6.3 μL, 0.063 mmol) were added and the mixture was stirred at room temperature for 30 min. More Pd/C (10%, 5 mg, 5 μmol) was added and the mixture was stirred at room temperature for 30 min. The mixture was filtered and the residue was washed with MeOH (3 × 4 mL). The solvent of the combined filtrates was removed under reduced pressure to give **22** as a colourless solid (4.1 mg, 99%). ^1^H NMR (600 MHz, pyridine-d_5_, 35 °C): δ [ppm] = 0.14 (s, 3H, SiCH_3_), 0.16 (s, 3H, SiCH_3_), 0.26 (s, 3H, SiCH_3_), 0.35 (s, 3H, SiCH_3_), 0.94 (s, 9H, SiC(CH_3_)_3_), 1.06 (s, 9H, SiC(CH_3_)_3_), 1.59 (s, 9H, OC(CH_3_)_3_), 2.11–2.16 (m, 1H, 2′′-H_a_), 2.21–2.26 (m, 1H, 2′′-H_b_), 3.09 (ddd, *J* = 12.3, 7.2, 5.6 Hz, 1H, 1′′-H_a_), 3.22 (ddd, *J* = 12.3, 6.1, 6.1 Hz, 1H, 1′′-H_b_), 3.45 (ddd, *J* = 12.5, 6.8, 6.7 Hz, 1H, 3′′-H_a_), 3.50 (ddd, *J* = 12.5, 6.6 Hz, 6.5 Hz, 1H, 3′′-H_b_), 4.06 (d, *J* = 3.8 Hz, 1H, 6′-H), 4.68 (d, *J* = 7.9 Hz, 1H, 4′-H), 4.79 (d, *J* = 4.3 Hz, 1H, 3′-H), 4.88 (dd, *J* = 7.9, 3.8 Hz, 1H, 5′-H), 5.12 (dd, *J* = 7.9, 4.3 Hz, 1H, 2′-H), 6.03 (d, *J* = 8.1 Hz, 1H, 5-H), 6.88 (d, *J* = 7.9 Hz, 1H, 1′-H), 8.41 (d, *J* = 8.1 Hz, 1H, 6-H). ^13^C NMR (126 MHz, pyridine-d_5_, 35 °C): δ [ppm] = −4.46 (SiCH_3_), −4.05 (SiCH_3_), −4.00 (SiCH_3_), −3.95 (SiCH_3_), 18.32 (SiC(CH_3_)_3_), 18.51 (SiC(CH_3_)_3_), 26.16 (SiC(CH_3_)_3_), 26.26 (SiC(CH_3_)_3_), 27.75 (C-2′′), 28.41 (OC(CH_3_)_3_), 39.51 (C-3′′), 46.86 (C-1′′), 64.60 (C-6′), 73.58 (C-5′), 73.85 (C-2′), 74.03 (C-3′), 81.72 (OC(CH_3_)_3_), 87.19 (C-4′), 87.19 (C-1′), 103.29 (C-5), 142.71 (C-6), 152.37 (C-2), 164.15 (C-4), 171.56 (C-7′). MS (ESI^+^): *m*/*z* = 659.4 [M + H]^+^. HRMS (ESI^+^): calcd.: 659.3866 [M + H]^+^, found: 659.3871. IR (ATR): *ν* [cm^−1^] = 1678, 1252, 1155, 1057, 865, 833, 813, 776, 542. UV (MeOH): *λ*_max_ (log ε) = 205 (3.98), 260 (3.84). Optical rotation: [α]_D_^25^ = −32.1 (c = 0.42, MeOH). m.p. = 184 °C. 

*N*^ε^-*tert*-butyloxycarbonyl-l-lysine trimethylsilylethyl ester (**24**): To a solution of *N*^α^-benzyloxycarbonyl-*N*^ε^-*tert*-butyloxycarbonyl-l-lysine **23** (667 mg, 1.75 mmol), ECD (673 mg, 3.51 mmol), and 4-(dimethylamino)pyridine (DMAP, 175 mg, 1.43 mmol) in CH_2_Cl_2_ (8.9 mL), 2-(trimethylsilyl)ethanol (380 μL, 310 mg, 2.62 mmol) was added and the mixture was stirred at room temperature for 25 h. It was then washed with sat. NaHCO_3_ (3 × 30 mL), brine (3 × 30 mL), and water (60 mL). The organic layer was dried over Na_2_SO_4_ and the solvent was removed under reduced pressure. The resultant crude product was purified by column chromatography (6:4, petroleum ether-CH_2_Cl_2_) to give the *N*^α^-protected lysine ester as a colourless oil (653 mg, 77%). This material (600 mg, 1.25 mmol) was dissolved in MeOH (2 mL), Pd/C (10%, 190 mg, 0.179 mmol) was added and the mixture was stirred under a hydrogen atmosphere (1 bar) at room temperature for 3 h. It was then filtered over celiter^TM^, the residue was washed with MeOH and the solvent of the combined filtrates was removed under reduced pressure to give **24** as a colourless oil (422 mg, 99%, 77% over 2 steps from **23**). ^1^H NMR (300 MHz, CD_2_Cl_4_, 100 °C): δ [ppm] = 4.53–4.44 (m, 1H, N^ε^H), 4.26–4.20 (m, 2H, H-1′), 3.39–3.35 (m, 1H, H-2), 3.14–3.07 (m, 2H, H-6), 1.80–1.68 (m, 1H, H-3_a_), 1.59–1.39 (m, 5H, H-3_b_, H-4, H-5), 1.45 (s, 9H, Boc-CH_3_), 1.05–1.00 (m, 2H, H-2′), 0.08 (s, 9H, Si(CH_3_)_3_). ^13^C-NMR (75 MHz, CD_2_Cl_4_, 100 °C): δ [ppm] = 175.38 (C-1), 155.57 (Boc-C=O), 78.67 (Boc-C), 62.70 (C-1′), 54.33 (C-2), 40.48 (C-6), 34.25 (C-3), 29.64 (C-5), 28.23 (Boc-CH_3_), 22.71 (C-4), 17.34 (C-2′), -1.77 (Si(CH_3_)_3_). MS (ESI^+^): *m*/*z* = 347.2 [M + H]^+^. HRMS (ESI^+^): calcd.: 347.2361 [M + H]^+^, found: 347.2364. IR (ATR): *ν* [cm^−1^] = 3372, 2952, 2364, 1713, 1520, 1365, 1250, 1171, 838. Optical rotation: [α]_D_^25^ = +5.5 (c = 0.58, CHCl_3_). TLC: R*_f_* (95:5, CH_2_Cl_2_-MeOH) = 0.80.

Protected urea dipeptide trimethylsilylethyl ester (**26**): To a solution of **24** (10 mg, 29 μmol) and *N*-(*S*-methylthiocarbonyl)-l-valine *tert*-butyl ester **25** (7.9 mg, 32 μmol) in EtOAc (1 mL), *N*-methylmorpholine (NMM, 9.5 μL, 8.7 mg, 86 μmol) and silver(I)-trifluoromethanesulfonate (AgOTf, 11 mg, 43 μmol) were added and the mixture was stirred at room temperature for 17 h. The solvent was removed under reduced pressure, and the resultant crude product was purified by column chromatography (3:1, petroleum ether-EtOAc) to give **26** as colourless oil (12 mg, 76%). ^1^H NMR (300 MHz, CD_2_Cl_4_, 100 °C): δ [ppm] = 4.98 (d, *J* = 8.0 Hz, 1H, Lys-N^α^H), 4.95 (d, *J* = 8.8 Hz, 1H, Val-NH), 4.63–4.48 (m, 1H, Lys-N^ε^H), 4.38 (ddd, *J* = 8.0, 7.6, 5.5 Hz, 1H, Lys-H-2), 4.31–4.14 (m, 3H, Val-H-2, H-1), 3.10 (dd, *J* = 13.0, 6.7 Hz, 2H, Lys-H-6), 2.18–2.03 (m, 1H, Val-H-3), 1.89–1.75 (m, 1H, Lys-H-3_a_), 1.75–1.59 (m, 1H, Lys-H-3_b_), 1.59–1.32 (m, 4H, Lys-H-4, Lys-H-5), 1.49 (s, 9H, *t*-Bu-CH_3_) 1.46 (s, 9H, Boc-CH_3_), 1.08–0.99 (m, 2H, H-2), 0.97 (d, *J* = 9.5 Hz, 3H, Val-H-4), 0.94 (d, *J* = 9.5 Hz, 3H, Val-H-4), 0.08 (s, 9H, Si(CH_3_)_3_). ^13^C NMR (75 MHz, CD_2_Cl_4_, 100 °C): δ [ppm] = 172.89 (Lys-C-1), 171.56 (Val-C-1), 156.78 (Boc-C=O), 155.69 (urea-C=O), 81.42 (*t*-Bu-C), 78.71 (Boc-C), 63.24 (C-1), 58.68 (Val-C-2), 53.13 (Lys-C-2), 40.35 (Lys-C-6), 32.33 (Lys-C-3), 31.16 (Val-C-3), 29.46 (Lys-C-5), 28.26 (*t*-Bu-CH_3_), 27.90 (Boc-CH_3_), 22.35 (Lys-C-4), 18.55 (Val-C-4), 17.60 (Val-C-4), 17.32 (C-2), -1.80 (Si(CH_3_)_3_). MS (ESI^+^): *m*/*z* = 568.3 [M + Na]^+^. HRMS (ESI)^+^: calcd.: 568.3388 [M + Na]^+^, found: 568.3391. IR (ATR): *ν* [cm^−1^] = 3355, 2961, 1715, 1644, 1550, 1365, 1249, 1164, 836. Optical rotation: [α]_D_^25^ = 7.7 (c = 0.38, CHCl_3_). TLC: R*_f_* (3:2, petroleum ether-EtOAc) = 0.47.

Protected urea dipeptide (**27**): To a solution of **26** (380 mg, 0.696 mmol) in THF (8.7 mL), tetrabutylammoniumfluoride solution (TBAF, 1 m in THF, 840 μL, 0.840 mmol) was added at 0 °C and the mixture was stirred at room temperature for 5 h. The solvent was removed under reduced pressure, and the resultant crude product was purified by column chromatography (95:5:1, CH_2_Cl_2_-MeOH-HOAc) to give **27** as colourless oil (287 mg, 93%). ^1^H NMR (300 MHz, CD_2_Cl_4_, 100 °C): δ [ppm] = 5.69–5.37 (m, 2H, Lys-N^α^H, Val-N^α^H). 4.95–4.83 (m, 1H, Lys-N^ε^H), 4.36–4.28 (m, 1H, Lys-H-2), 4.25–4.18 (m, 1H, Val-H-2), 3.16–3.06 (m, 2H, Lys-H-6), 2.23–2.02 (m, 1H, Val-H-3), 1.95–1.80 (m, 1H, Lys-H-3_a_), 1.80–1.66 (m, 1H, Lys-H-3_b_), 1.51–1.48 (m, 4H, Lys-H-4, Lys-H-5), 1.48 (s, 9H, *t*-Bu-CH_3_) 1.47 (s, 9H, Boc-CH_3_), 0.97 (d, *J* = 10.5 Hz, 3H, Val-H-4), 0.94 (d, *J* = 10.5 Hz, 3H, Val-H-4). ^13^C NMR (75 MHz, CD_2_Cl_4_, 100 °C): δ [ppm] = 175.12 (Lys-C-1) 171.99 (Val-C-1), 158.34 (Boc-C=O), 156.21 (urea-C=O), 81.98 (*t*-Bu-C), 81.74 (Boc-C), 58.46 (Val-C-2), 53.38 (Lys-C-2), 40.04 (Lys-C-6), 31.16 (Lys-C-3), 31.11 (Val-C-3), 29.29 (Lys-C-5), 28.26 (*t*-Bu-CH_3_), 27.85 (Boc-CH_3_), 22.25 (Lys-C-4), 18.69 (Val-C-4), 17.43 (Val-C-4). MS (ESI^+^): *m*/*z* = 468.3 [M + Na]^+^. HRMS (ESI^+^): calcd.: 468.2680 [M + Na]^+^, found: 468.2681. IR (ATR): *ν* [cm^−1^] = 3359, 2967, 1715, 1640, 1552, 1366, 1159, 847. Optical rotation: [α]_D_^25^ = 19.9 (c = 0.80, CHCl_3_). TLC: R*_f_* (7:3:1, CH_2_Cl_2_:MeOH:HOAc) = 0.41.

l*-*leucine trimethylsilylethyl ester trifluoroacetate (**28**): To a solution of *N*-benzyloxycarbonyl-l-leucine **30** (10.0 g, 37.8 mmol) in CH_2_Cl_2_ (385 mL), EDC (9.21 g, 48 mmol), DMAP (922 mg, 7.55 mmol), and 2-(trimethylsilyl)ethanol (6.87 mL, 5.67 g, 48.0 mmol) were added and the mixture was stirred at room temperature for 14 h. It was then washed with HCl (1 m, 3 × 600 mL), sat. NaHCO_3_ (3 × 600 mL), and brine (3 × 600 mL). The organic layer was dried over NaSO_4_ and the solvent was removed under reduced pressure. *N*-benzyloxycarbonyl-l-leucine trimethylsilylethyl ester was isolated after column chromatography as a colourless oil (11.8 g, 86%). To a solution of this material (366 mg, 1.00 mmol) in MeOH (40 mL), TFA (80 μL, 0.12 g, 1.0 mmol) and Pd/C (10%, 146 mg, 0.130 mmol) were added and the mixture was stirred under hydrogen atmosphere (1 bar) at room temperature for 4 h. It was then filtered over celite™ and the residue was washed with MeOH. The solvent of the combined filtrates was removed under reduced pressure to give **28** as a colourless solid (345 mg, quant., 86% over 2 steps from **30**). ^1^H NMR (300 MHz, CDCl_3_): δ [ppm] = 4.29–4.23 (m, 2H, H-1′), 3.90 (dd, *J* = 7.0, 7.0 Hz, 1H, H-2), 1.87–1.73 (m, 3H, H-3, H-4), 1.05–0.99 (m, 2H, H-2′), 0.97 (d, *J* = 6.1 Hz, 3H, H-5), 0.95 (d, *J* = 6.1 Hz, 3H, H-5), 0.04 (s, 9H, Si(CH_3_)_3_). ^13^C NMR (75 MHz, CDCl_3_): δ [ppm] = 170.12 (C-1), 162.73–161.77 (m, TFA-COO), 116.32–114.45 (m, TFA-CF_3_), 65.12 (C-1′), 51.54 (C-2), 39.61 (C-3), 24.29 (C-4), 22.09 (C-5), 21.74 (C-5), 17.19 (C-2′), −1.68 (Si(CH_3_)_3_). ^19^F NMR (282 MHz, CDCl_3_): δ [ppm] = −75.98 (TFA-CF_3_). MS (ESI^+^): *m*/*z* = 232.2 [M − TFA]^+^. HRMS (ESI^+^): calcd.: 232.1727 [M − TFA]^+^, found: 232.1724. IR (ATR): *ν* [cm^−1^] = 1733, 1665, 1250, 1201, 1174, 1137, 1042, 930, 834. Optical rotation: [α]_D_^25^ = 3.6 (c = 1.0, CHCl_3_). m.p. = 85 °C. TLC: R*_f_* (9:1, petroleum ether:EtOAc) = 0.76.

Protected urea tripeptide trimethylsilylethyl ester (**29**): To a solution of **27** (15 mg, 35 μmol) in THF (1 mL), HOBt (4.7 mg, 35 μmol), and EDC (6.7 mg, 35 μmol) were added and the mixture was stirred at room temperature for 30 min. A solution of **28** (12 mg, 35 μmol) and DIPEA (12 μL, 9.0 mg, 70 μmol) in CH_2_Cl_2_ (1 mL) was added and the mixture was stirred at room temperature for 18 h. The solvent was removed under reduced pressure, and the resultant crude product was purified by column chromatography (99:1, CH_2_Cl_2_-MeOH) to give **29** as a colourless solid (18 mg, 80%). ^1^H NMR (300 MHz, CD_2_Cl_4_, 100 °C): δ [ppm] = 6.59 (d, *J* = 8.1 Hz, 1H, Lys-N^α^H), 5.21 (d, *J* = 7.8 Hz, 1H, Leu-NH), 5.14 (d, *J* = 8.7 Hz, 1H, Val-NH), 4.76–4.64 (m, 1H, Lys-N^ε^H), 4.53 (ddd, *J* = 8.2, 8.1, 5.2 Hz, 1H, Lys-H-2), 4.31–4.12 (m, 4H, Val-H-2, Leu-H-2, H-1), 3.10 (dd, *J* = 13.1, 6.3 Hz, 2H, Lys-H-6), 2.20–2.02 (m, 1H, Val-H-3), 1.93–1.76 (m, 1H, Lys-H-3_a_), 1.77–1.34 (m, 8H, Lys-H-3_b_, Lys-H-4, Lys-H-5, Leu-H-3, Leu-H-4), 1.46 (s, 9H, Boc-CH_3_), 1.49 (s, 9H, *t*-Bu-CH_3_), 1.10–1.00 (m, 2H, H-2), 1.01–0.87 (m, 12H, 2 × Val-H-4, 2 × Leu-H-5), 0.07 (s, 9H, Si(CH_3_)_3_). ^13^C NMR (75 MHz, CD_2_Cl_4_, 100 °C): δ [ppm] = 172.41 (Lys-C-1), 172.36 (Val-C-1), 172.06 (Leu-C-1), 157.52 (Boc-C=O), 155.97 (urea-C=O), 81.55 (*t*-Bu-C), 78.78 (Boc-C), 63.24 (C-1), 58.34 (Val-C-2), 53.51 (Lys-C-2), 50.77 (Leu-C-2), 41.06 (Lys-C-6), 39.90 (Leu-C-3), 31.51 (Lys-C-3), 31.03 (Val-C-3), 29.47 (Lys-C-5), 28.29 (*t*-Bu-CH_3_), 27.88 (Boc-CH_3_), 24.52 (Leu-C-4), 22.57 (Lys-C-4), 22.39 (Val-C-4), 21.79 (Val-C-4), 18.80 (Leu-C-5), 17.43 (Leu-C-5), 17.15 (C-2), −1.73 (Si(CH_3_)_3_). MS (ESI^+^): *m*/*z* = 681.50 [M + Na]^+^. HRMS (ESI^+^): calcd.: 681.4229 [M + Na]^+^, found: 681.4233. IR (ATR): *ν* [cm^−1^] = 3339, 2958, 1731, 1688, 1633, 1546, 1249, 1153, 837. Optical rotation: [α]_D_^25^ = −14.1 (c = 0.91, CHCl_3_). m.p. = 108 °C. TLC: R*_f_* (98:2, CH_2_Cl_2_:MeOH) = 0.48. 

Cbz-protected 6′-*epi* nucleoside building block (**33**): To a solution of the uridine-derived epoxy *tert*-butyl ester **31** (60 mg, 0.10 mmol) in *i*-PrOH (4 mL), *N*-benzyloxycarbonyl-1,3-diaminopropane **32** (31 mg, 0.15 mmol) was added and the mixture was stirred under reflux for 4 d. The solvent was removed under reduced pressure, and the resultant crude product was purified by column chromatography (1:1, petroleum ether-EtOAc) to give **33** as a colourless solid (61 mg, 77%). ^1^H NMR (600 MHz, CDCl_3_): δ [ppm] = 0.05 (s, 3H, SiCH_3_), 0.05 (s, 3H, SiCH_3_), 0.08 (s, 3H, SiCH_3_), 0.10 (s, 3H, SiCH_3_), 0.86 (s, 9H, SiC(CH_3_)_3_), 0.91 (s, 9H, SiC(CH_3_)_3_), 1.47 (s, 9H, OC(CH_3_)_3_), 1.60–1.65 (m, 2H, 2′′-H), 2.39 (ddd, *J* = 11.6, 7.1, 6.9 Hz, 1H, 1′′-H_a_), 2.84 (ddd, *J* = 11.6, 5.8, 5.8 Hz, 1H, 1′′-H_b_), 3.26–3.36 (m, 3H, 6′-H, 3′′-H), 3.84 (dd, *J* = 7.2, 1.1 Hz, 1H, 5′-H), 4.07 (dd, 4.6, 1.1 Hz, 1H, 4′-H), 4.12 (dd, *J* = 4.6, 4.4 Hz, 1H, 3′-H), 4.36 (dd, *J* = 4.5, 4.4 Hz, 1H, 2′-H), 5.03 (dd, *J* = 6.2, 6.1 Hz, 1H, 3′′-NH), 5.08 (d, *J* = 12.2 Hz, 1H, 1′′′-H_a_), 5.10 (d, *J* = 12.2 Hz, 1H, 1′′′-H_b_), 5.52 (d, *J* = 4.5 Hz, 1H, 1′-H), 5.70 (d, *J* = 8.1 Hz, 1H, 5-H), 7.29–7.35 (m, 5H, aryl-H), 7.59 (d, *J* = 8.1 Hz, 1H, 6-H), 8.69 (s, 1H, 3-H). ^13^C NMR (126 MHz, CDCl_3_): δ [ppm] = −4.71 (SiCH_3_), −4.67 (SiCH_3_), −4.62 (SiCH_3_), −4.23 (SiCH_3_), 17.99 (SiC(CH_3_)_3_), 18.11 (SiC(CH_3_)_3_), 25.82 (SiC(CH_3_)_3_), 25.90 (SiC(CH_3_)_3_), 28.13 (OC(CH_3_)_3_), 30.13 (C-2′′), 38.70 (C-3′′), 45.65 (C-1′′), 64.55 (C-6′), 66.67 (C-1′′′), 68.96 (C-5′), 72.18 (C-3′), 73.77 (C-2′), 82.19 (OC(CH_3_)_3_), 84.82 (C-4′), 92.31 (C-1′), 102.03 (C-5), 128.00, 128.08, 128.43 (aryl-C), 136.54 (C-2′′′), 141.81 (C-6), 149.94 (C-2), 156.38 (Cbz-C=O), 162.74 (C-4), 172.04 (C-7′). MS (ESI^+^): *m*/*z* = 793.5 [M + H]^+^. HRMS (ESI^+^): calcd.: 793.4234 [M + H]^+^, found: 793.4234. IR (ATR): *ν* [cm^−1^] = 1682, 1252, 1154, 1121, 867, 834, 813, 776, 735. UV (MeCN): *λ*_max_ (log ε) = 204 (4.24), 261 (3.99). Optical rotation: [α]_D_^25^ = +18.0 (c = 0.55, CHCl_3_). m.p. = 62 °C. TLC: R*_f_* = 0.13 (2:3, petroleum ether:EtOAc).

Cbz-protected 5′-*epi* nucleoside building block (**35**): To a solution of uridine-derived epoxy *tert*-butyl ester **34** (10 mg, 0.017 mmol) in *i*-PrOH (4 mL), *N*-benzyloxycarbonyl-1,3-diaminopropane **32** (5.4 mg, 0.026 mmol) was added and the mixture was stirred under reflux for 3 d. The solvent was removed under reduced pressure, and the resultant crude product was purified by column chromatography (97:3, CH_2_Cl_2_-MeOH) to give **35** as a colourless solid (12 mg, 89%). ^1^H NMR (600 MHz, DMSO-d_6_, 35 °C): δ [ppm] = −0.07 (s, 3H, SiCH_3_), 0.01 (s, 3H, SiCH_3_), 0.08 (s, 3H, SiCH_3_), 0.11 (s, 3H, SiCH_3_), 0.81 (s, 9H, SiC(CH_3_)_3_), 0.89 (s, 9H, SiC(CH_3_)_3_), 1.42 (s, 9H, OC(CH_3_)_3_), 1.49–1.54 (m, 2H, 2′′-H), 2.04 (s, 1H, 6′-NH), 2.34 (ddd, *J* = 10.6, 8.5, 8.2 Hz, 1H, 1′′-H_a_), 2.54 (ddd, *J* = 10.6, 7.1, 6.8 Hz, 1H, 1′′-H_b_), 2.99–3.06 (m, 3H, 6′-H, 3′′-H), 3.70 (ddd, *J* = 6.3, 6.1, 5.4 Hz, 1H, 5′-H), 4.10 (d, *J* = 5.4 Hz, 1H, 4′-H), 4.23 (d, *J* = 4.4 Hz, 1H, 3′-H), 4.32 (dd, *J* = 7.7, 4.4 Hz, 1H, 2′-H), 5.00 (s, 2H, 1′′′-H), 5.58 (d, *J* = 6.3 Hz, 1H, OH), 5.67 (d, *J* = 8.1 Hz, 1H, 5-H), 5.87 (d, *J* = 7.7 Hz, 1H, 1′-H), 7.16 (dd, *J* = 5.9, 5.8 Hz, 1H, 3′′-NH), 7.33–7.36 (m, 5H, aryl-H), 7.73 (d, *J* = 8.1 Hz, 1H, 6-H), 11.31 (s, 1H, 3-H). ^13^C NMR (126 MHz, DMSO-d_6_, 35 °C): δ [ppm] = −5.15 (SiCH_3_), −4.88 (SiCH_3_), −4.63 (SiCH_3_), −4.53 (SiCH_3_), 17.54 (SiC(CH_3_)_3_), 17.67 (SiC(CH_3_)_3_), 25.54 (SiC(CH_3_)_3_), 25.62 (SiC(CH_3_)_3_), 27.76 (OC(CH_3_)_3_), 30.07 (C-2′′), 38.44 (C-3′′), 44.96 (C-1′′), 64.21 (C-6′), 64.97 (C-1′′′), 71.89 (C-3′), 72.22 (C-5′), 73.76 (C-2′), 80.11 (OC(CH_3_)_3_), 85.66 (C-1′), 86.04 (C-4′), 102.09 (C-5), 127.43, 127.47, 128.07 (aryl-C), 137.02 (C-2′′′), 140.51 (C-6), 150.64 (C-2), 155.81 (Cbz-C=O), 162.52 (C-4), 171.66 (C-7′). MS (ESI^+^): *m*/*z* = 793.5 [M + H]^+^. HRMS (ESI^+^): calcd.: 793.4234 [M + H]^+^, found: 793.4239. IR (ATR): *ν* [cm^−1^] = 1689, 1252, 1153, 1057, 833, 813, 775, 735, 697. UV (MeCN): *λ*_max_ (log ε) = 204 (4.13), 259 (3.77). Optical rotation: [α]_D_^25^ = −30.0 (c = 0.24, CHCl_3_). m.p. = 78 °C. TLC: R*_f_* = 0.15 (96:4, CH_2_Cl_2_:MeOH).

### 4.2. Overexpression of MraY from S. aureus

The overexpression of MraY was performed as described before [48]. A plasmid containing the *mraY* gene [48] was transformed into *E. coli* Lemo21 cells, which were plated on lysogeny broth (LB) agar containing kanamycin (50 μg/mL) and chloramphenicol (30 μg/mL). A single colony was picked to induce an overnight culture (10 mL) of LB media containing kanamycin (50 μg/mL) and chloramphenicol (30 μg/mL), which was incubated at 37 °C and 180 rpm for 16 h. A culture of LB media (500 mL) containing kanamycin (50 μg/mL), chloramphenicol (30 μg/mL), and l-rhamnose (1 mM) was inoculated with the overnight culture (500 μL) and then grown at 37 °C and 180 rpm to OD_600_ 0.6. Subsequently, MraY expression was induced with IPTG (1 mM) and the culture was incubated at 37 °C and 180 rpm for 4 h. Cells were then centrifuged and the pellet was resuspended in buffer A (50 mM Tris-HCl buffer pH 7.5, 1 mM MgCl_2_, 2 mM β-mercaptoethanol, 15 mL overall). Egg white lysozyme (spatula tip), DNAse I (spatula tip), and cOmplete^TM^ EDTA-free protease inhibitor cocktail (one tablet) were added. Cells were lysed using sonication and then incubated at 4 °C for 30 min. The lysate was centrifuged and the supernatant was centrifuged again. The resultant pellet was resuspended in buffer A (1.7 mL), flash frozen in liquid nitrogen and stored at −80 °C (aliquots of 20 μL). This MraY-containing crude membrane preparation (overall protein concentration ~20.5 mg/mL as determined by OD_280_) was diluted with water (final overall protein concentration 1 mg/mL) and then directly used for MraY activity assays without further purification.

### 4.3. Fluorescence-Based MraY assay

The assay was performed as described before [40]. The crude membrane preparation of MraY from *S. aureus* (1 μL, vide supra) was added to a mixture of undecaprenyl phosphate (50 μM), dansylated Park′s nucleotide (7.5 μM) [48], and the tested compound (at varying concentrations) in buffer (100 mM Tris-HCl buffer pH 7.5, 200 mM KCl, 10 mM MgCl_2_, 0.1% Triton X-100, 20 μL overall). Fluorescence of the assay mixtures over time was monitored at *λ*_ex_ = 355 nm and *λ*_em_ = 520 nm (plate reader, 384-well plate format). MraY activity at a certain inhibitor concentration was determined using linear regression from 0 to 2 min. This measure of activity was plotted against logarithmic inhibitor concentrations and fitted using a sigmoidal fit. This procedure furnished IC_50_ values with standard deviations.

## 5. Conclusions

In summary, we have demonstrated that the synthesis of 5′-*epi* and 6′-*epi* muraymycin analogues with retained inhibitory activities against the bacterial target enzyme MraY is feasible. The 5′,6′-*anti*-configured 5′-hydroxy motif has been newly established as a versatile structural variation in this context. In particular, (5′*S*,6′*R*)-derivatives (i.e., 5′-hydroxy-6′-*epi* analogues) appear to be promising as their synthesis is highly efficient. Therefore, this novel scaffold will allow the introduction of diverse variations of the 5′-*O*-aminoribose motif for further SAR studies, thus enabling the future optimisation of MraY inhibitor potencies and the development of synthetic muraymycin analogues towards antibacterial drug candidates. Work along this line is on the way in our laboratories.

## Figures and Tables

**Table 1:**
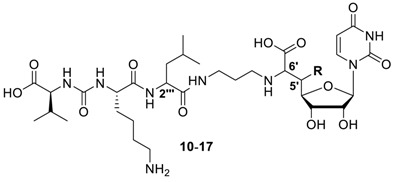
In vitro inhibitory activities of muraymycin analogues **10**–**17** against MraY from *S. aureus*.

Compound	R	Configuration			IC_50_ [μm] ^1^
		5′	6′	2′′′ (Leu)	
**10**	H	-	*S*	*S*	2.5 ± 0.6
**11**	H	-	*S*	*R*	2.0 ± 0.6
**12**	H	-	*R*	*S*	38 ± 6
**13**	H	-	*R*	*R*	24 ± 9
**14**	OH	*S*	*R*	*S*	10 ± 4
**15**	OH	*S*	*R*	*R*	10 ± 2
**16**	OH	*R*	*S*	*S*	6.2 ± 2.8
**17**	OH	*R*	*S*	*R*	21 ± 4
deoxy-C4 **9** ^2^	H	-	*S*	*S*	0.095 ± 0.019

^1^ A crude membrane preparation of MraY from *S. aureus*, heterologously overexpressed in *E. coli*, was used. All measurements were carried out in triplicates. Shown data represent mean values ± standard deviations. ^2^ Structure shown in Figure 1.

## Supplementary Materials

The following are available online, details on the analysis of peptide units using Marfey’s reagent, copies of NMR spectra.
