# Supplementary material for: Analogues of Muraymycin Nucleoside Antibiotics with Epimeric Uridine-Derived Core Structures
Source: Molecules. 2018 Nov 3;23(11):2868. doi: 10.3390/molecules23112868 (PMC6278576; doi:10.3390/molecules23112868)
Supplement: Supplementary file 1 [file molecules-23-02868-s001.pdf]

Supplementary Materials

Analogues of muraymycin nucleoside antibiotics with epimeric uridine-derived core structures

Anatol P. Spork[2,#], Stefan Koppermann[1,#], Stephanie Schier (née Wohnig)[1,2], Ruth Linder[1,2] and Christian Ducho[1,2,*]

[1] Saarland University, Department of Pharmacy, Pharmaceutical and Medicinal Chemistry, Campus C2 3, 66 123 Saarbrücken, Germany

[2] Georg-August-University Göttingen, Department of Chemistry, Institute of Organic and Biomolecular Chemistry, Tammannstr. 2, 37 077 Göttingen, Germany

*Corresponding author *E-mail: christian.ducho@uni-saarland.de*

Table of contents

Analysis of peptide units using Marfey's reagent.. S2

^{1}H, ^{13}C and ^{19}F NMR spectra of synthesized compounds.. S14

References.. S59

Analysis of peptide units using Marfey's reagent

Peptide coupling of uridine-derived building blocks **19-22** with the urea tripeptide **18** and subsequent global deprotection gave two compounds in each of these two-step transformations. Each such pair of compounds showed identical masses and could be separated by preparative HPLC, thus identifying them as diastereomers. We assumed that epimerization in the 2'''-position (*i.e.*, at leucine-C-α) had occurred during the peptide coupling, giving rise to the formation of epimers (cf. Scheme 5 and Table 1). To confirm this assumption and assign the correct stereoconfiguration to each epimer, we used a method based on derivatization with Marfey's reagent **S1** [1], similar to an approach reported by Ichikawa, Matsuda and co-workers [2]. First, we synthesized two reference compounds **S2** and **S3** from L-configured Marfey's reagent **S1** with L-leucine and D-leucine, respectively, in the presence of base (Scheme S1). The L-L-isomer **S2** (obtained from L-leucine) showed a shorter retention time in analytical RP-HPLC relative to the D-L-isomer **S3** (obtained from D-leucine). This behaviour results from the enhanced tendency to form intramolecular H-bond interactions within **S3**, making it less polar than **S2** [1-3].

We then performed an acidic hydrolysis of the isolated muraymycin analogues **10-17** with aqueous HCl (6 M), followed by a transformation of the resultant mixture with Marfey's reagent **S1** in the presence of base (Scheme S1). This procedure should either give **S2** or **S3** as a component of the mixture, depending on the configuration of **10-17** in the 2'''-position (*i.e.*, at leucine-C-α). We therefore analyzed the mixtures by RP-HPLC and additionally performed co-injection experiments. For these, we doped the samples with authentic L-leucine or D-leucine derivatives **S2** or **S3**, respectively, and demonstrated an increased peak area at the respective retention time. According RP-HPLC chromatograms are shown in Figures S1-S19.

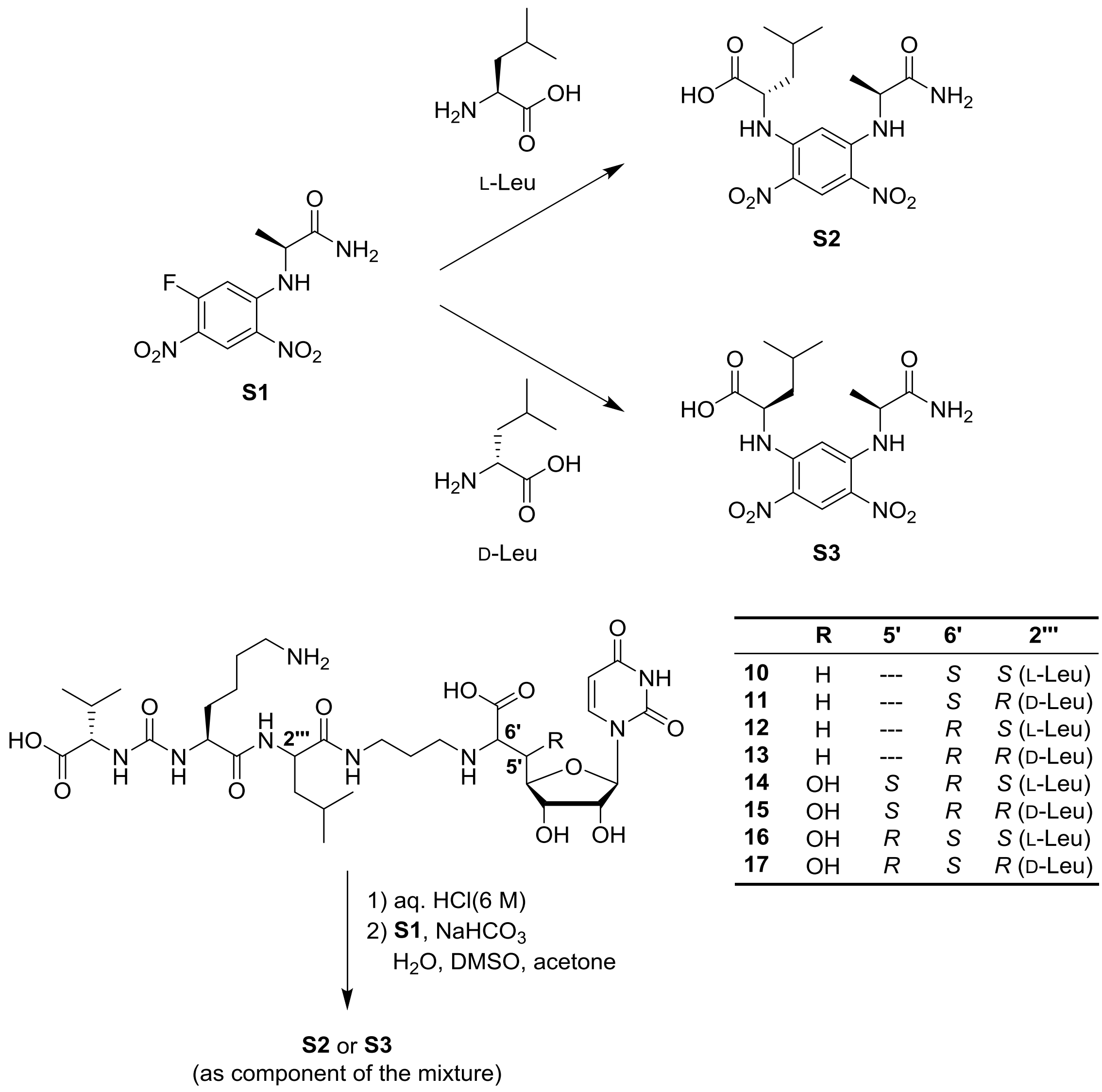

	R	5'	6'	2'''
**10**	H	---	*S*	*S* (L-Leu)
**11**	H	---	*S*	*R* (D-Leu)
**12**	H	---	*R*	*S* (L-Leu)
**13**	H	---	*R*	*R* (D-Leu)
**14**	OH	*S*	*R*	*S* (L-Leu)
**15**	OH	*S*	*R*	*R* (D-Leu)
**16**	OH	*R*	*S*	*S* (L-Leu)
**17**	OH	*R*	*S*	*R* (D-Leu)

Scheme S1. Synthesis of authentic standards **S2** and **S3** and reaction of Marfey's reagent with hydrolyzed muraymycin analogues **10-17** for stereochemical analysis of the peptide units.

Method for analytical HPLC. eluent A water, eluent B MeOH; 0-15 min gradient of B (80-90%), 15-25 min 100% B, 25-30 min 80% B; flow 1 mL/min.

Synthesis of standards S2 and S3. In two separate reactions, to a solution of either L-leucine or D-leucine (25 mM in water, 80 µL, 2.0 µmol) in DMSO (15 µL), Marfey's reagent **S1** (20 mM in acetone, 50 µL, 1.0 µmol) and NaHCO$_3$ (1 M in water, 5 µL, 5 µmol) were added

and the reaction mixture was stirred at 40 °C for 15 h. Then, HCl (1 M, 10 µL, 10 µmol) was added. The solvent was removed by lyophilization, and the resultant residue was dissolved in DMSO (1 mL) and analyzed by RP-HPLC (t_R = 34.6 min (**S2**), t_R = 42.3 min (**S3**)). The eluents of the analytical HPLC runs were collected and analyzed by MS to confirm the identity of the respective compounds. **S2:** MS (ESI^+): m/z = 406.2 $[M+Na]^+$. HRMS (ESI^+): calcd.: 406.1333 $[M+Na]^+$, found: 406.1316. **S3:** MS (ESI^+): m/z = 406.2 $[M+Na]^+$. HRMS (ESI^+): calcd.: 406.1333 $[M+Na]^+$, found: 406.1330.

Analysis of the peptide units in 10-17. A solution of the respective muraymycin analogue **10-17** (0.1 mg, 0.1 µmol) in HCl (6 M, 200 µL) was heated to 120 °C for 20 h. The solvent was then removed under reduced pressure, and the resultant residue was dissolved in water (80 µL) and DMSO (15 µL). Marfey's reagent **S1** (20 mM in acetone, 50 µL, 1.0 µmol) and $NaHCO_3$ (1 M in water, 5 µL, 5 µmol) were added and the reaction mixture was stirred at 40 °C for 16 h. Then, HCl (1 M, 10 µL, 10 µmol) was added. The solvent was removed by lyophilization, and the resultant residue was dissolved in DMSO (1 mL) and analyzed by RP-HPLC. The according RP-HPLC chromatograms are shown in Figures S1-S19.

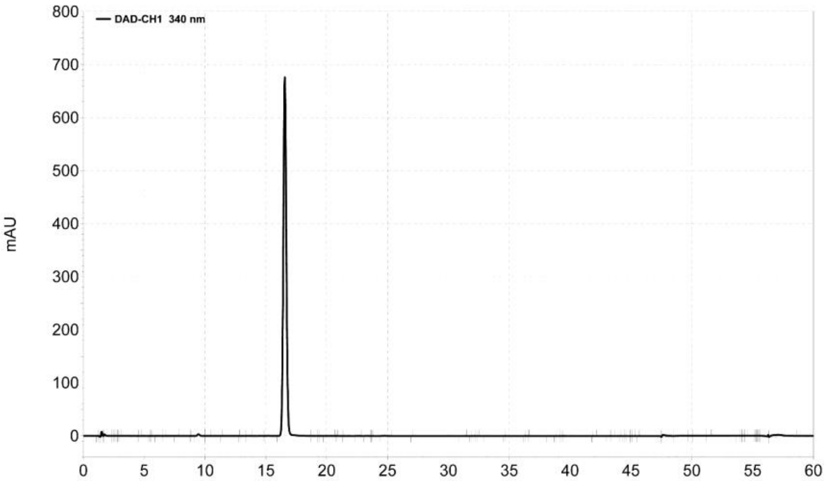

Figure S1. HPLC chromatogram of Marfey's reagent **S1**.

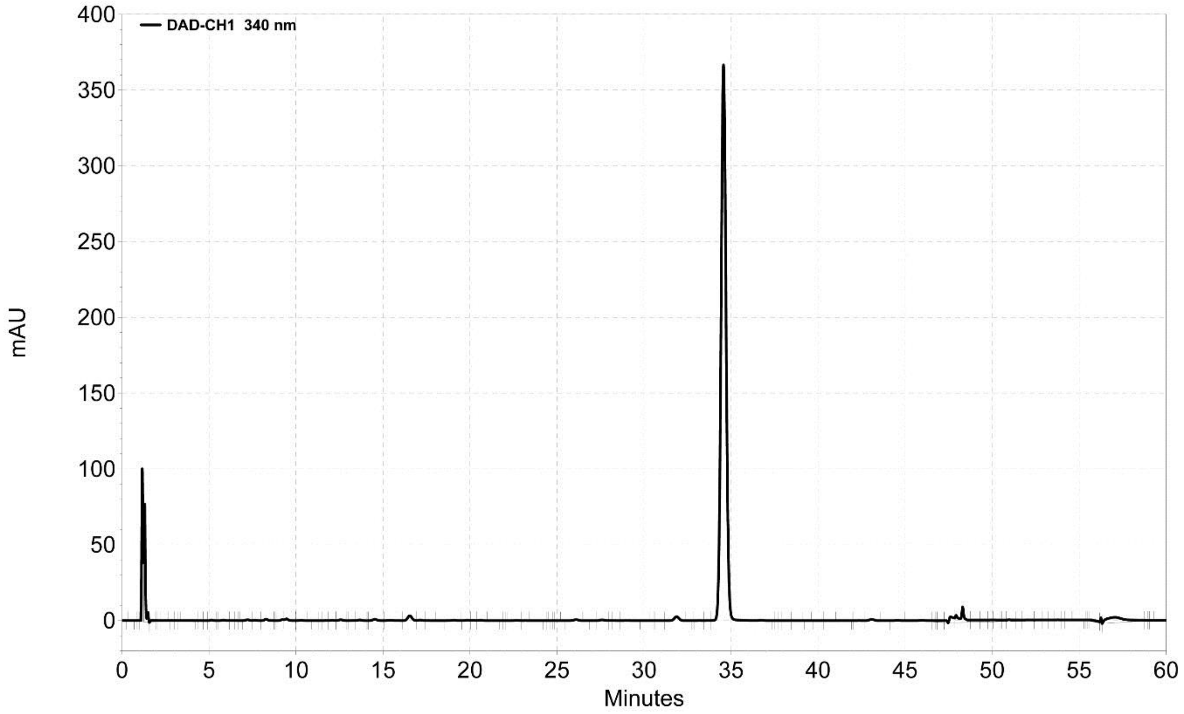

Figure S2. HPLC chromatogram of the L-L-configured authentic standard **S2**.

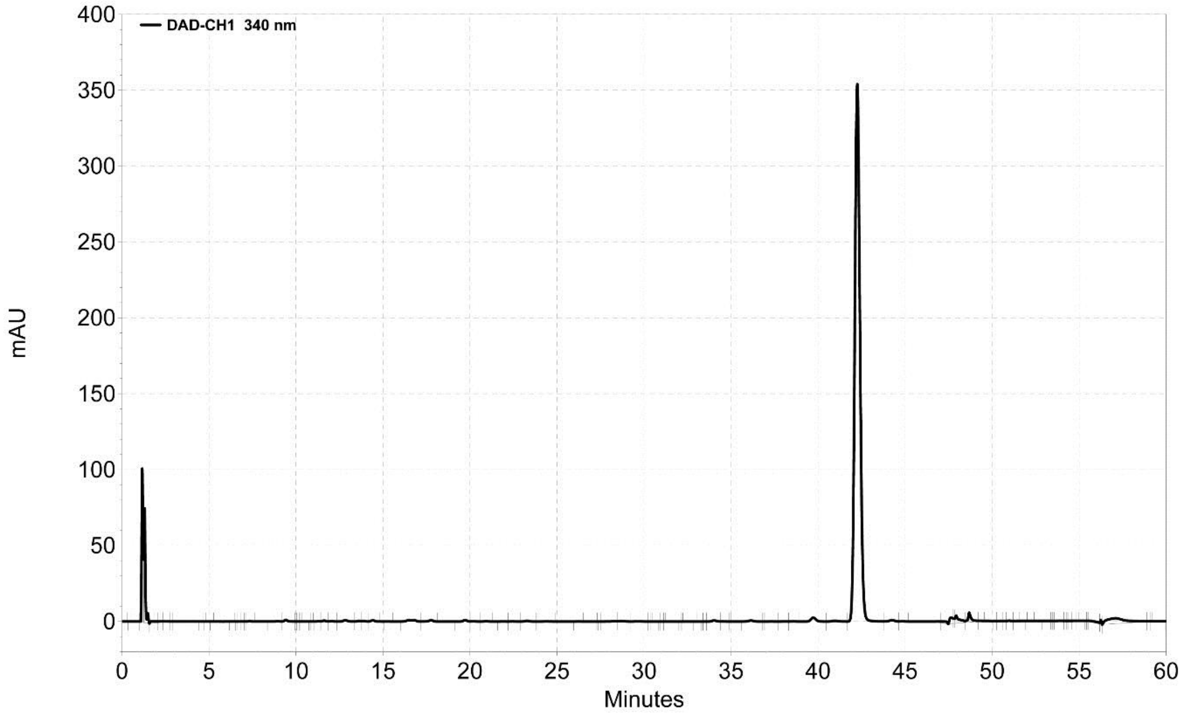

Figure S3. HPLC chromatogram of the D-L-configured authentic standard **S3**.

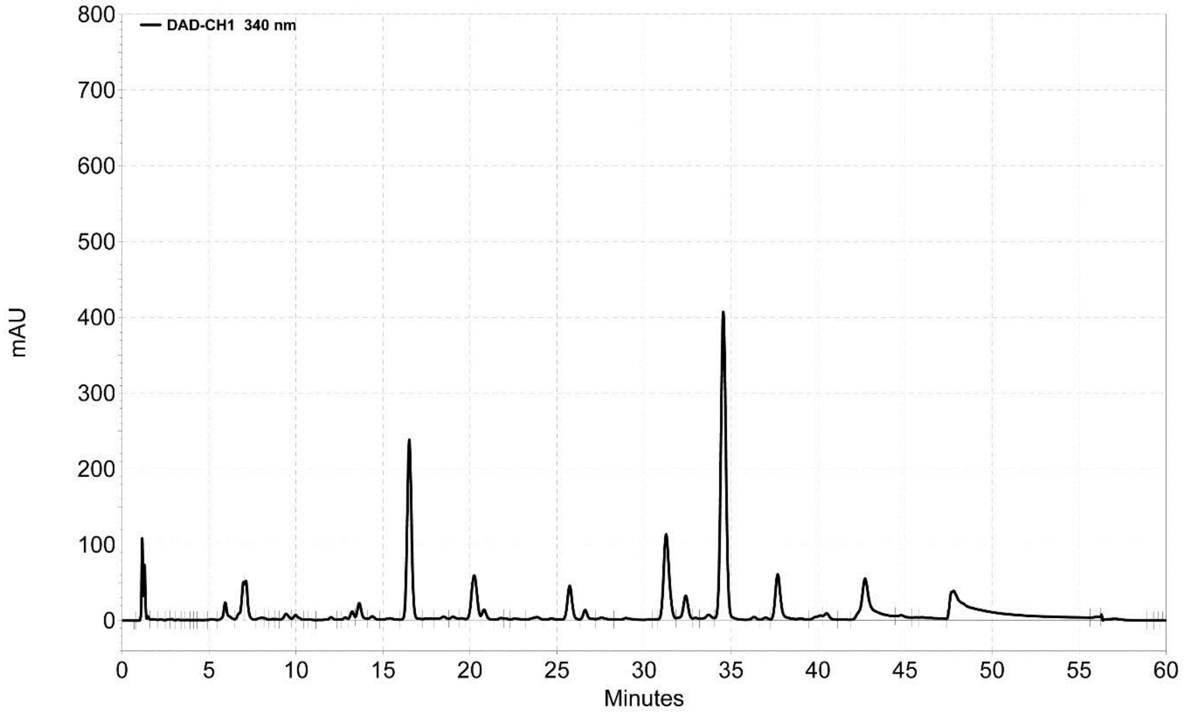

Figure S4. HPLC chromatogram of the mixture obtained from **10**.

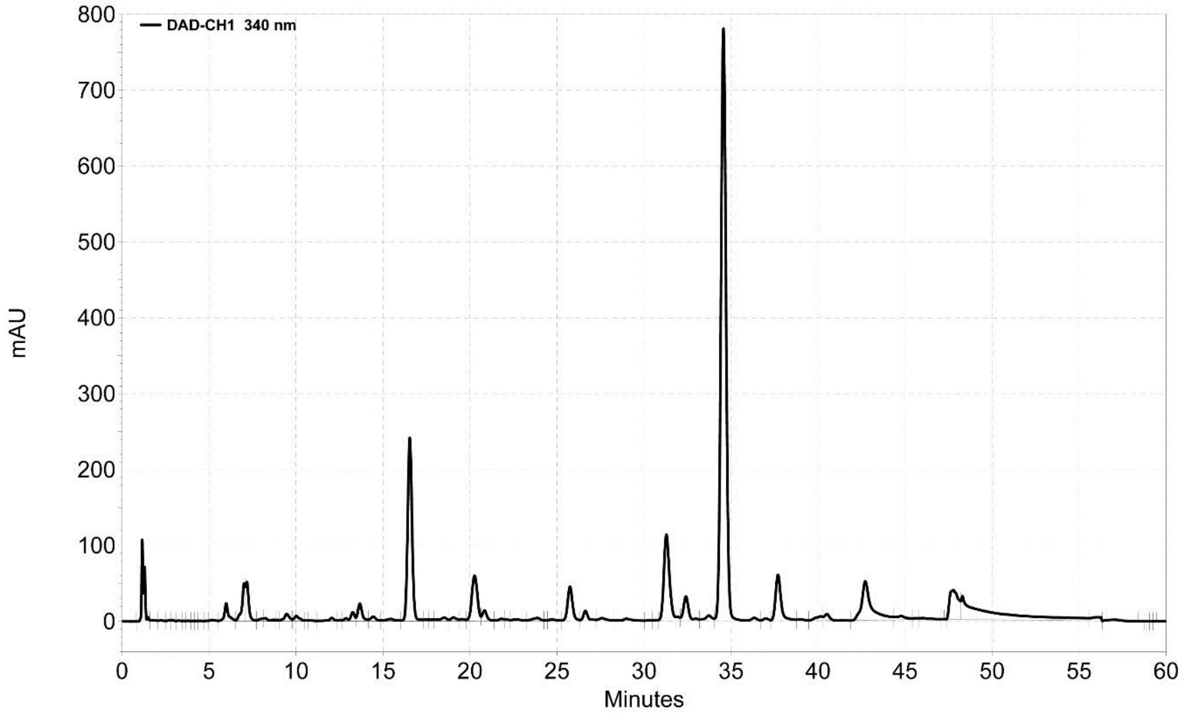

Figure S5. Co-injection of the mixture obtained from **10** with standard **S2**.

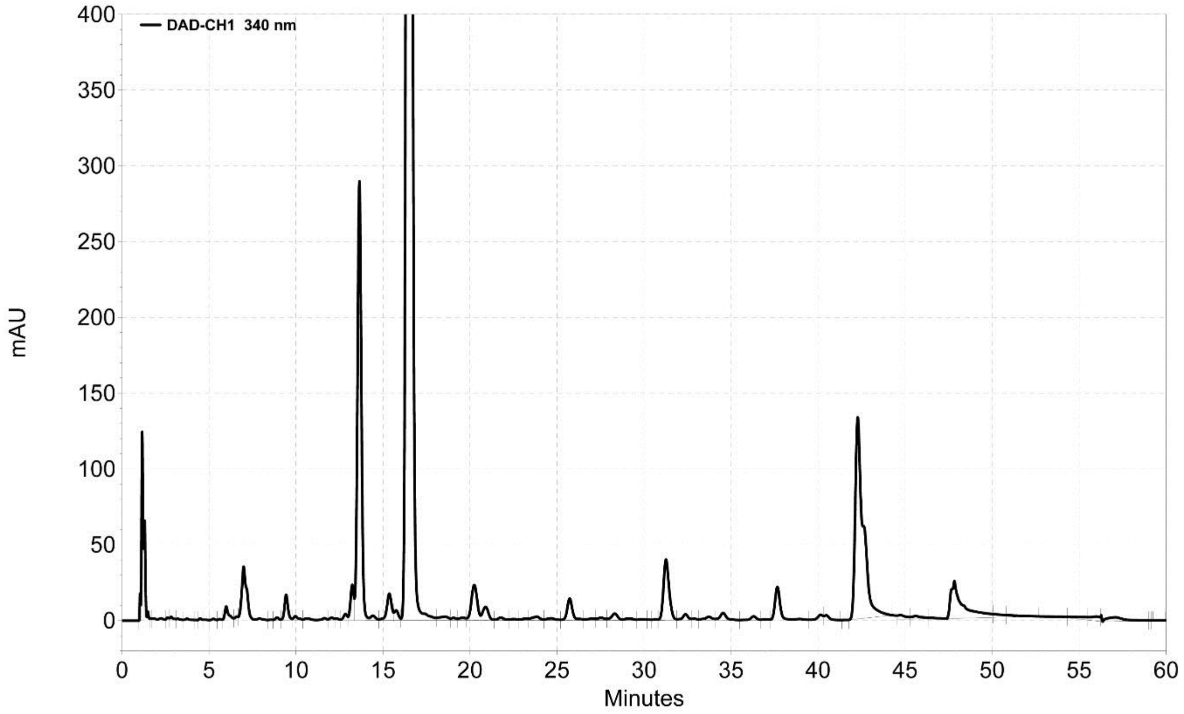

Figure S6. HPLC chromatogram of the mixture obtained from **11**.

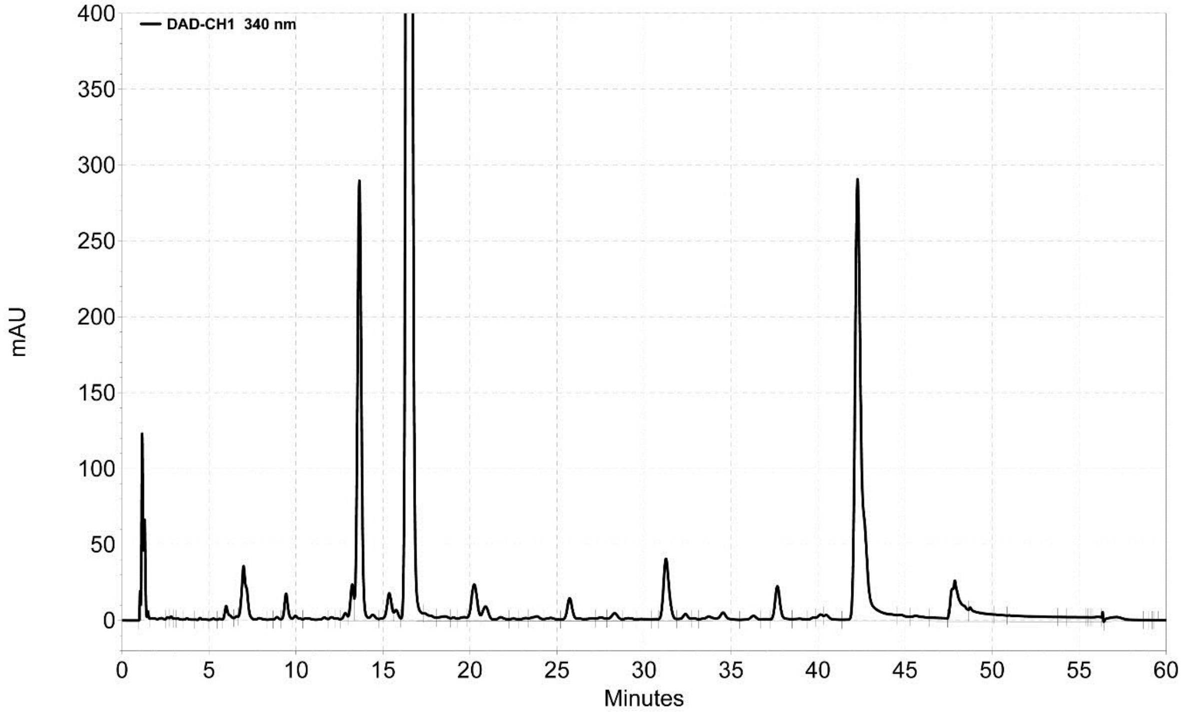

Figure S7. Co-injection of the mixture obtained from **11** with standard **S3**.

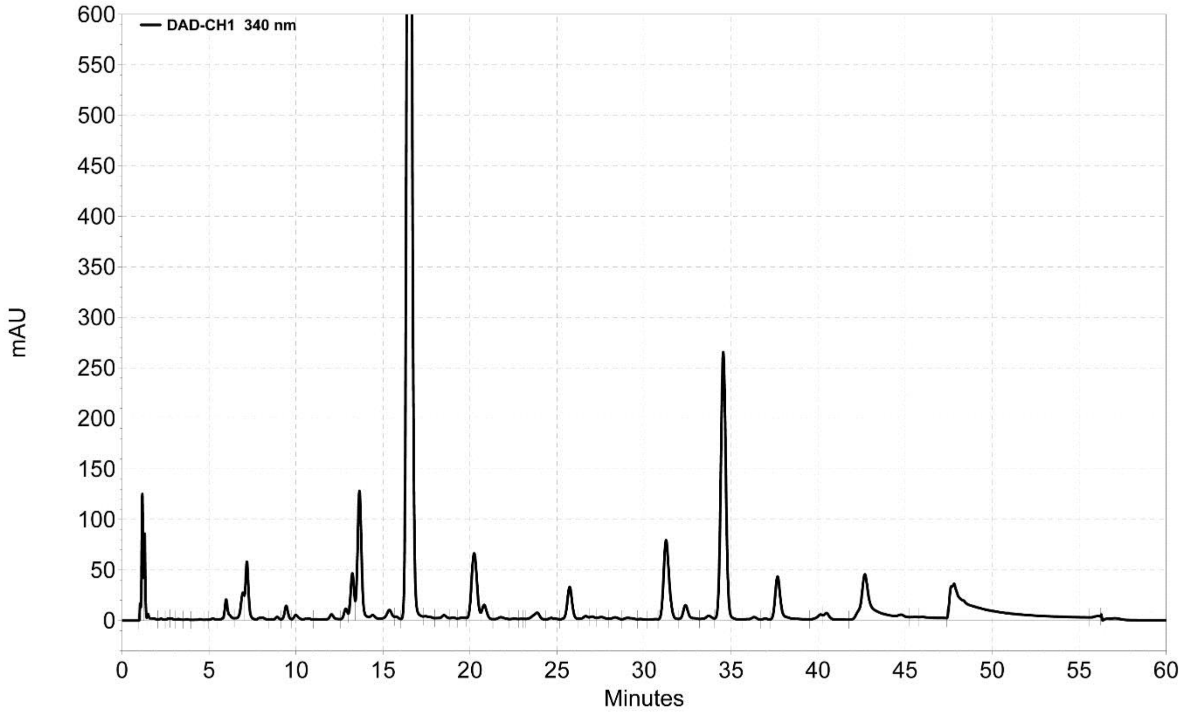

Figure S8. HPLC chromatogram of the mixture obtained from **12**.

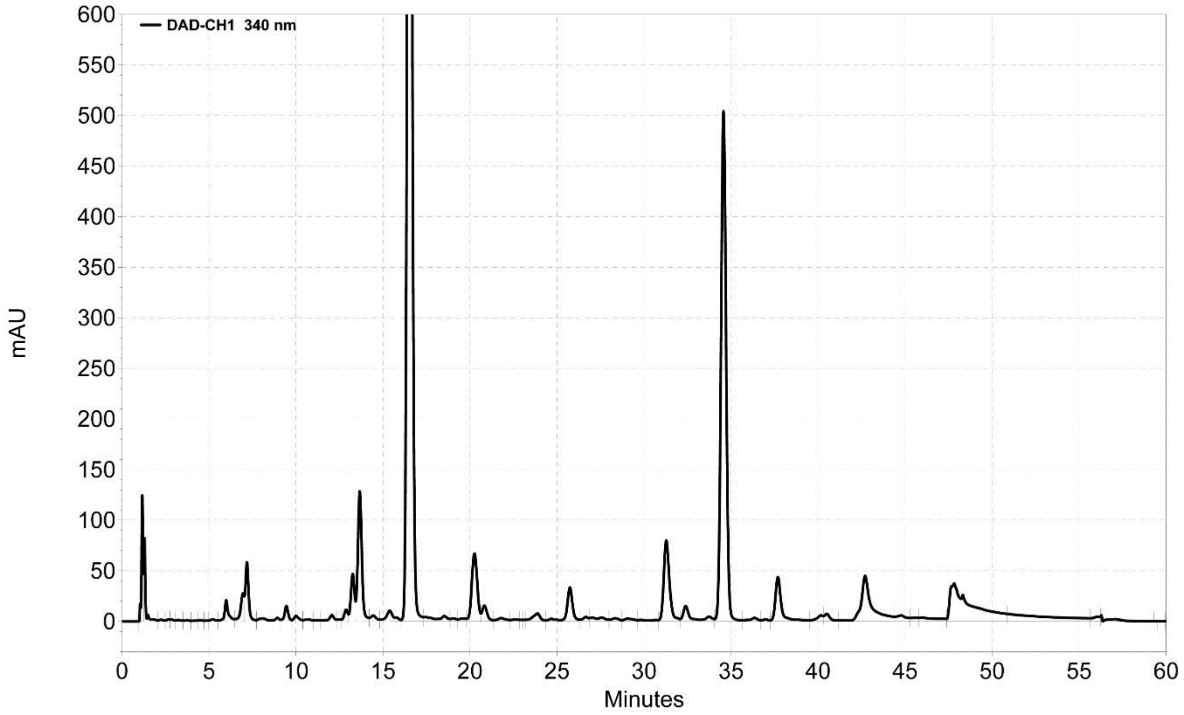

Figure S9. Co-injection of the mixture obtained from **12** with standard **S2**.

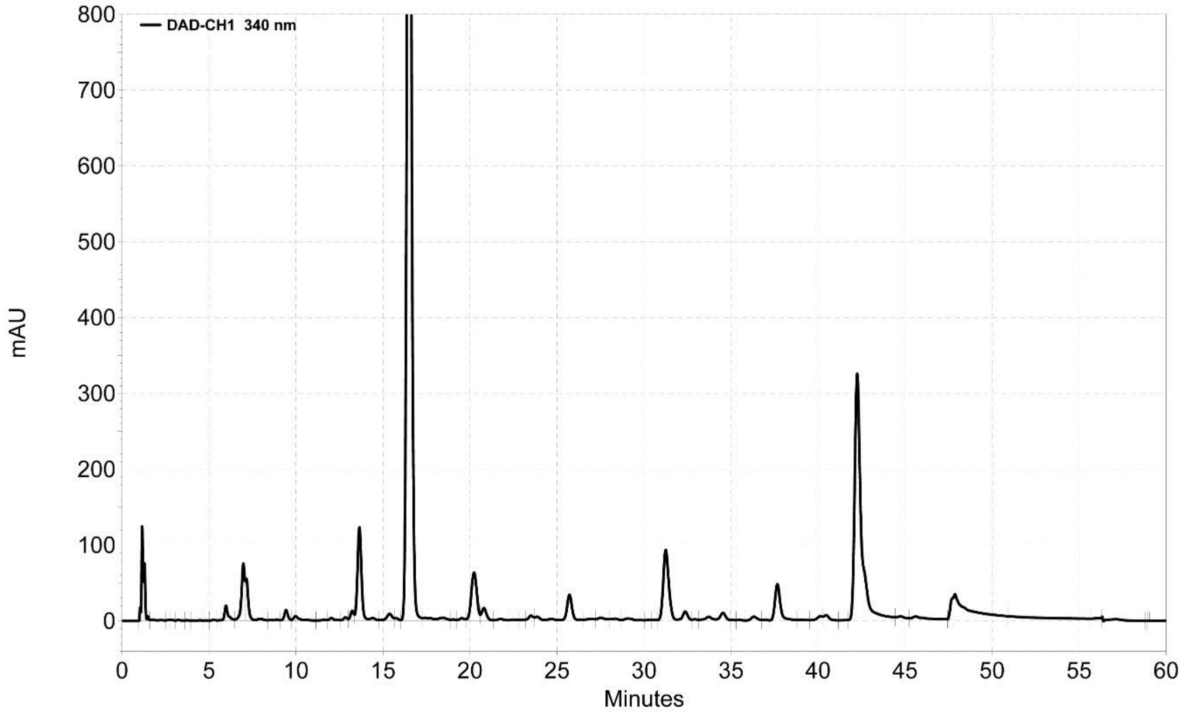

Figure S10. HPLC chromatogram of the mixture obtained from **13**.

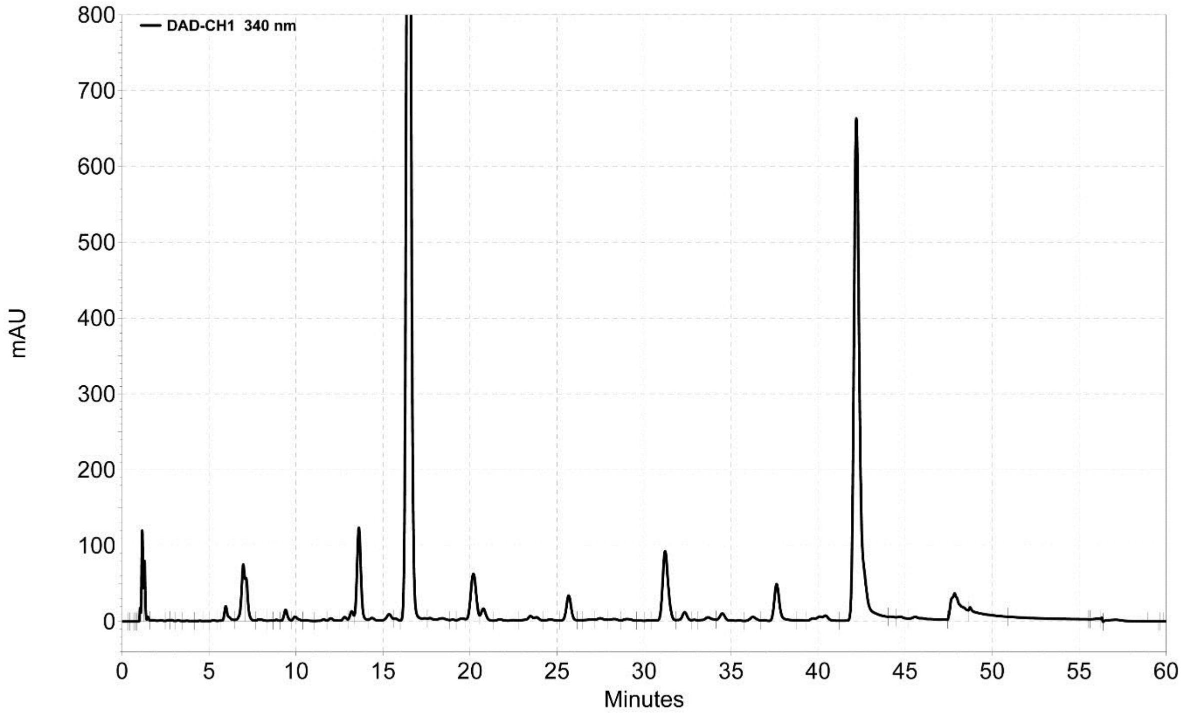

Figure S11. Co-injection of the mixture obtained from **13** with standard **S3**.

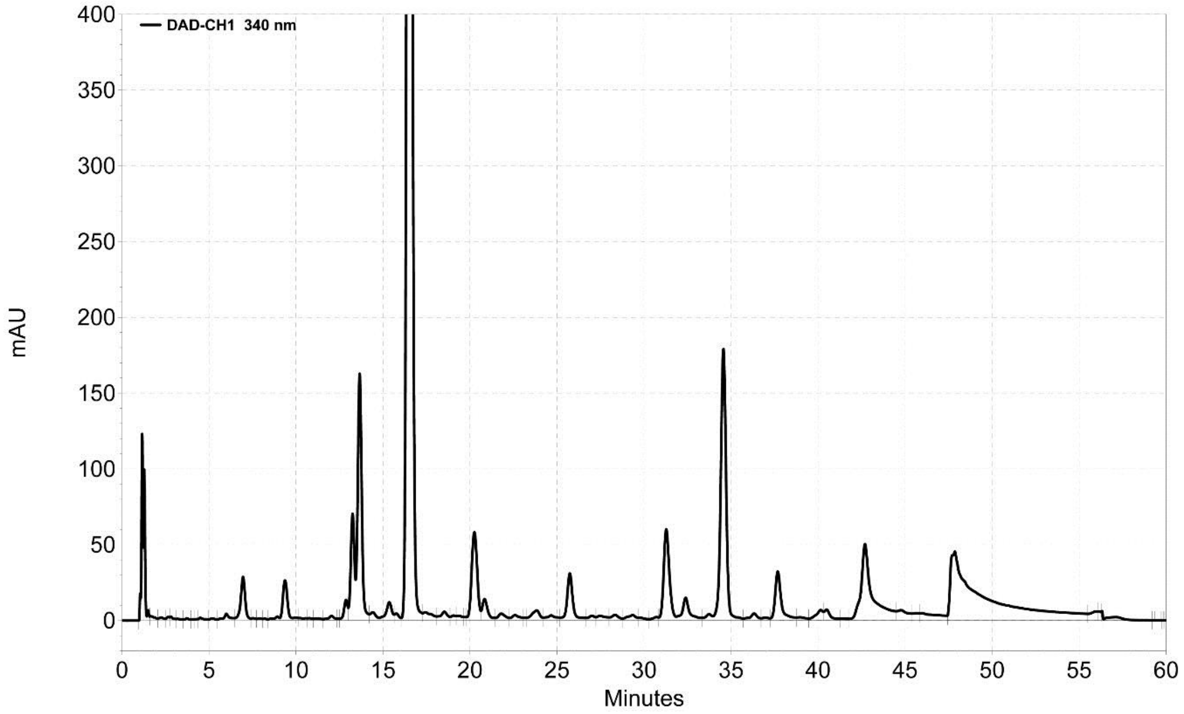

Figure S12. HPLC chromatogram of the mixture obtained from **14**.

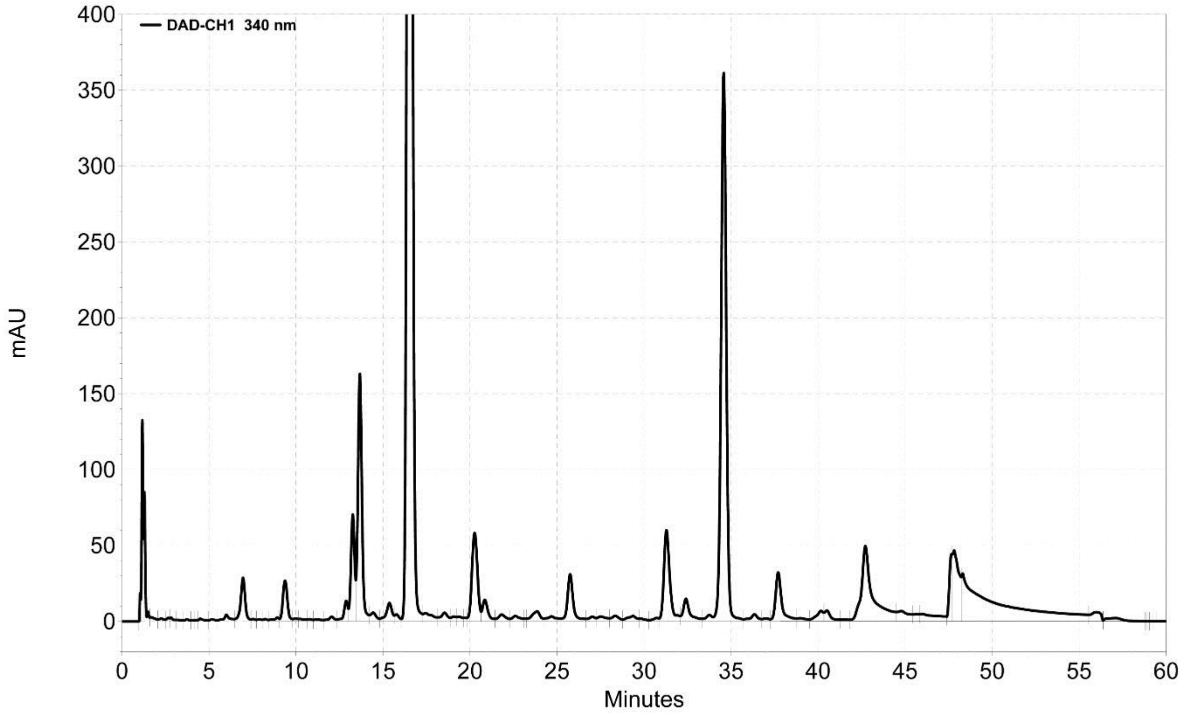

Figure S13. Co-injection of the mixture obtained from **14** with standard **S2**.

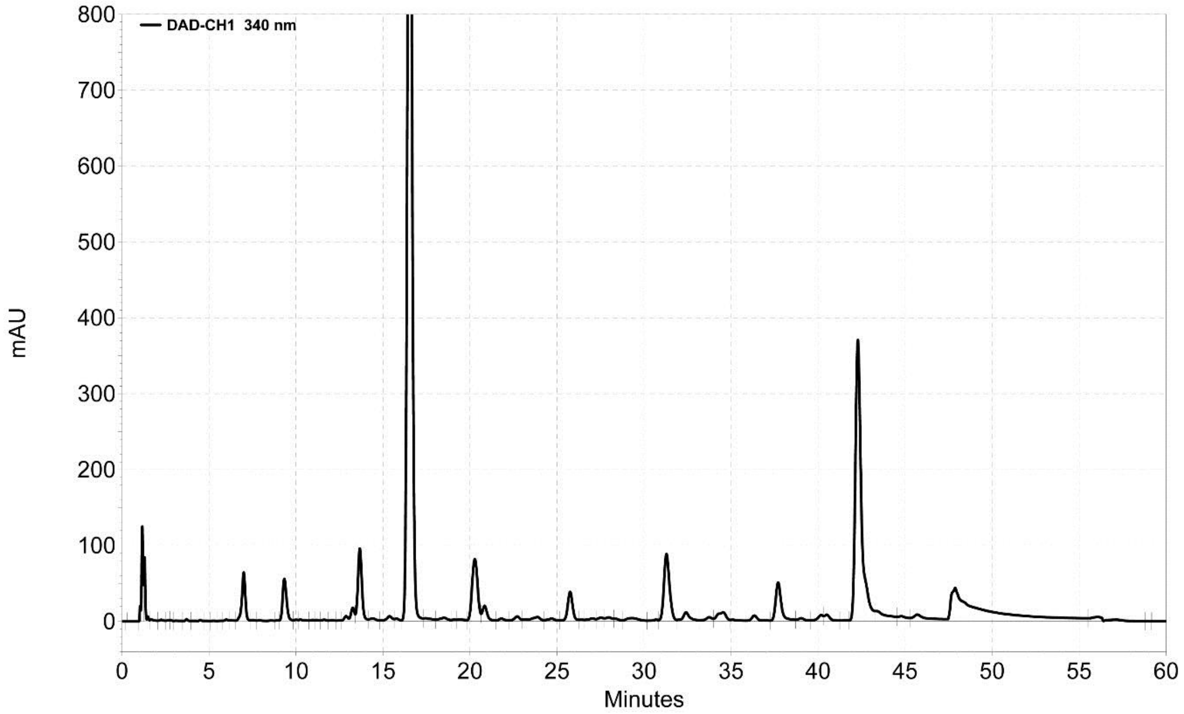

Figure S14. HPLC chromatogram of the mixture obtained from **15**.

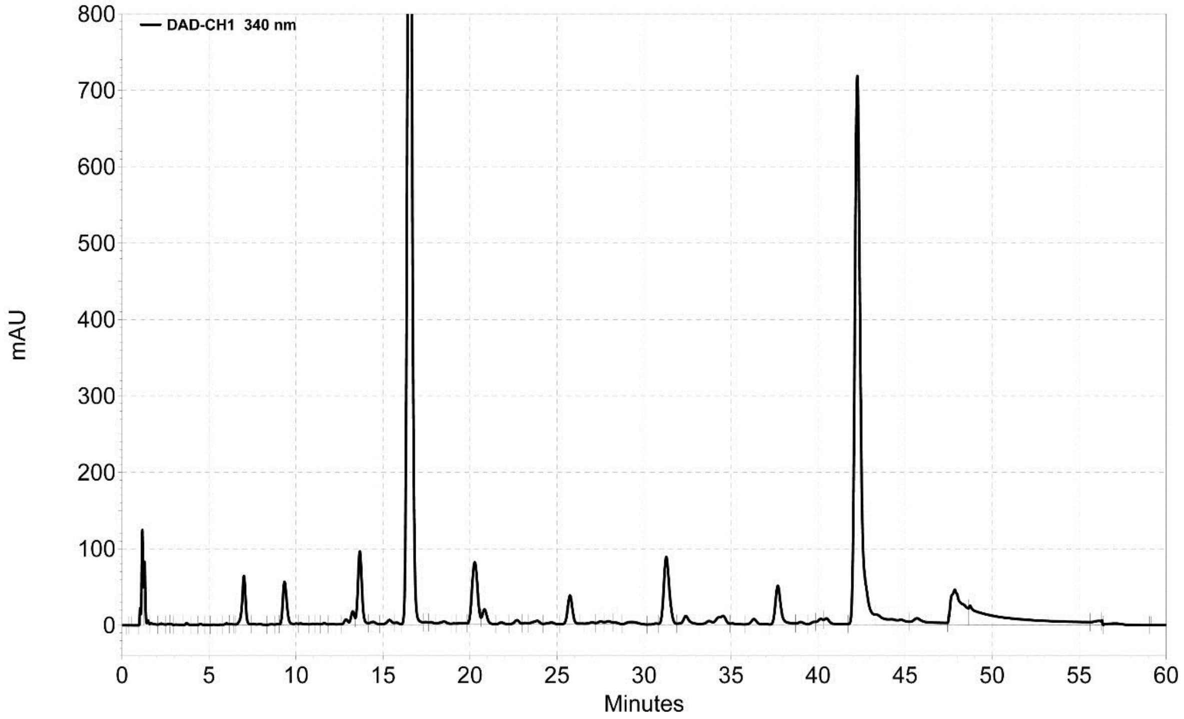

Figure S15. Co-injection of the mixture obtained from **15** with **S3**.

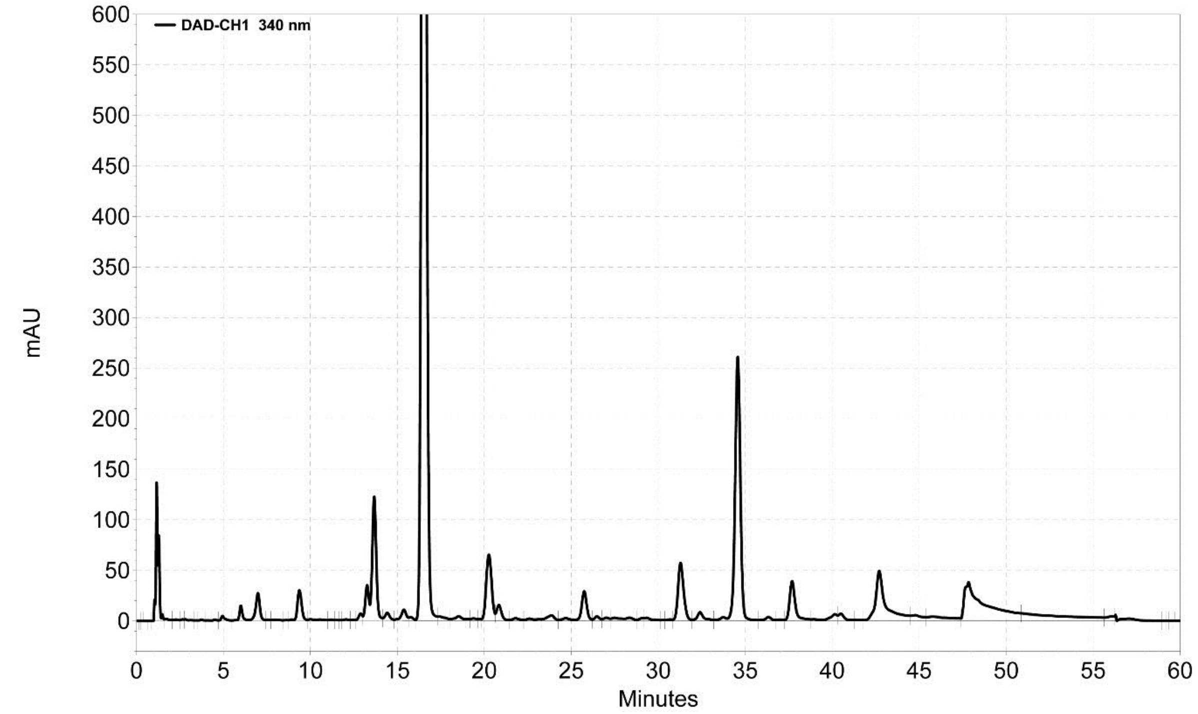

Figure S16. HPLC chromatogram of the mixture obtained from **16**.

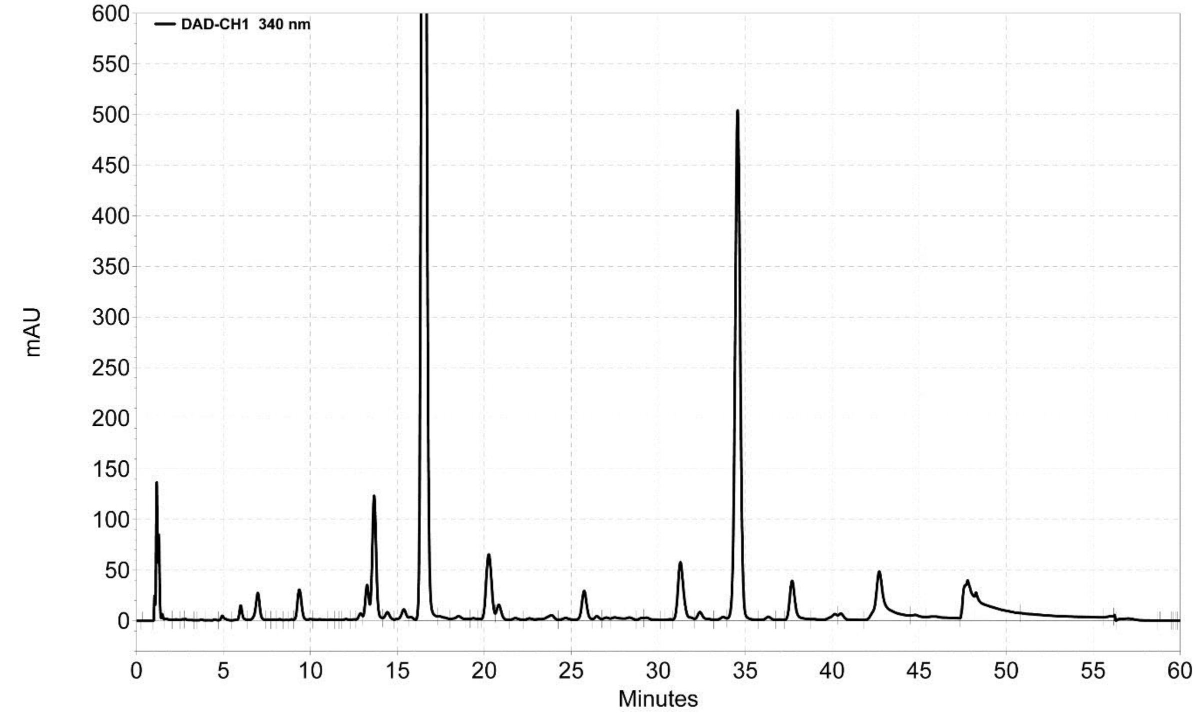

Figure S17. Co-injection of the mixture obtained from **16** with standard **S2**.

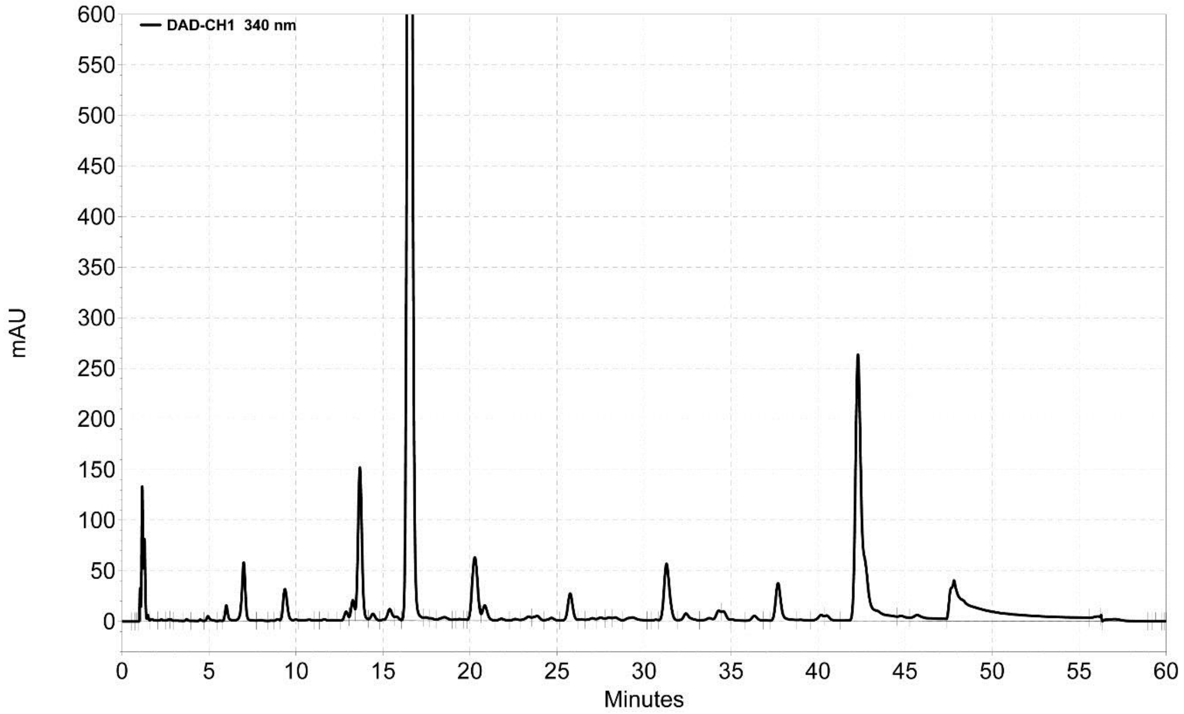

Figure S18. HPLC chromatogram of the mixture obtained from **17**.

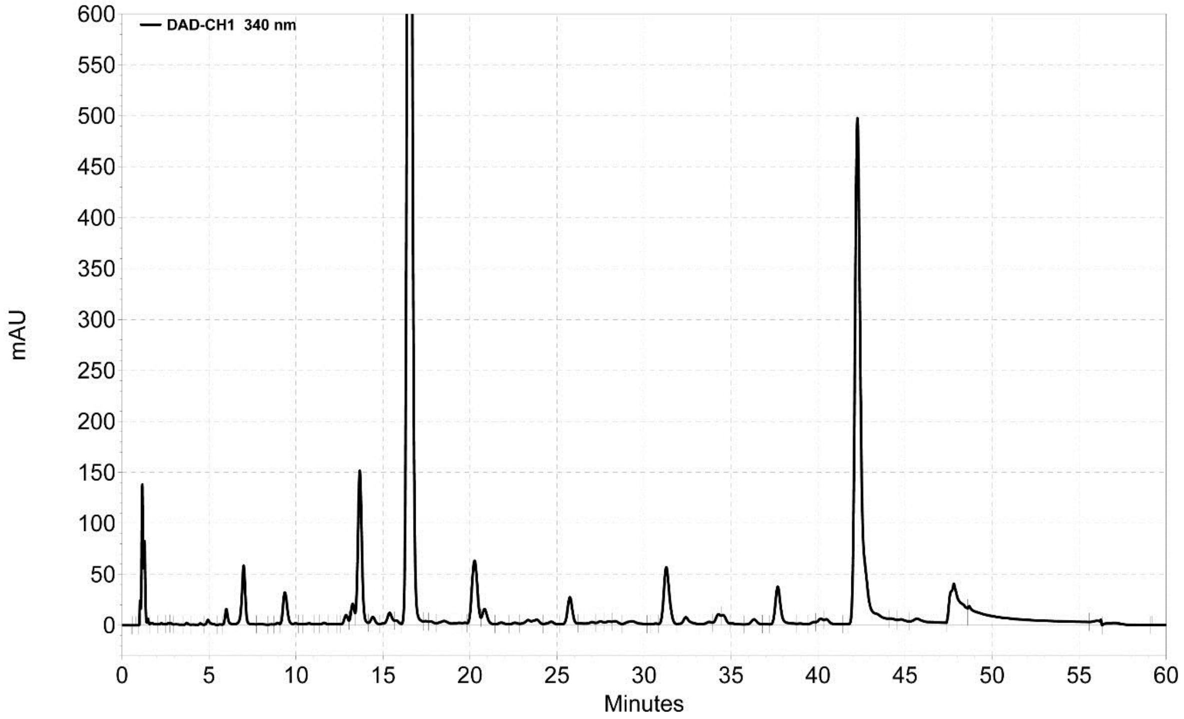

Figure S19. Co-injection of the mixture obtained from **17** with standard **S3**.

^{1}H, ^{13}C and ^{19}F NMR spectra of synthesized compounds

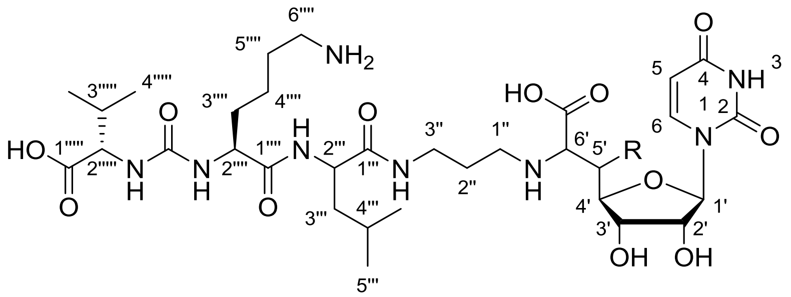

Figure S20. Numbering of atoms of muraymycin target structures for the assignment of NMR signals.

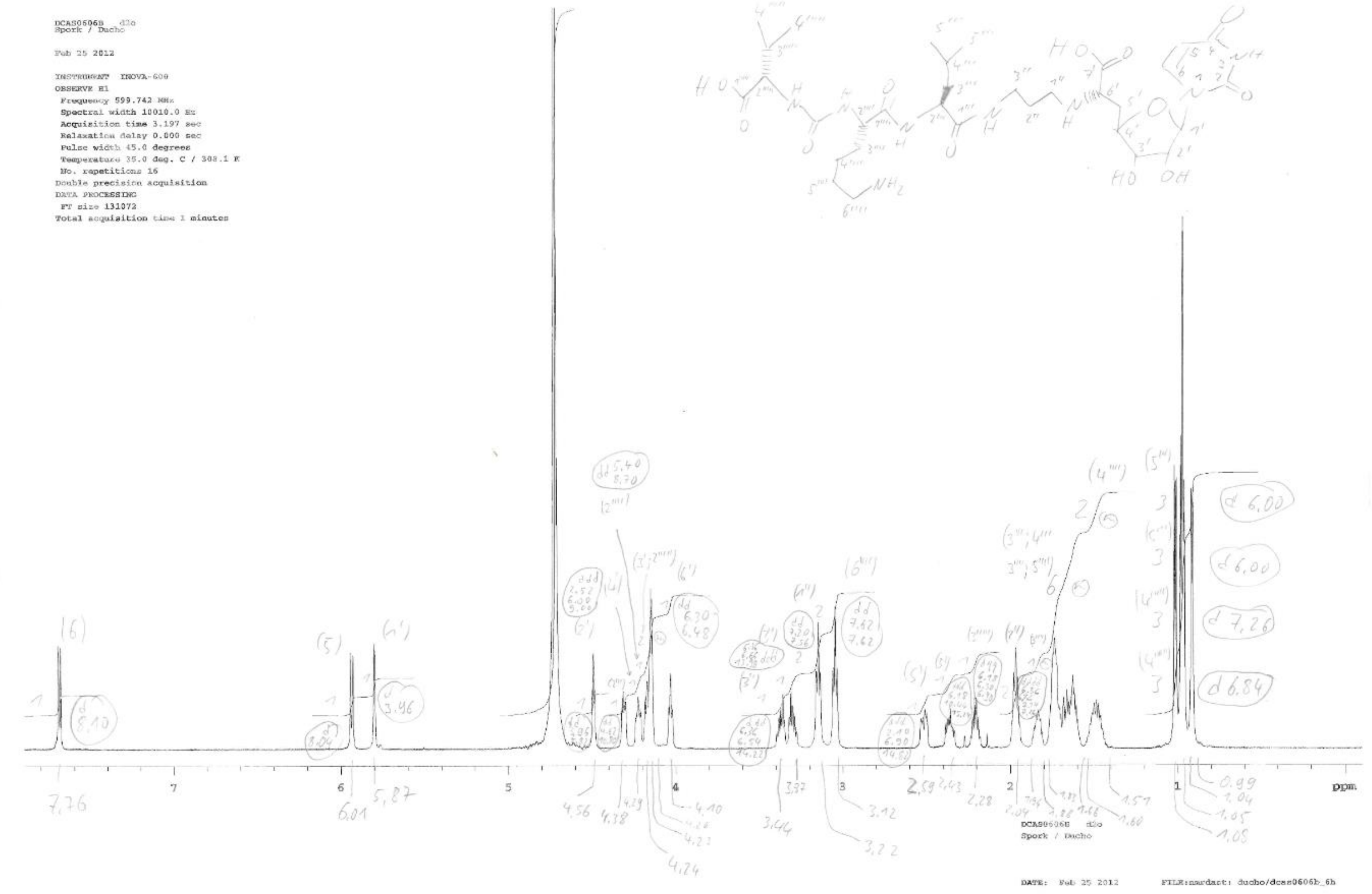

^{1}H NMR spectrum of **10** (600 MHz, D_2O, 35 °C)

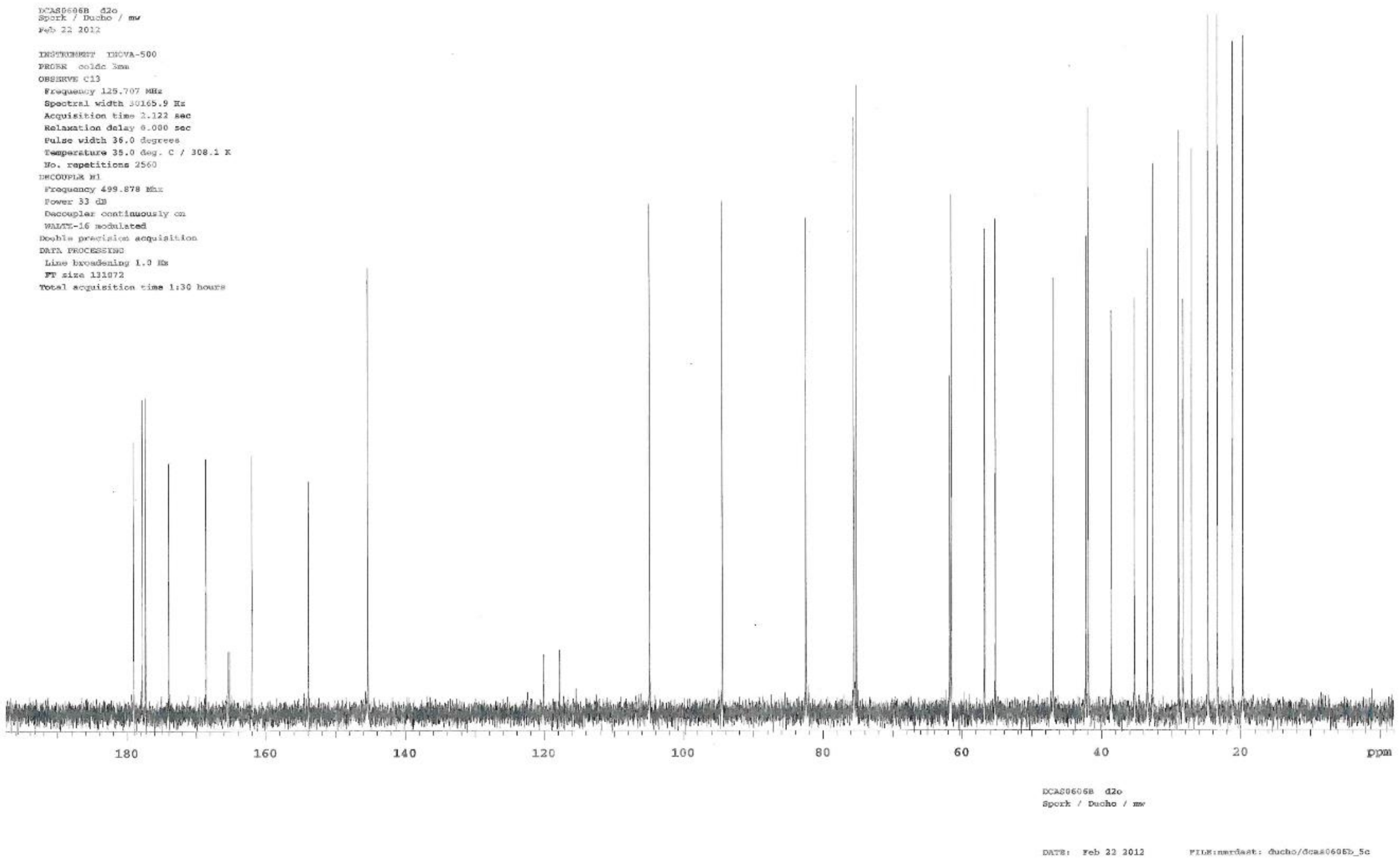

^{13}C NMR spectrum of **10** (126 MHz, D_2O, 35 °C)

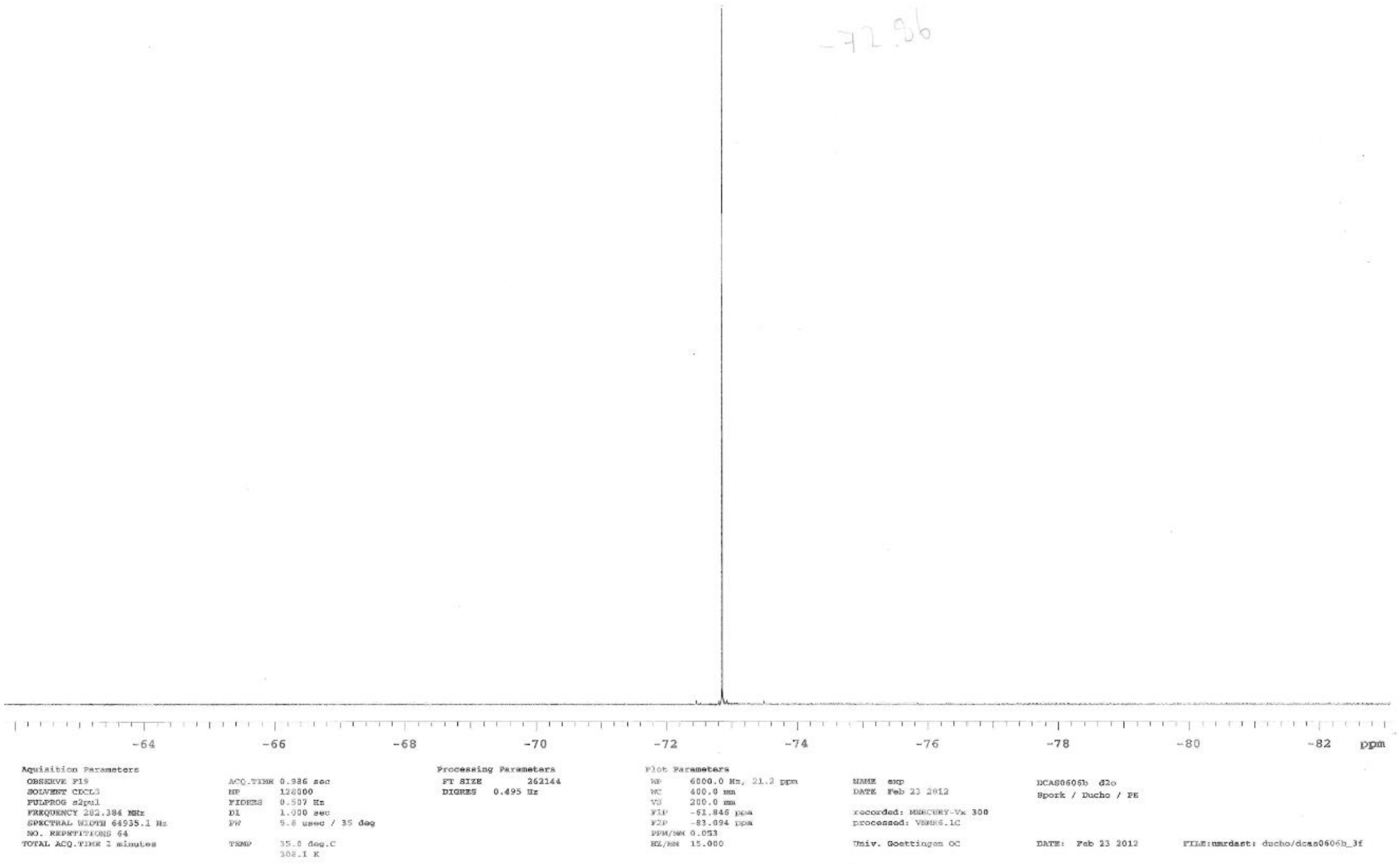

^{19}F NMR spectrum of **10** (282 MHz, D_2O, 35 °C)

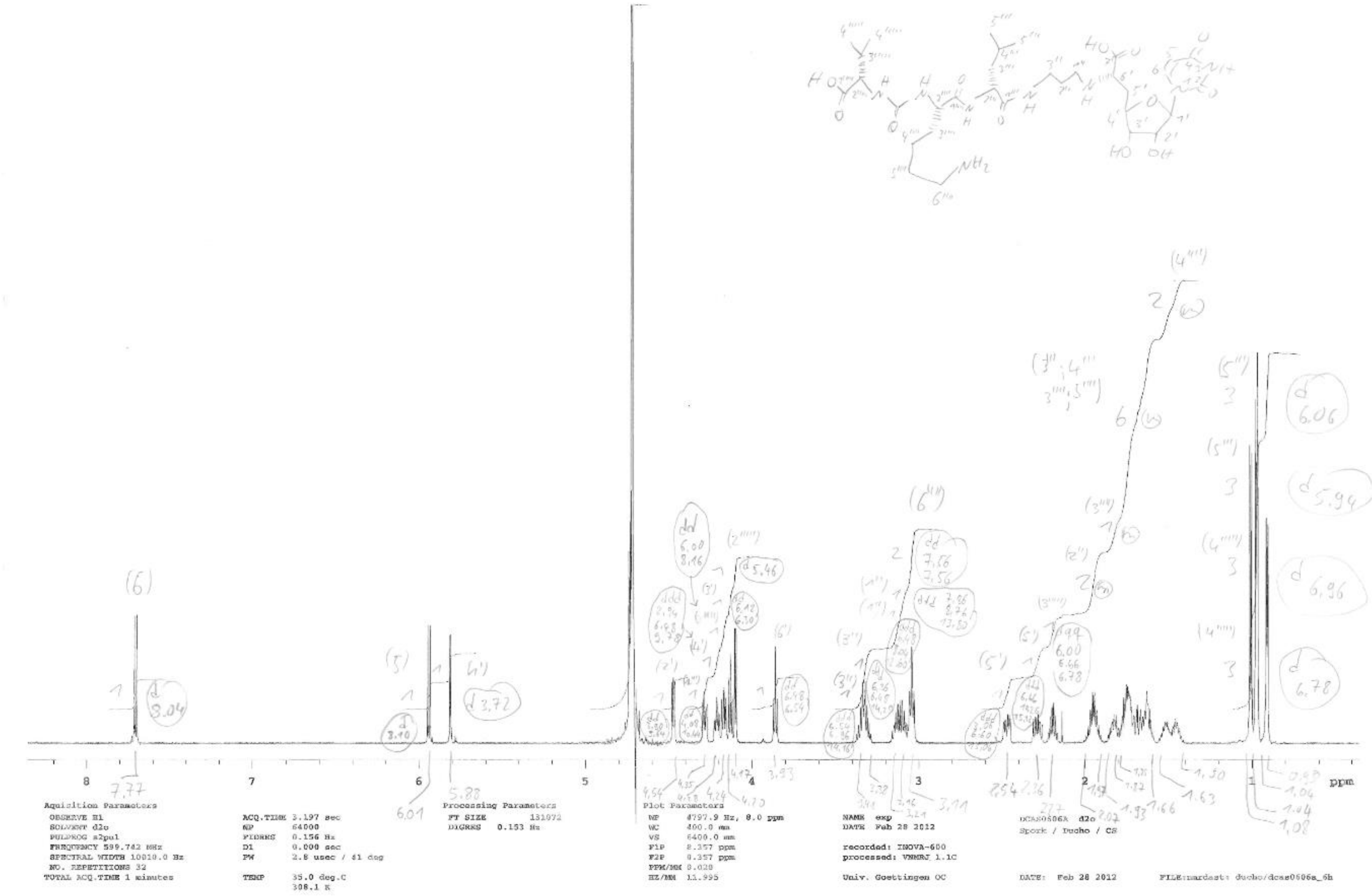

^{1}H NMR spectrum of **11** (600 MHz, D_2O, 35 °C)

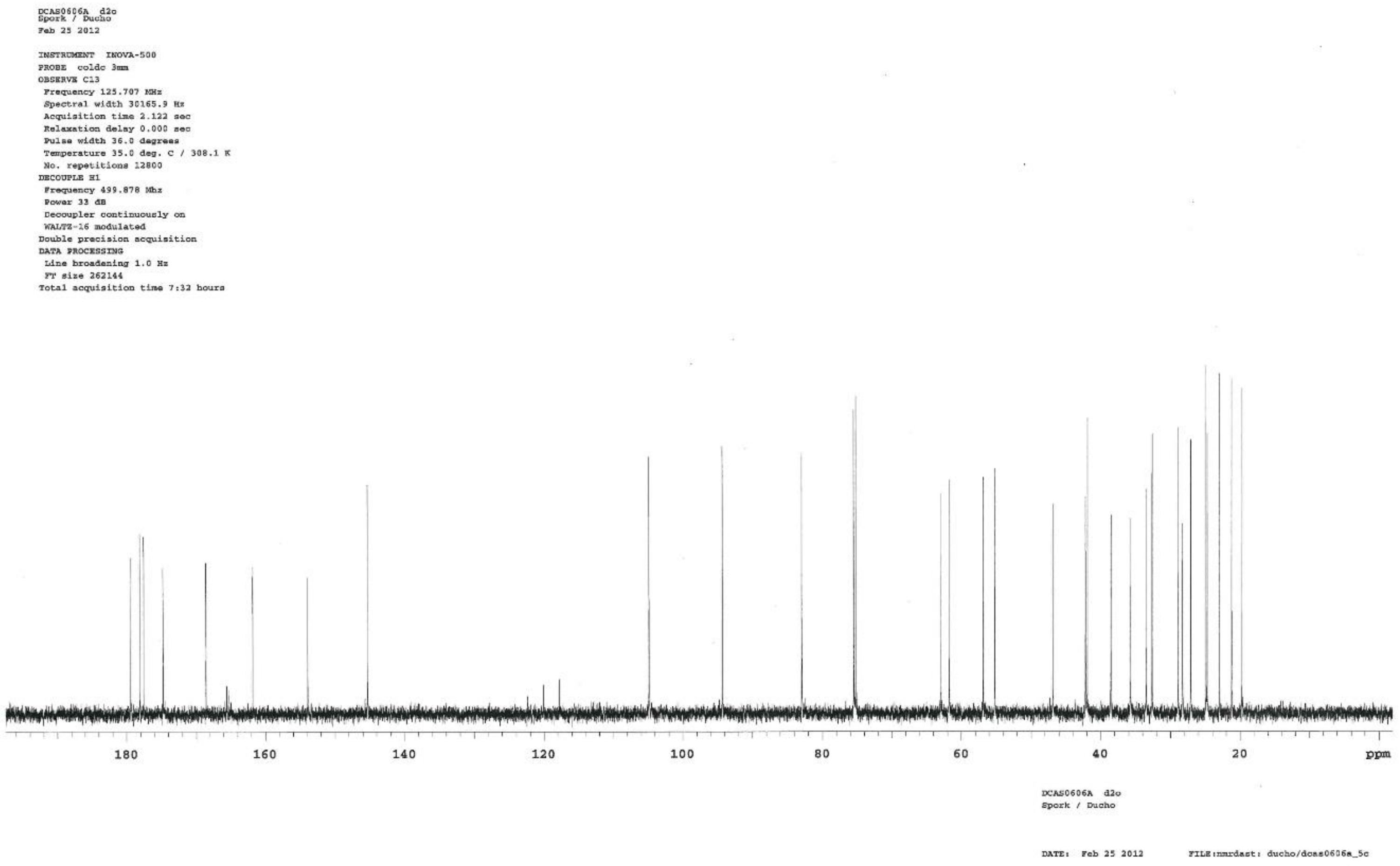

^{13}C NMR spectrum of **11** (126 MHz, D_2O, 35 °C)

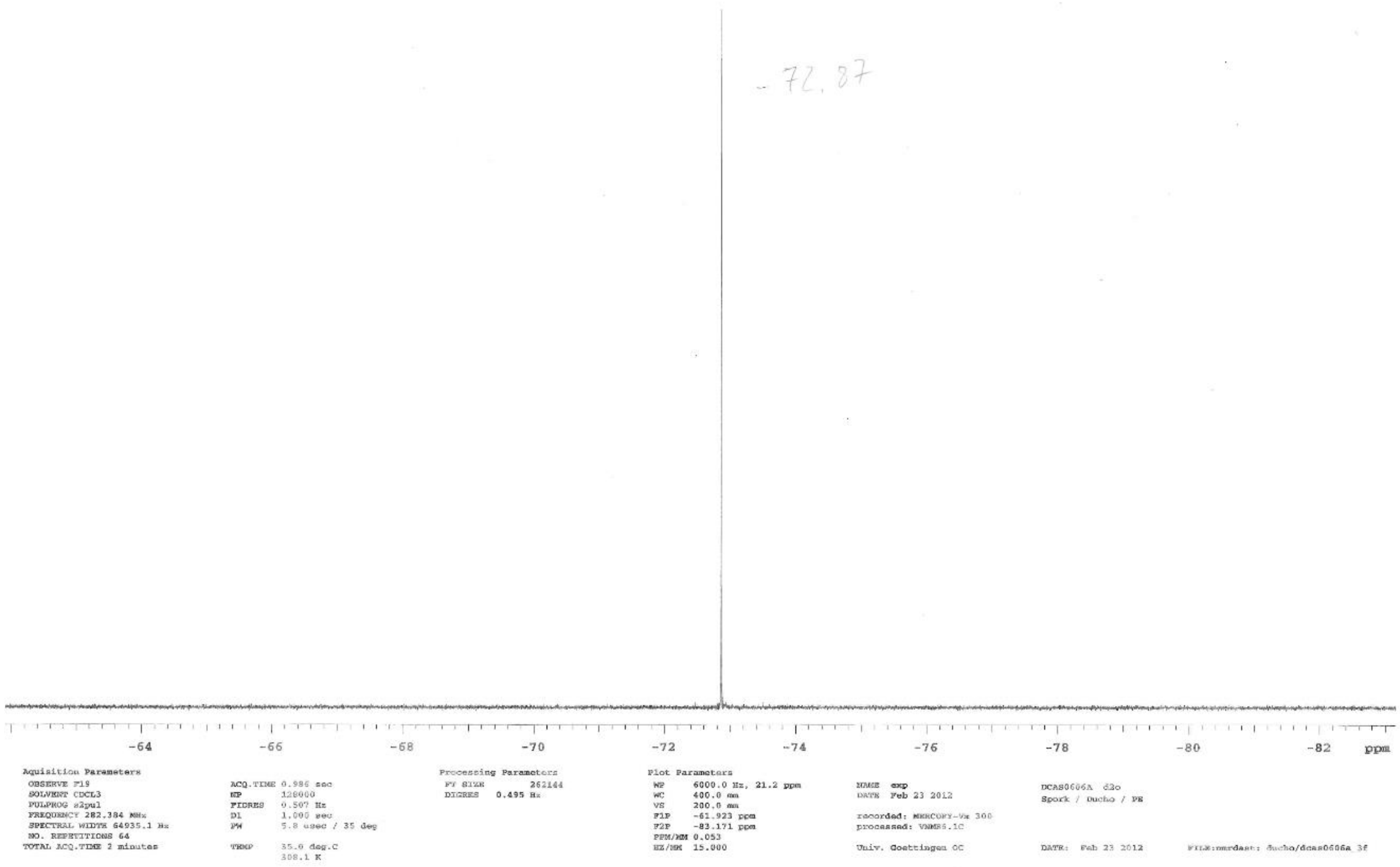

^{19}F NMR spectrum of **11** (282 MHz, D_2O, 35 °C)

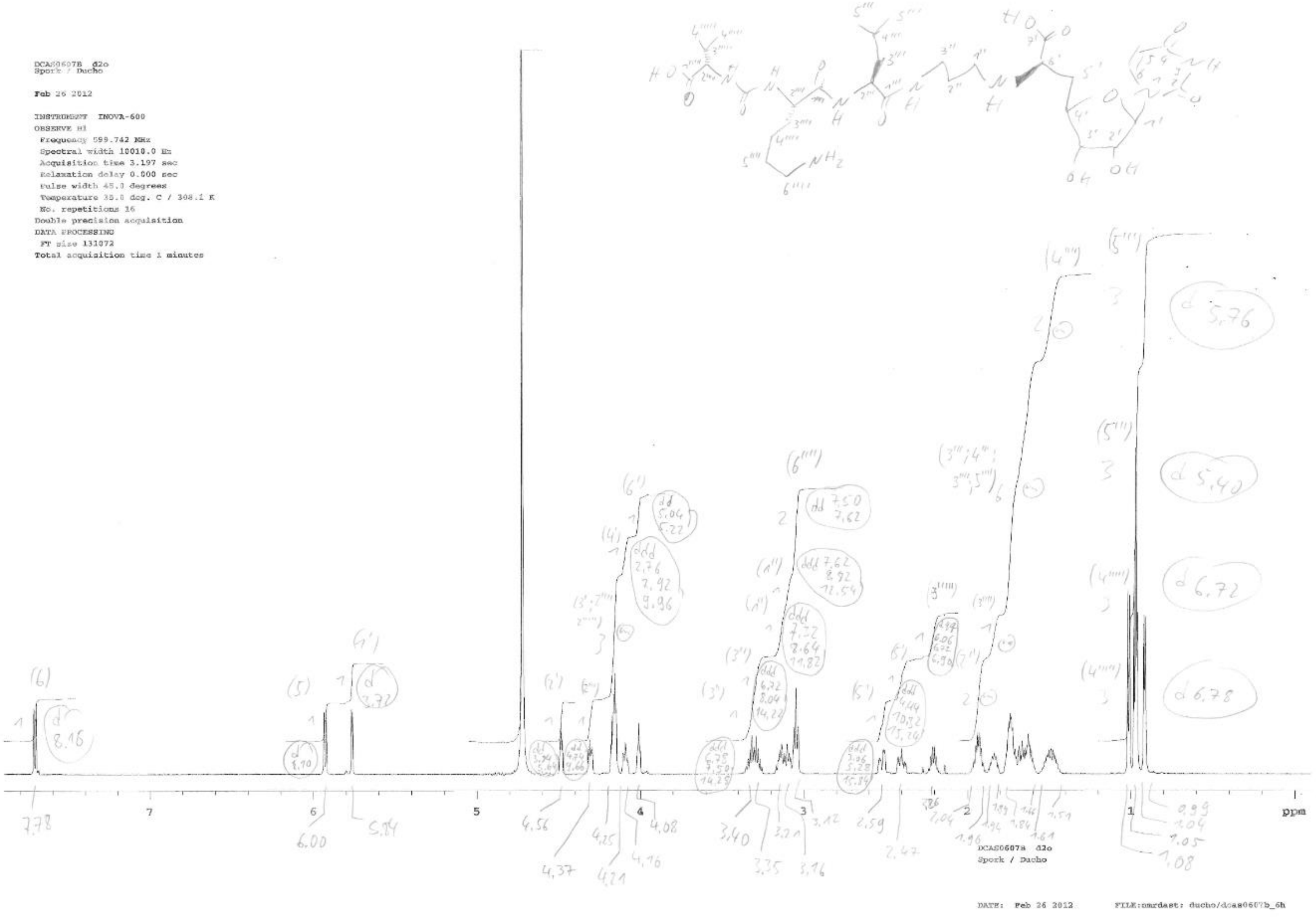

^{1}H NMR spectrum of **12** (600 MHz, D_2O, 35 °C)

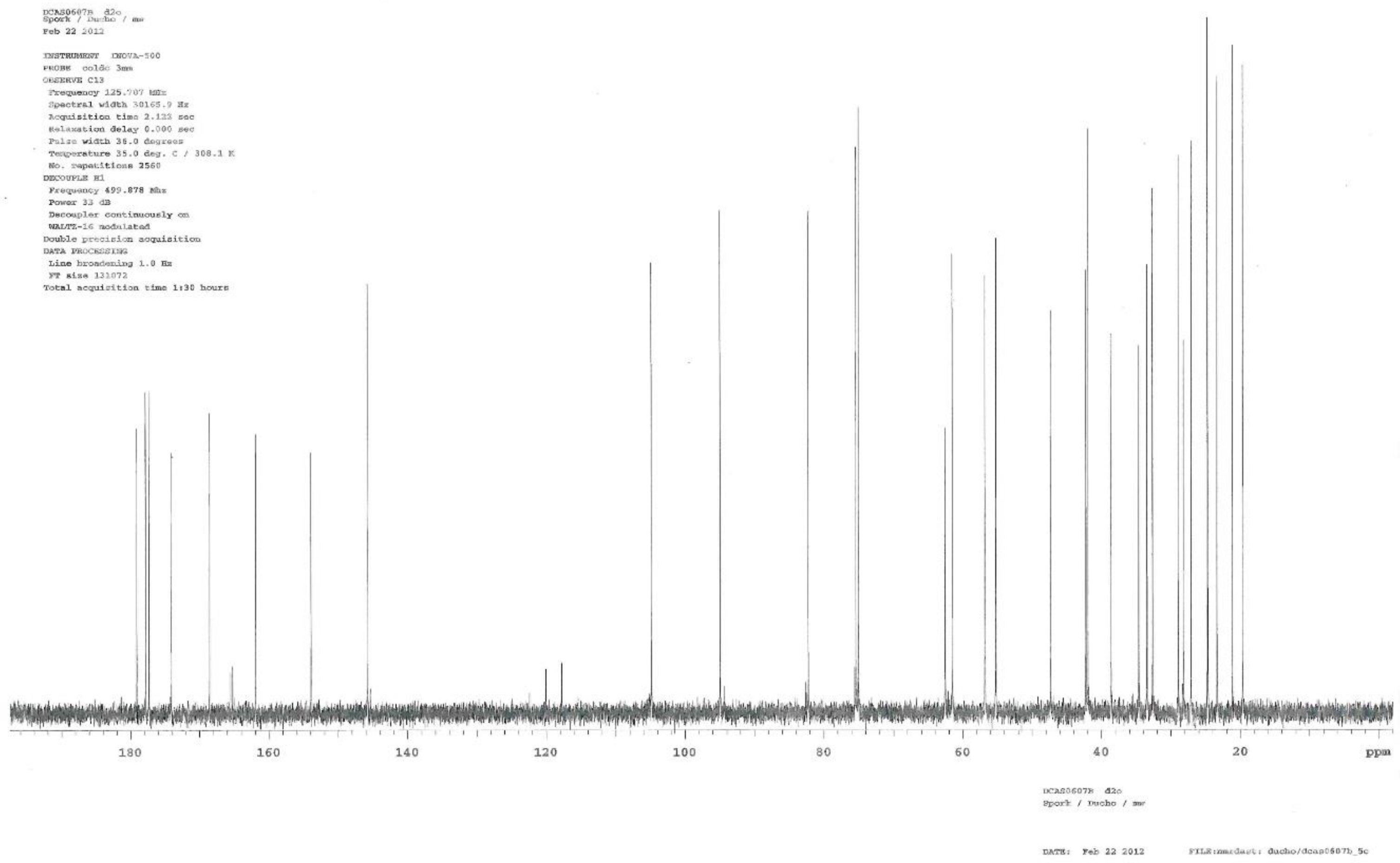

^{13}C NMR spectrum of **12** (126 MHz, D_2O, 35 °C)

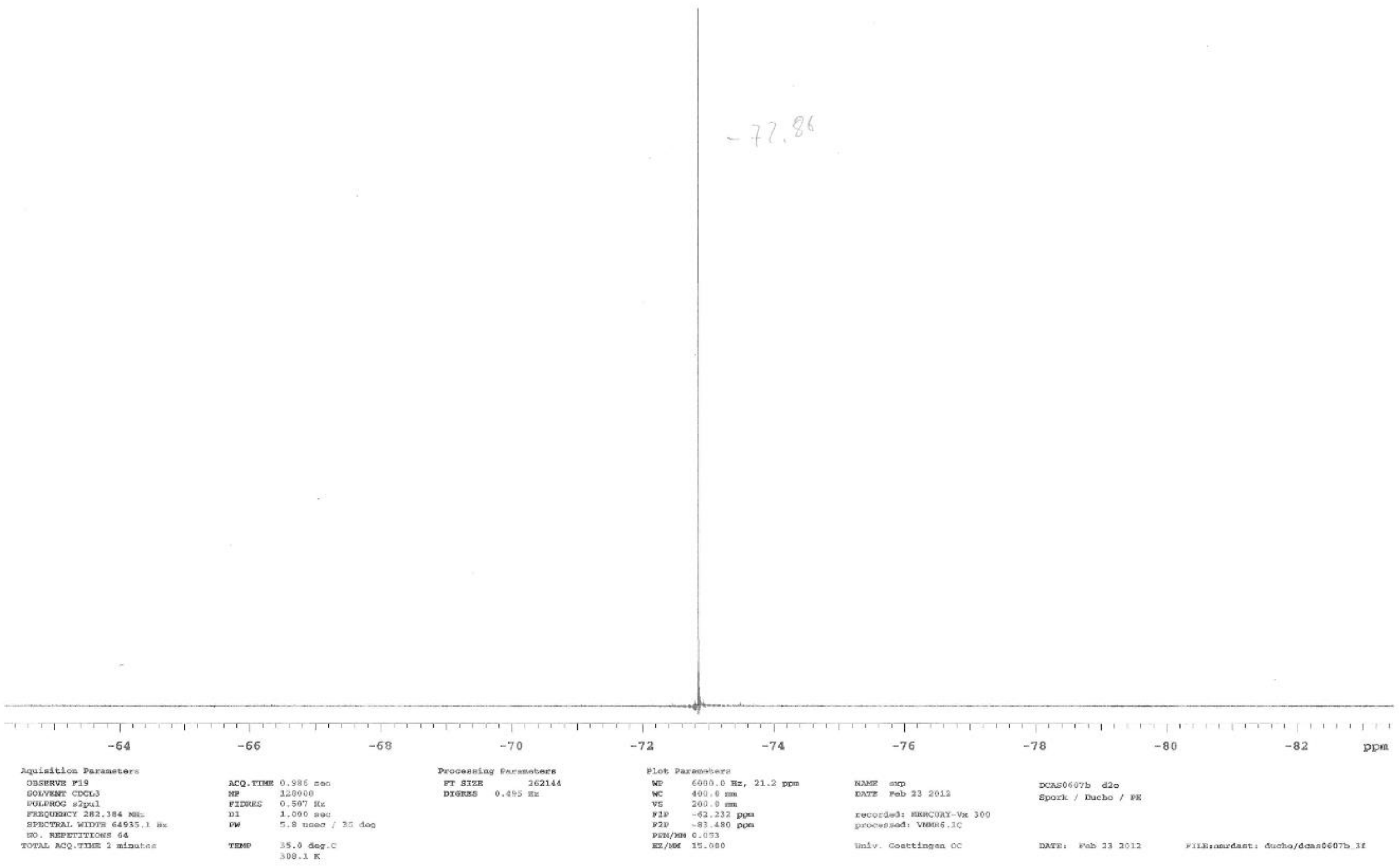

^{19}F NMR spectrum of **12** (282 MHz, D_2O, 35 °C)

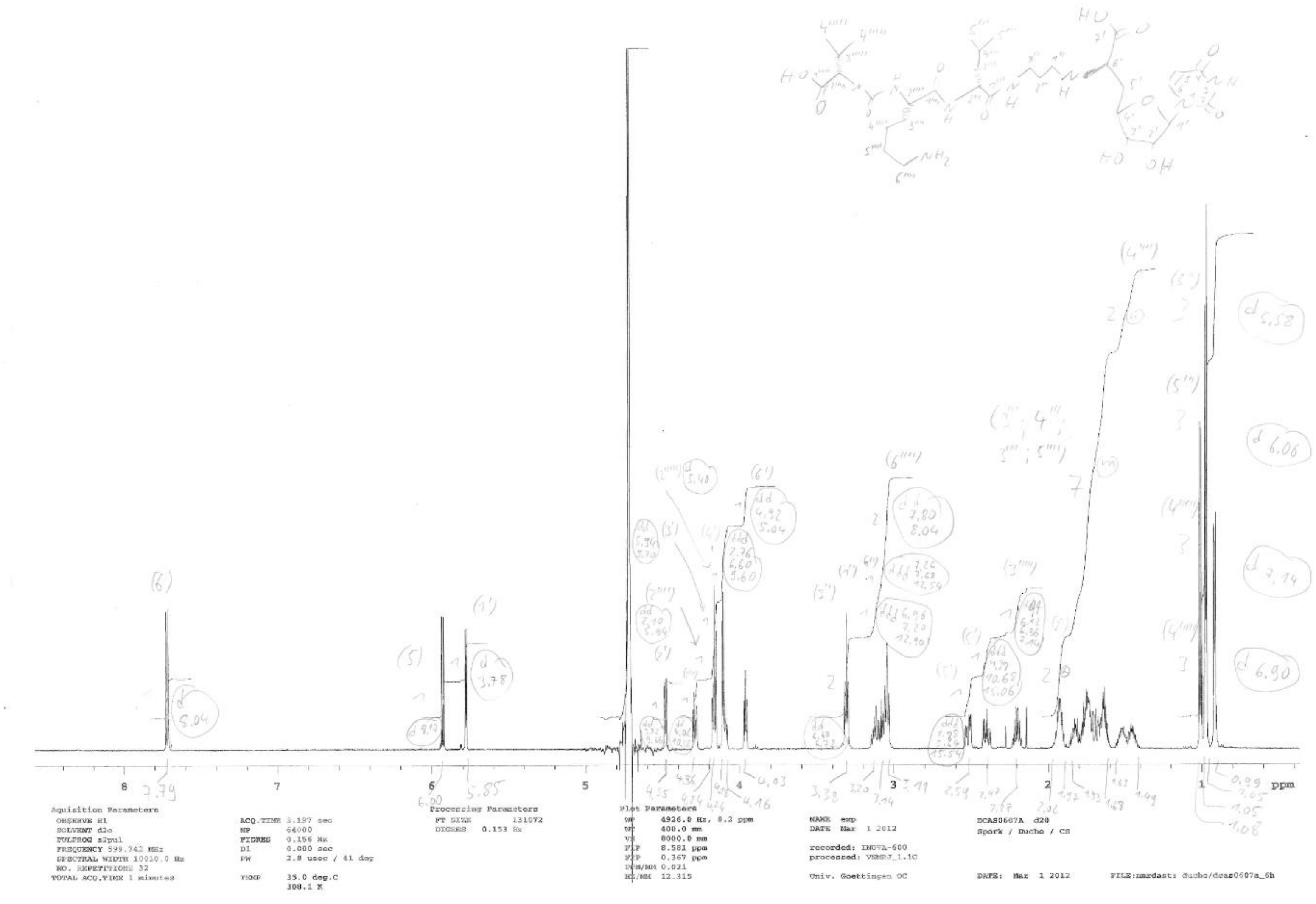

^{1}H NMR spectrum of **13** (600 MHz, D_2O, 35 °C)

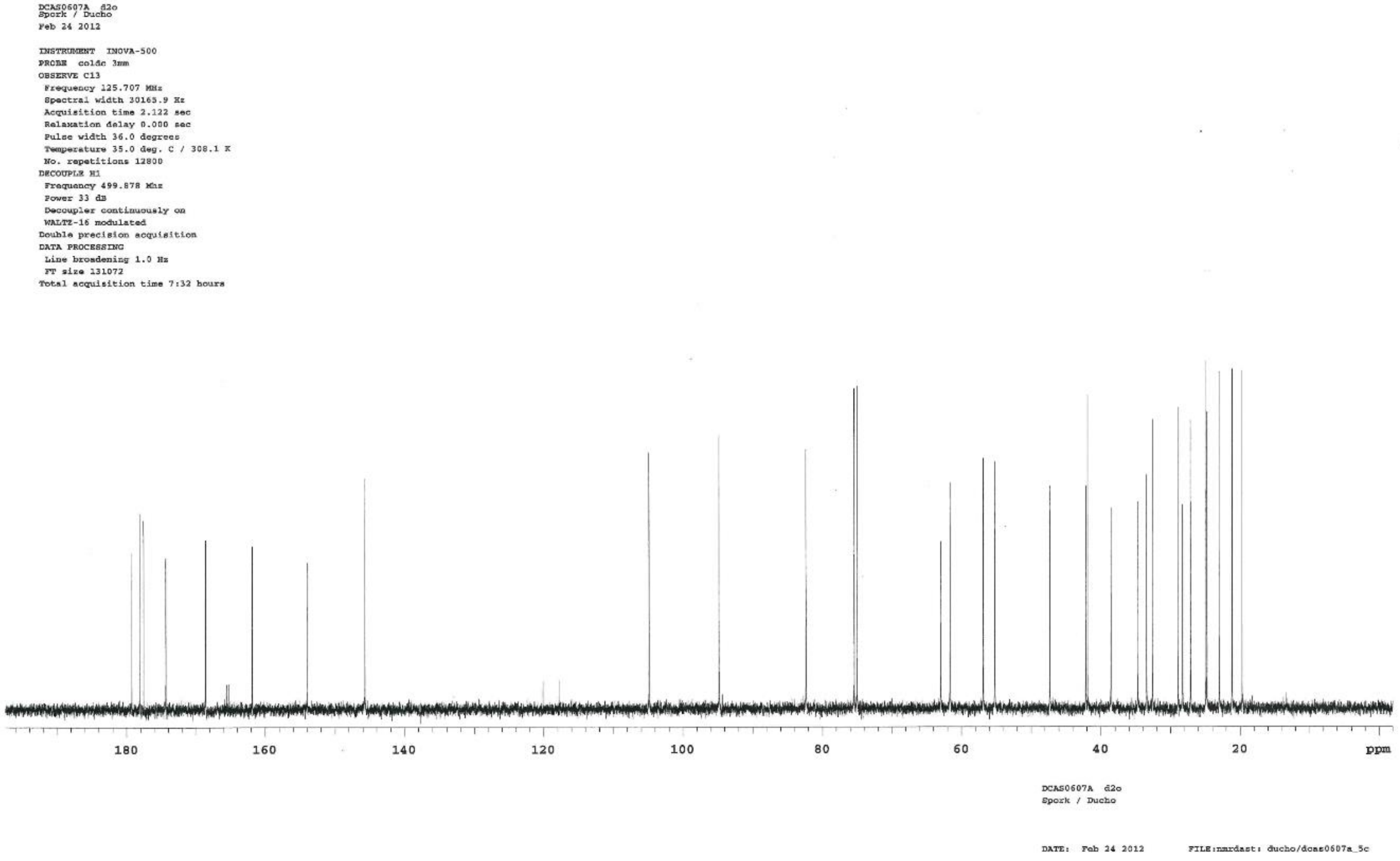

^{13}C NMR spectrum of **13** (126 MHz, D_2O, 35 °C)

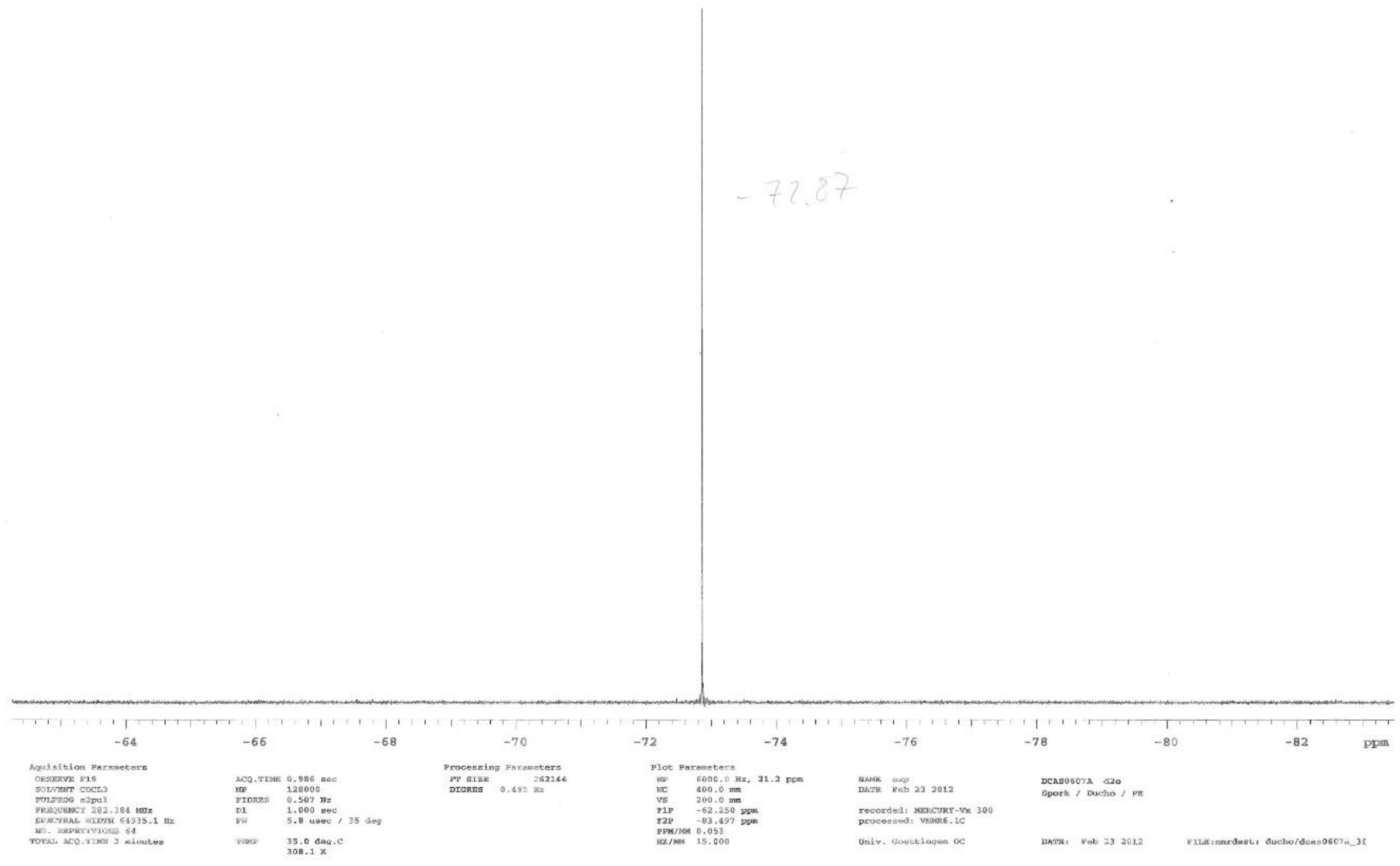

^{19}F NMR spectrum of **13** (282 MHz, D_2O, 35 °C)

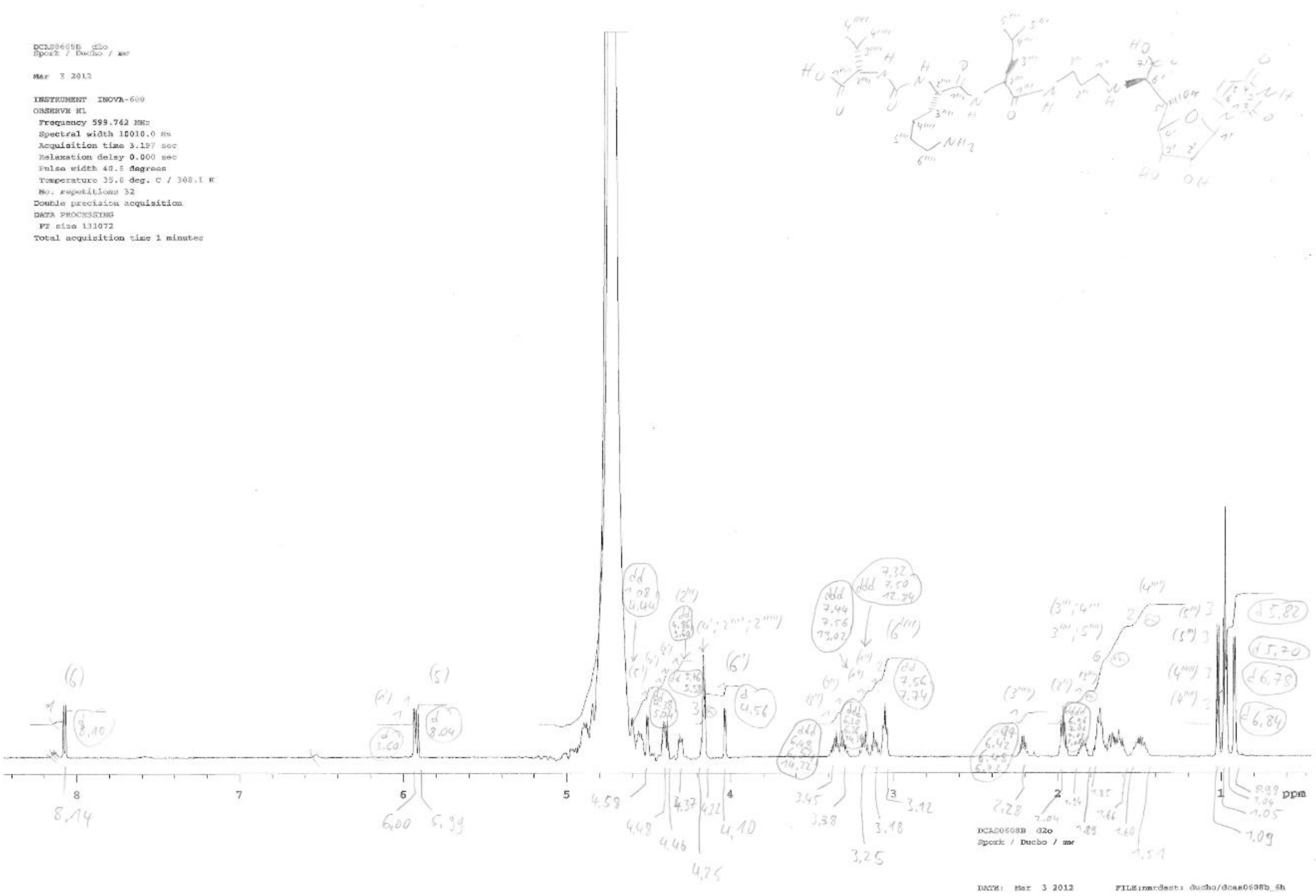

^{1}H NMR spectrum of **14** (600 MHz, D_2O, 35 °C)

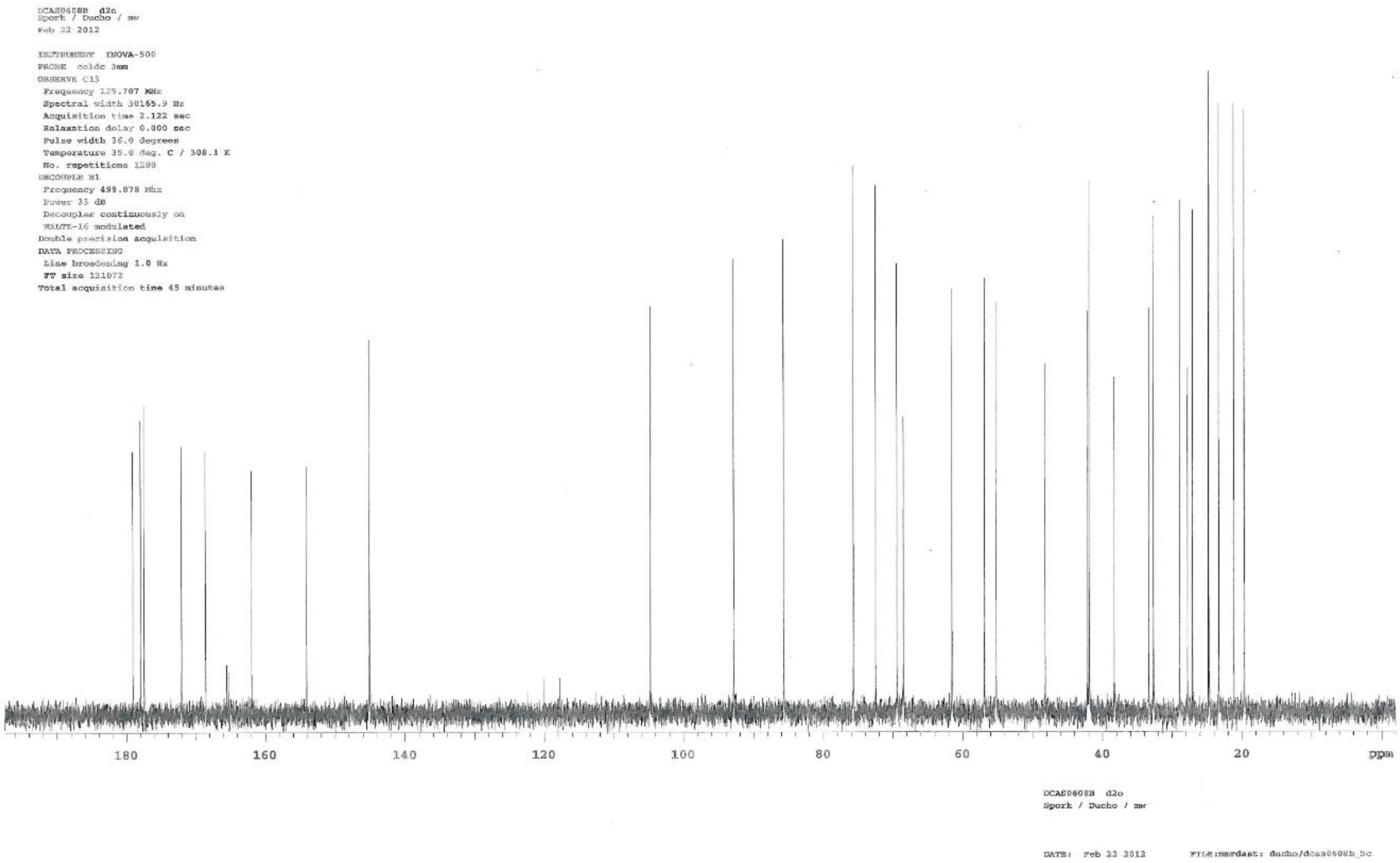

^{13}C NMR spectrum of **14** (126 MHz, D_2O, 35 °C)

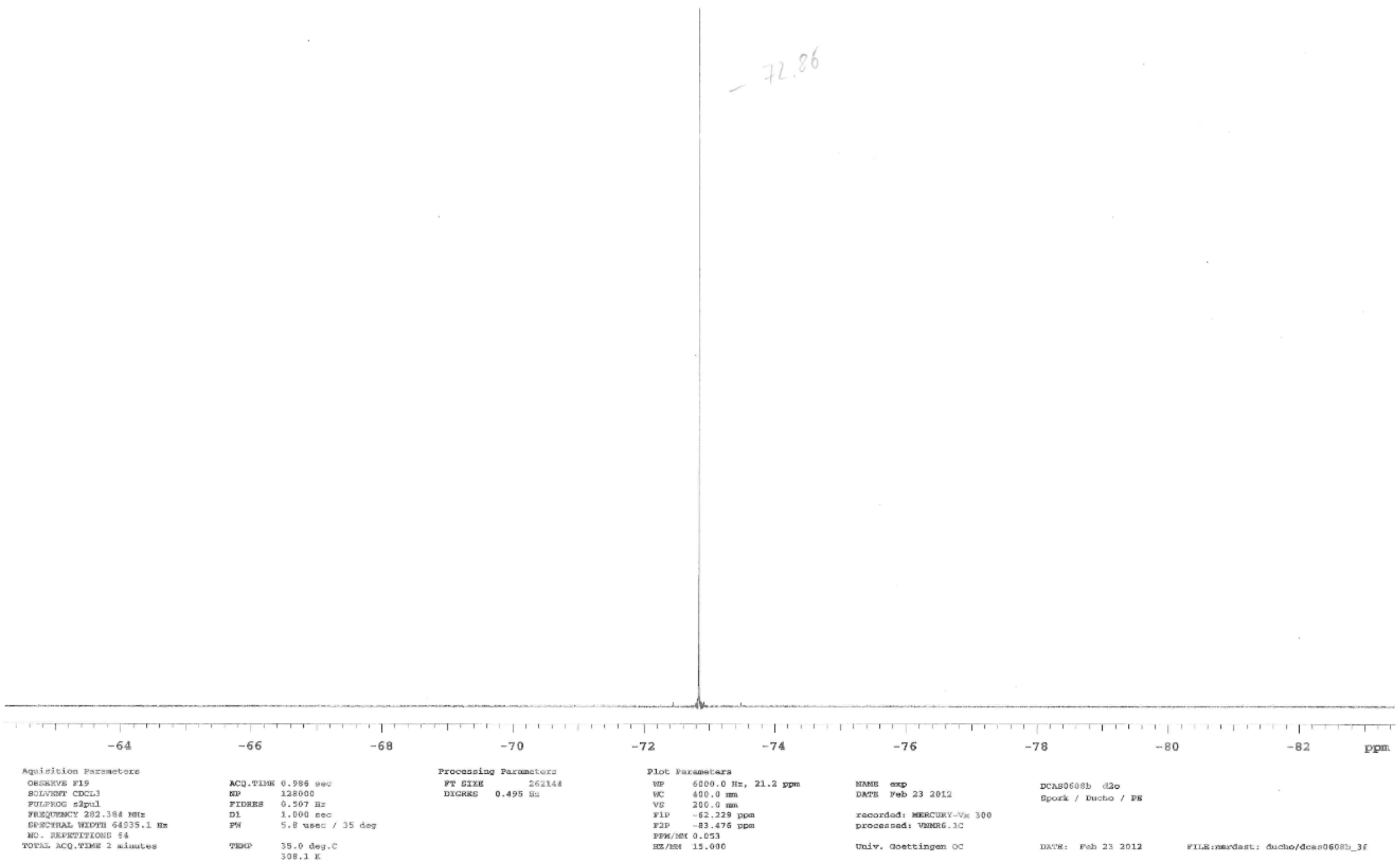

^{19}F NMR spectrum of **14** (282 MHz, D_2O, 35 °C)

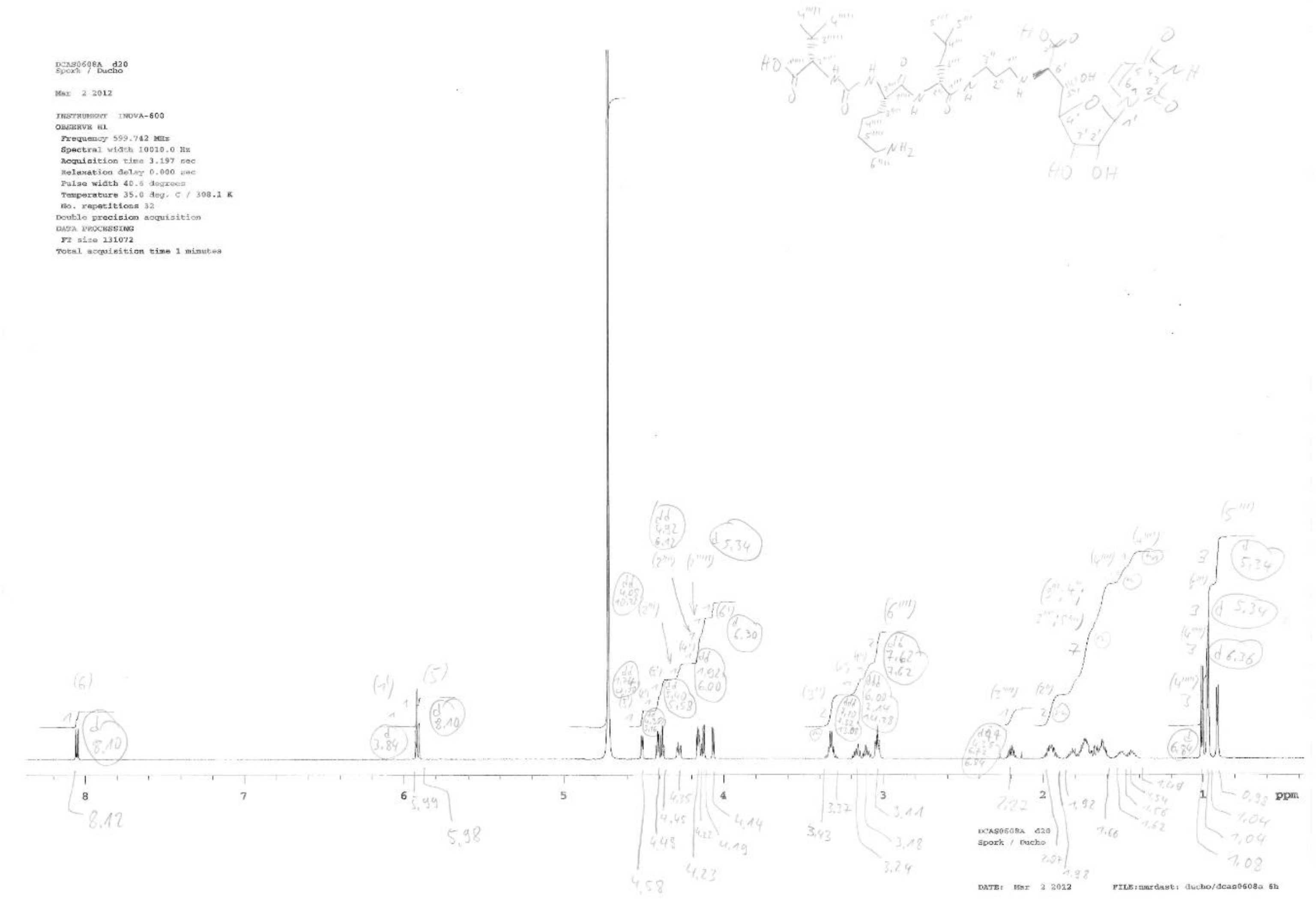

^{1}H NMR spectrum of **15** (600 MHz, D_2O, 35 °C)

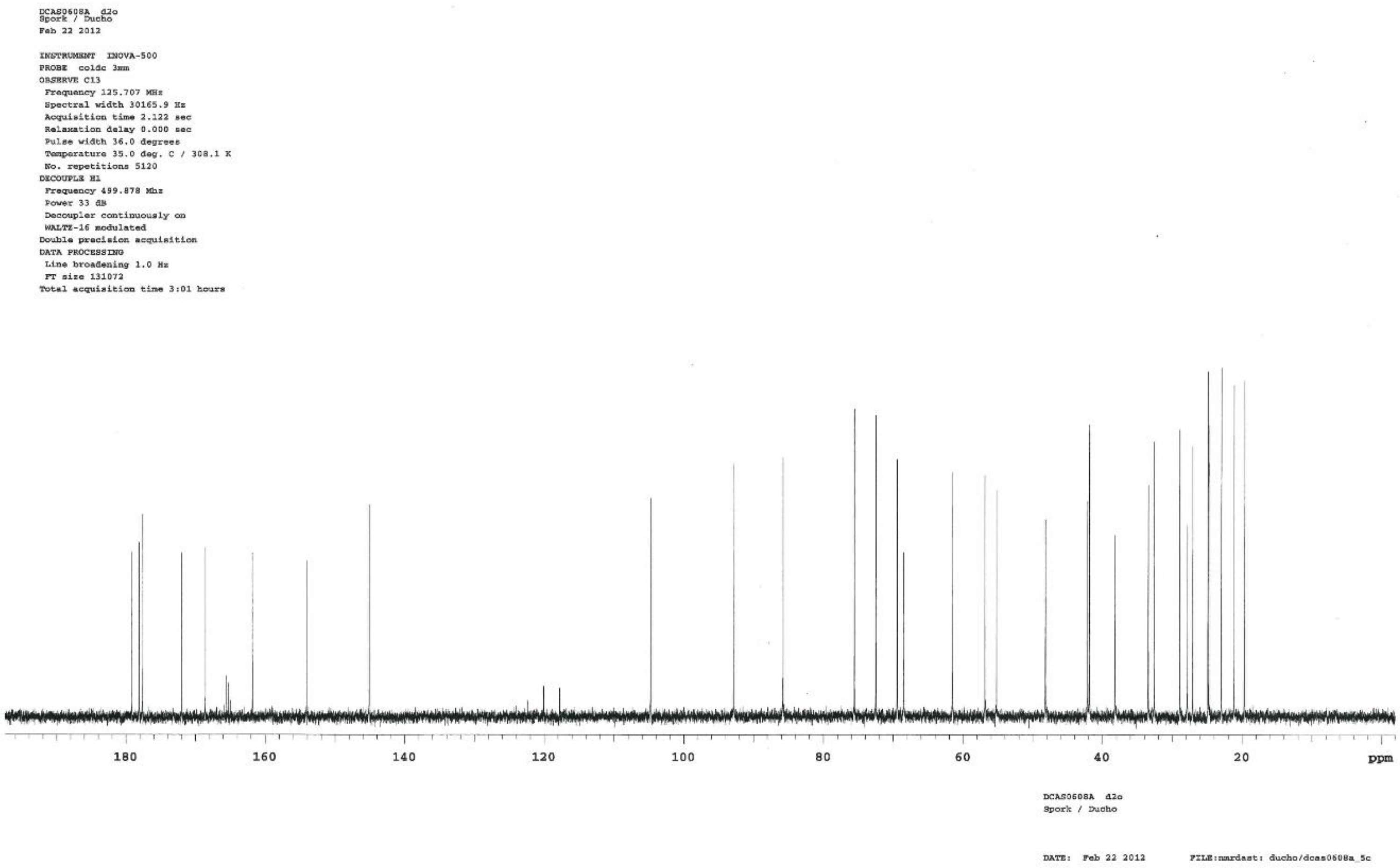

^{13}C NMR spectrum of **15** (126 MHz, D_2O, 35 °C)

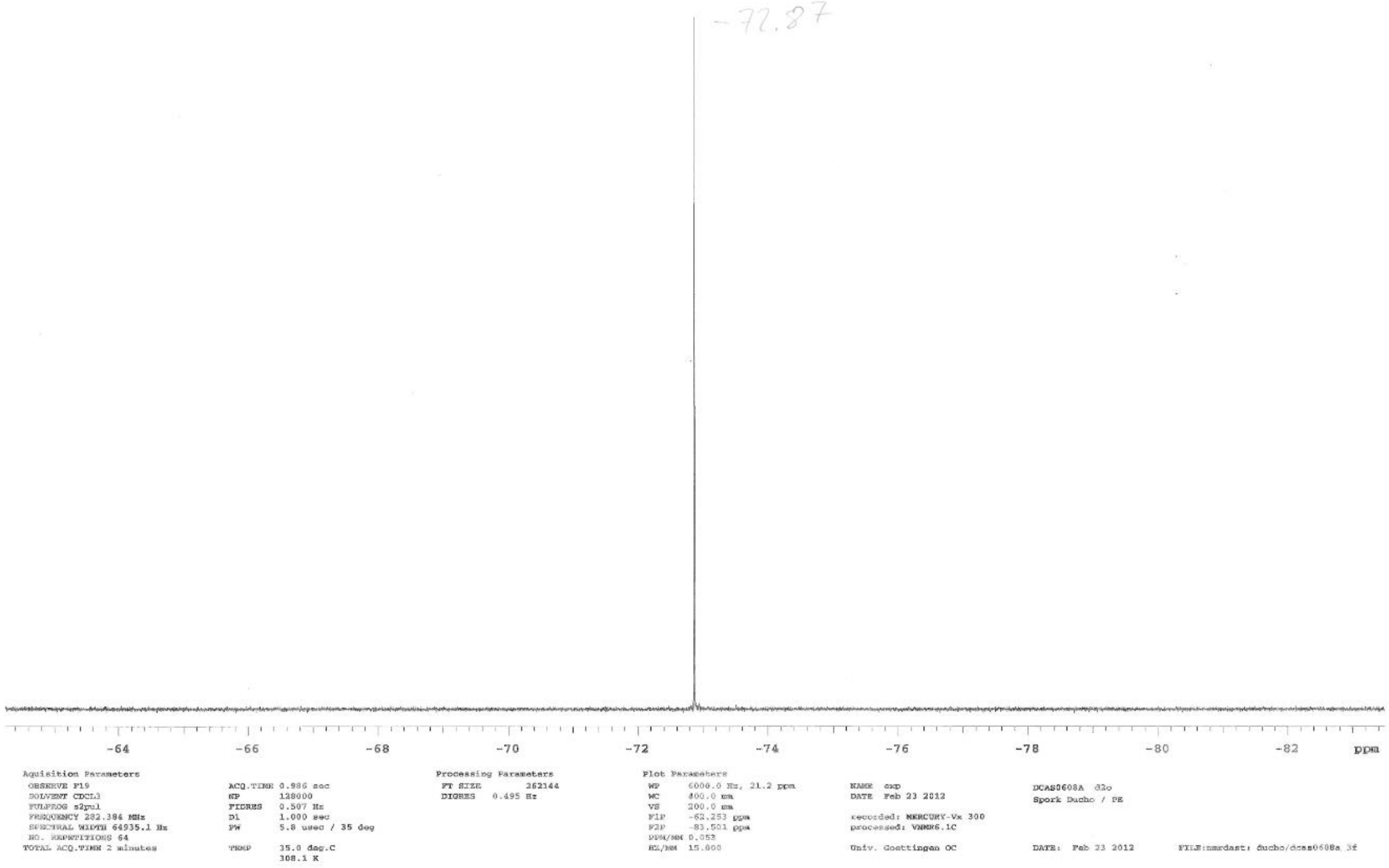

^{19}F NMR spectrum of **15** (282 MHz, D_2O, 35 °C)

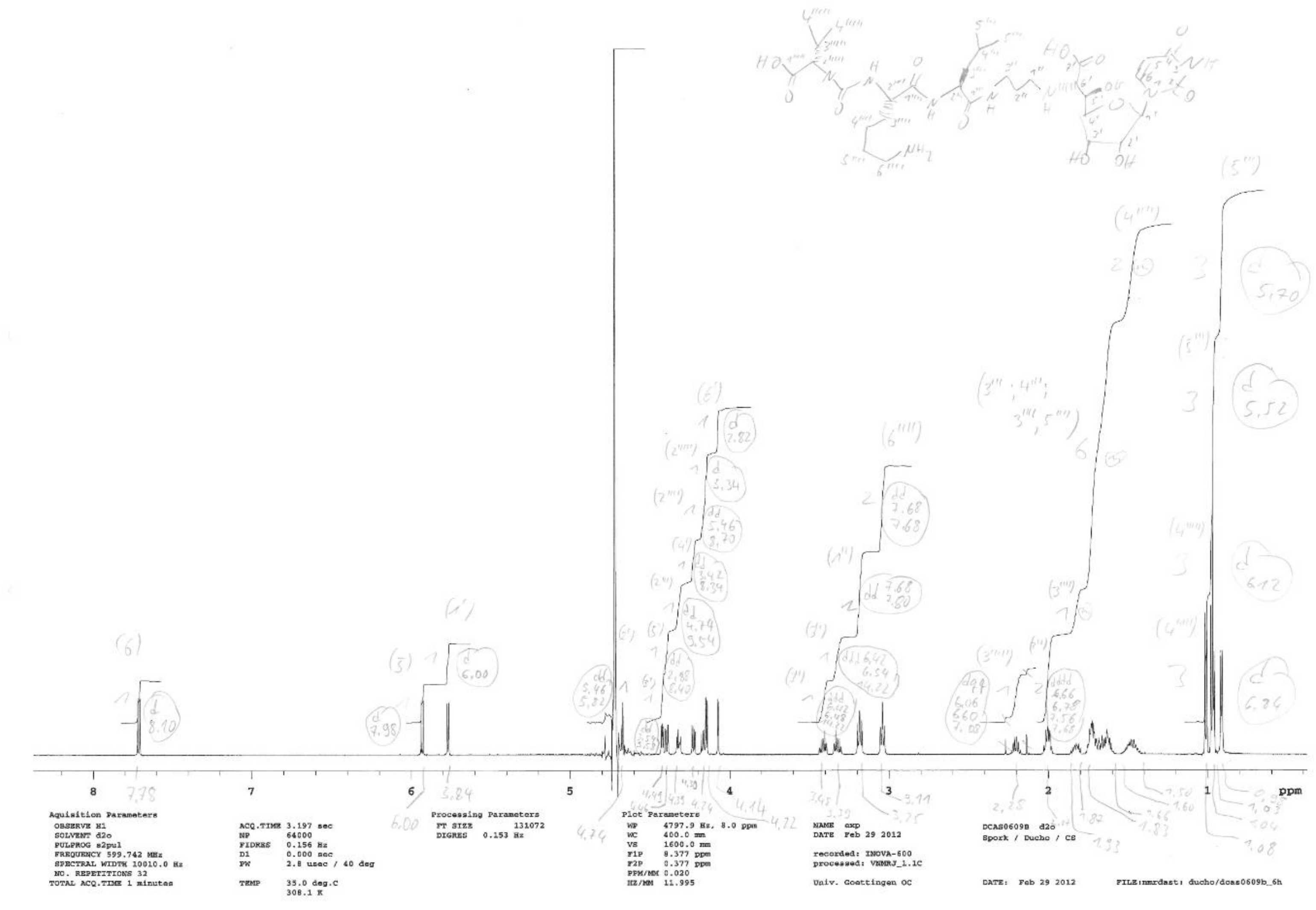

^{1}H NMR spectrum of **16** (600 MHz, D_2O, 35 °C)

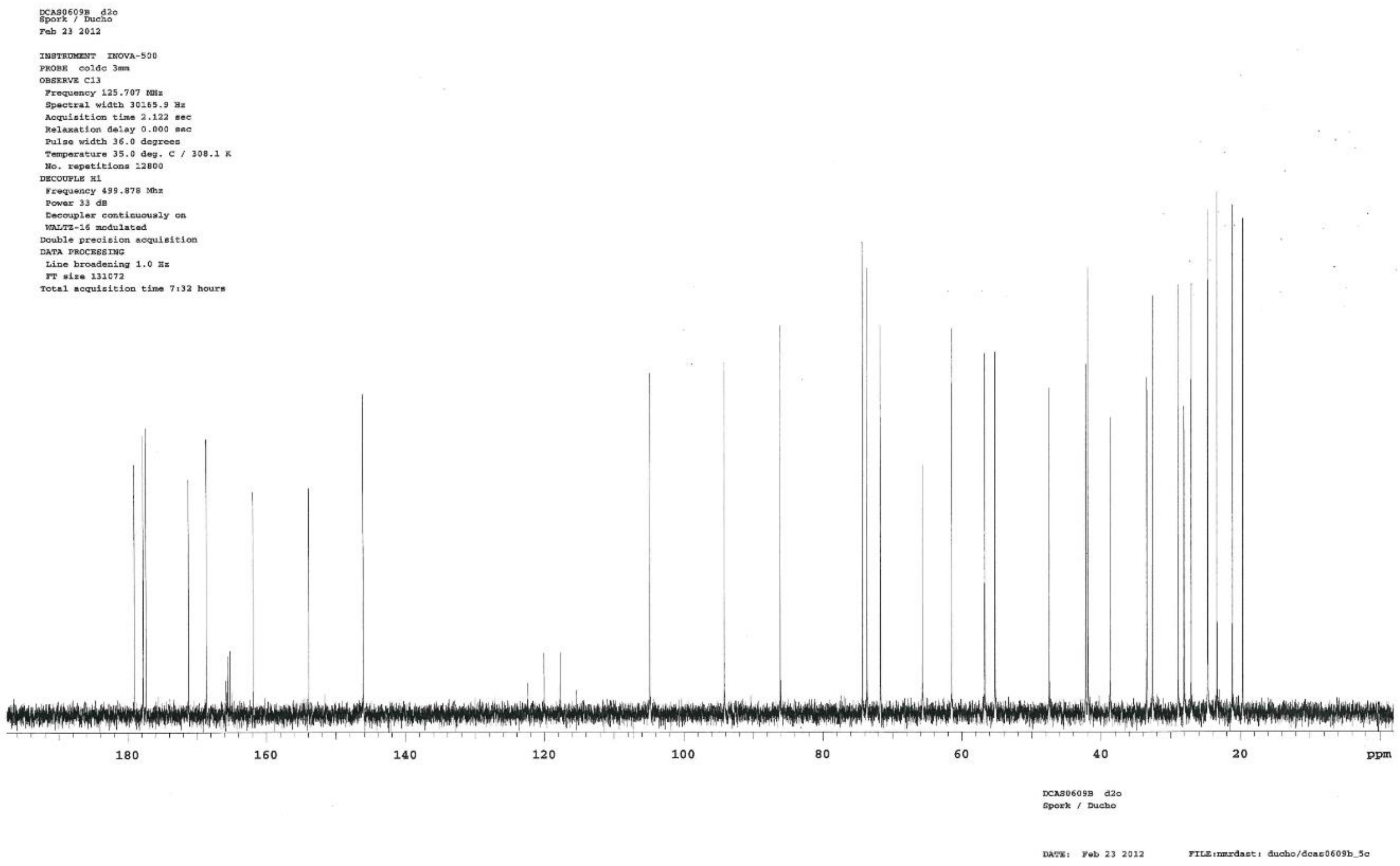

^{13}C NMR spectrum of **16** (126 MHz, D_2O, 35 °C)

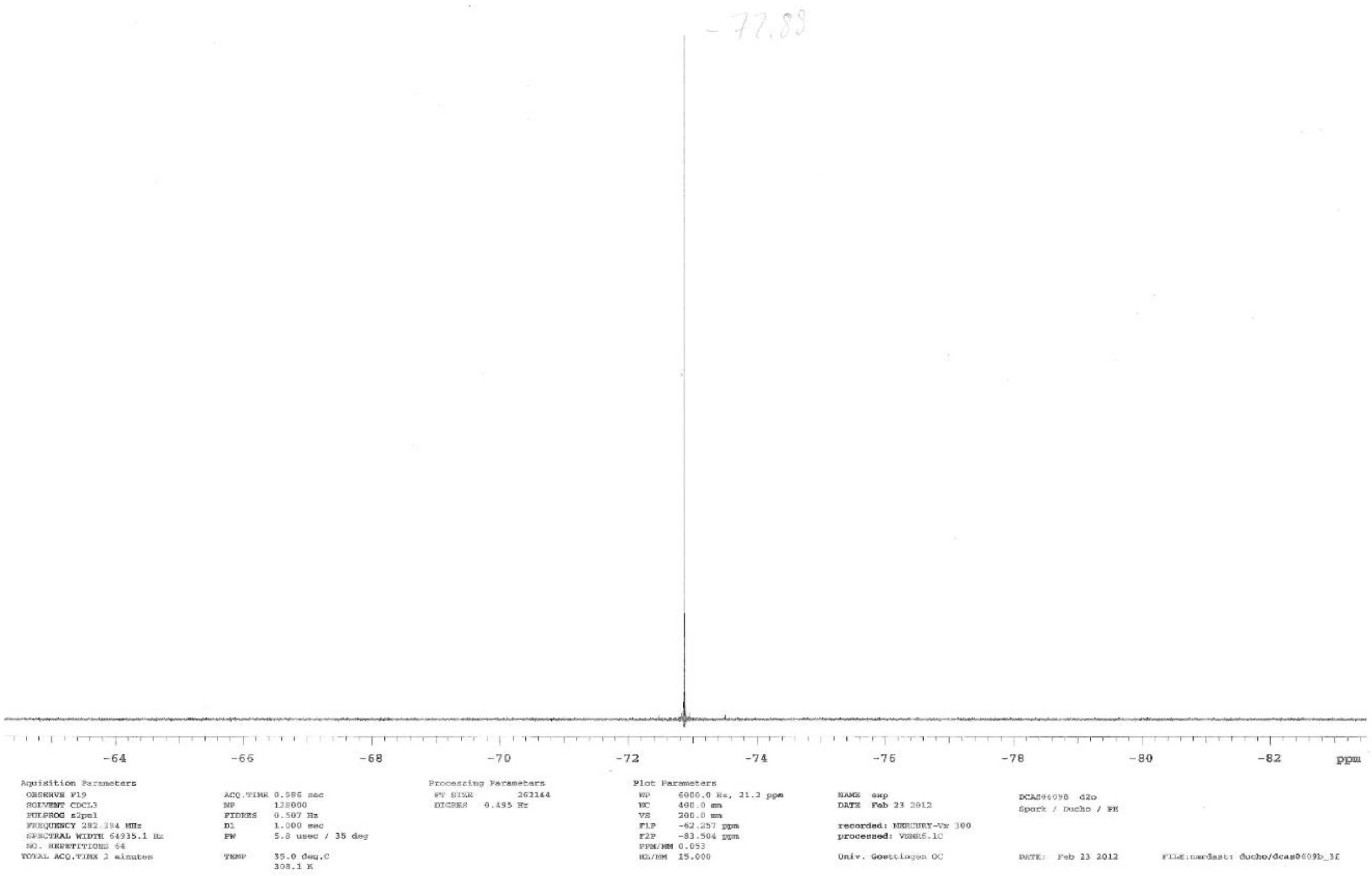

^{19}F NMR spectrum of **16** (282 MHz, D_2O, 35 °C)

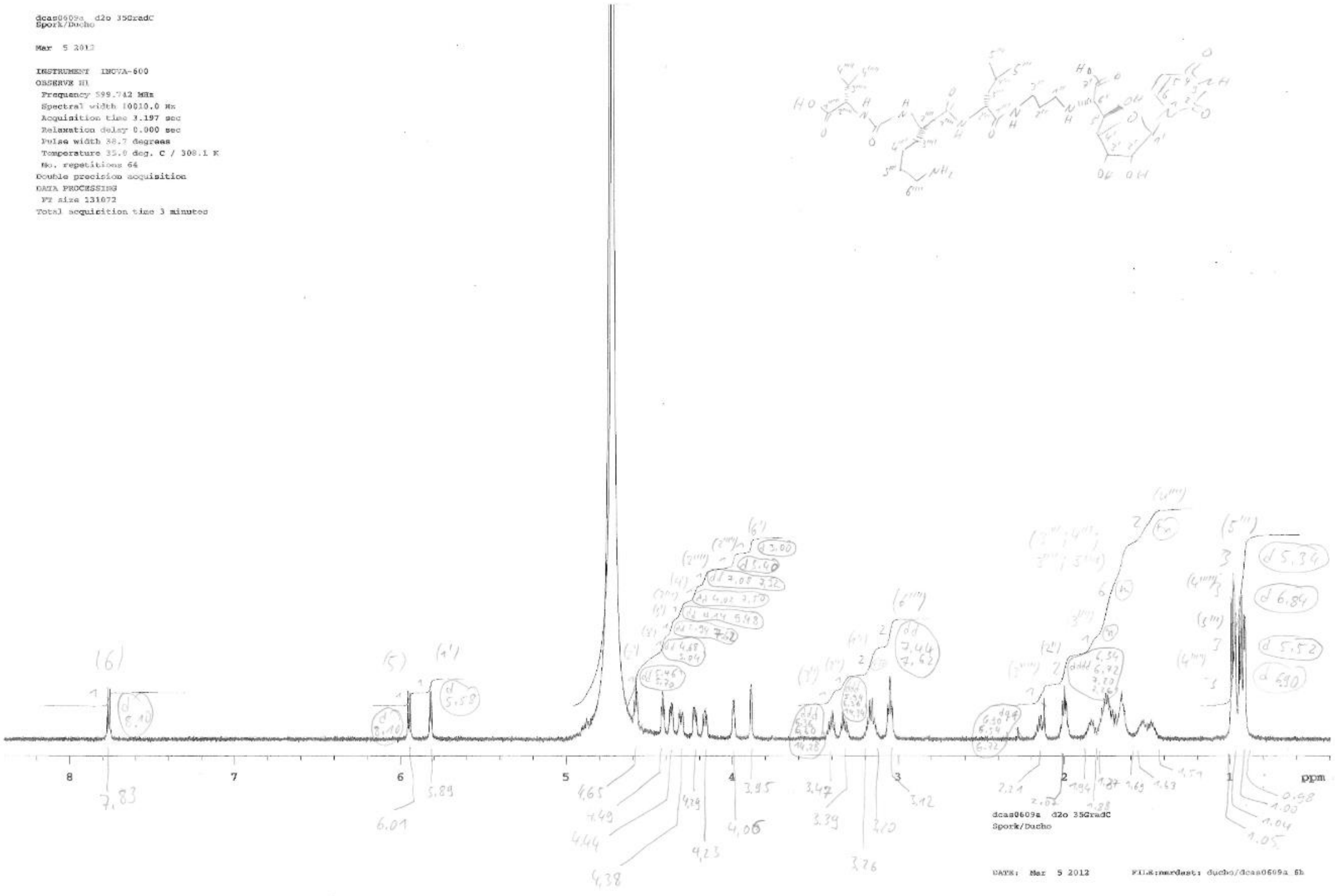

^{1}H NMR spectrum of **17** (600 MHz, D_2O, 35 °C)

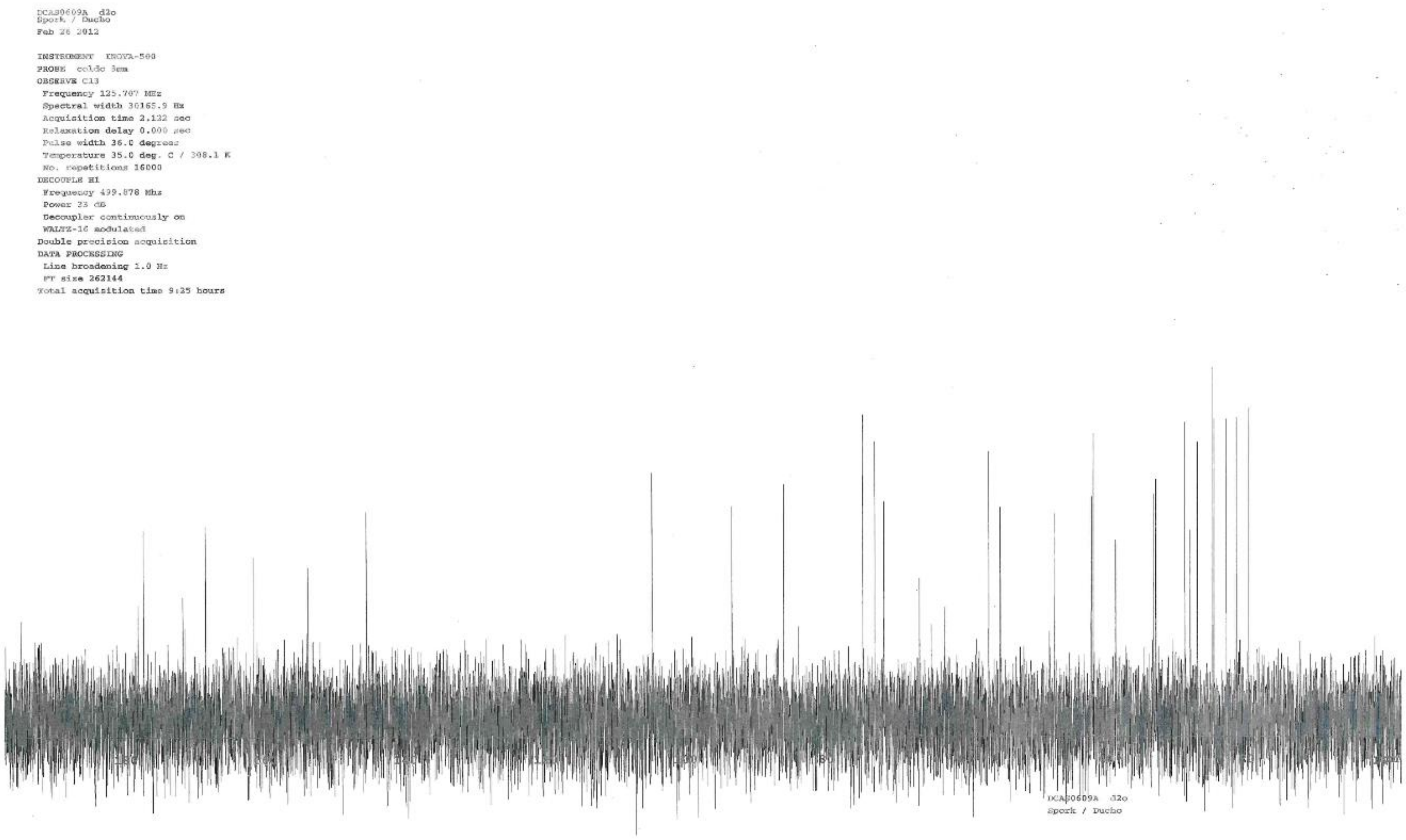

^{13}C NMR spectrum of **17** (126 MHz, D_2O, 35 °C)

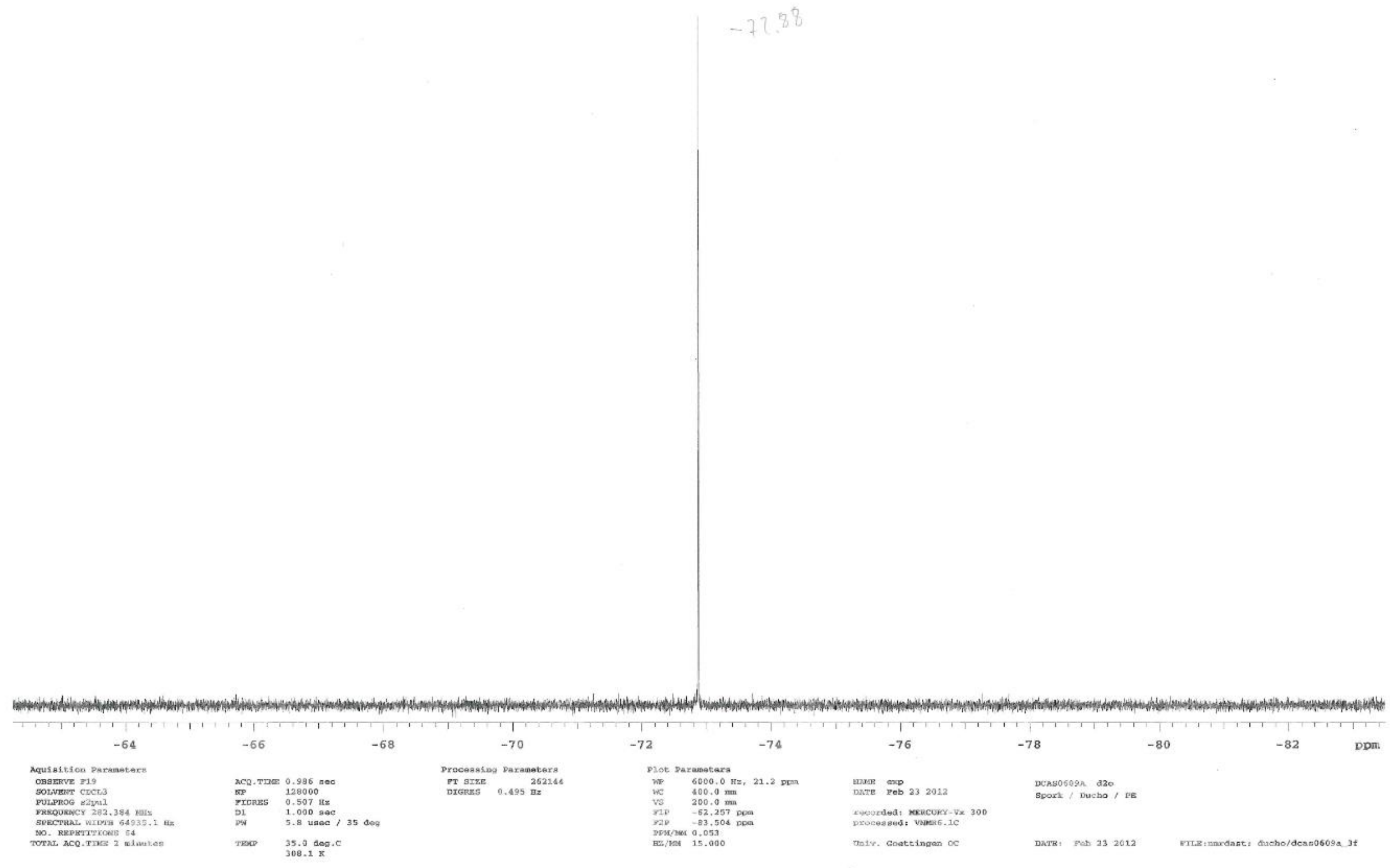

^{19}F NMR spectrum of **17** (282 MHz, D_2O, 35 °C)

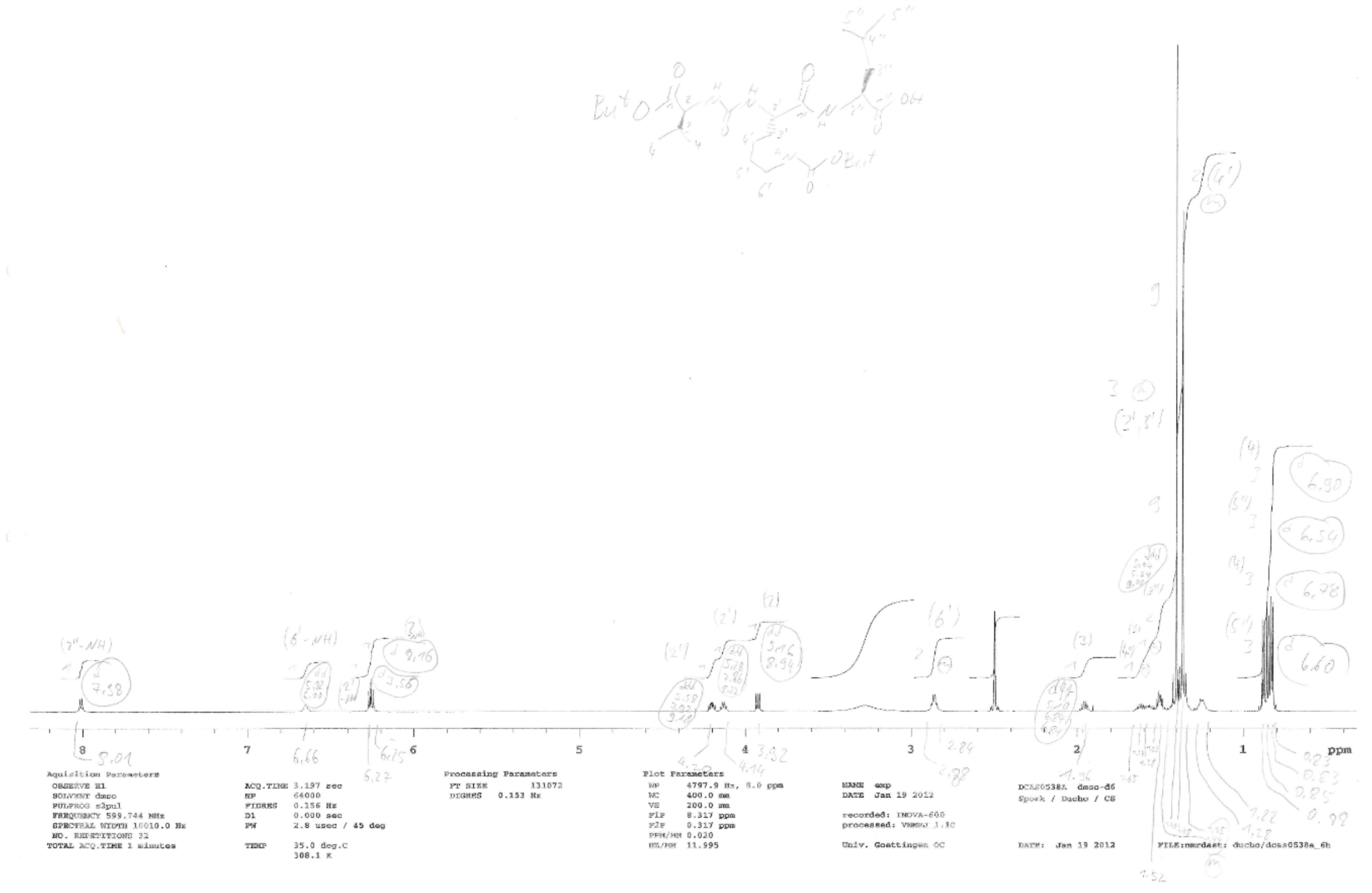

^{1}H NMR spectrum of **18** (600 MHz, DMSO-d_6, 35 °C)

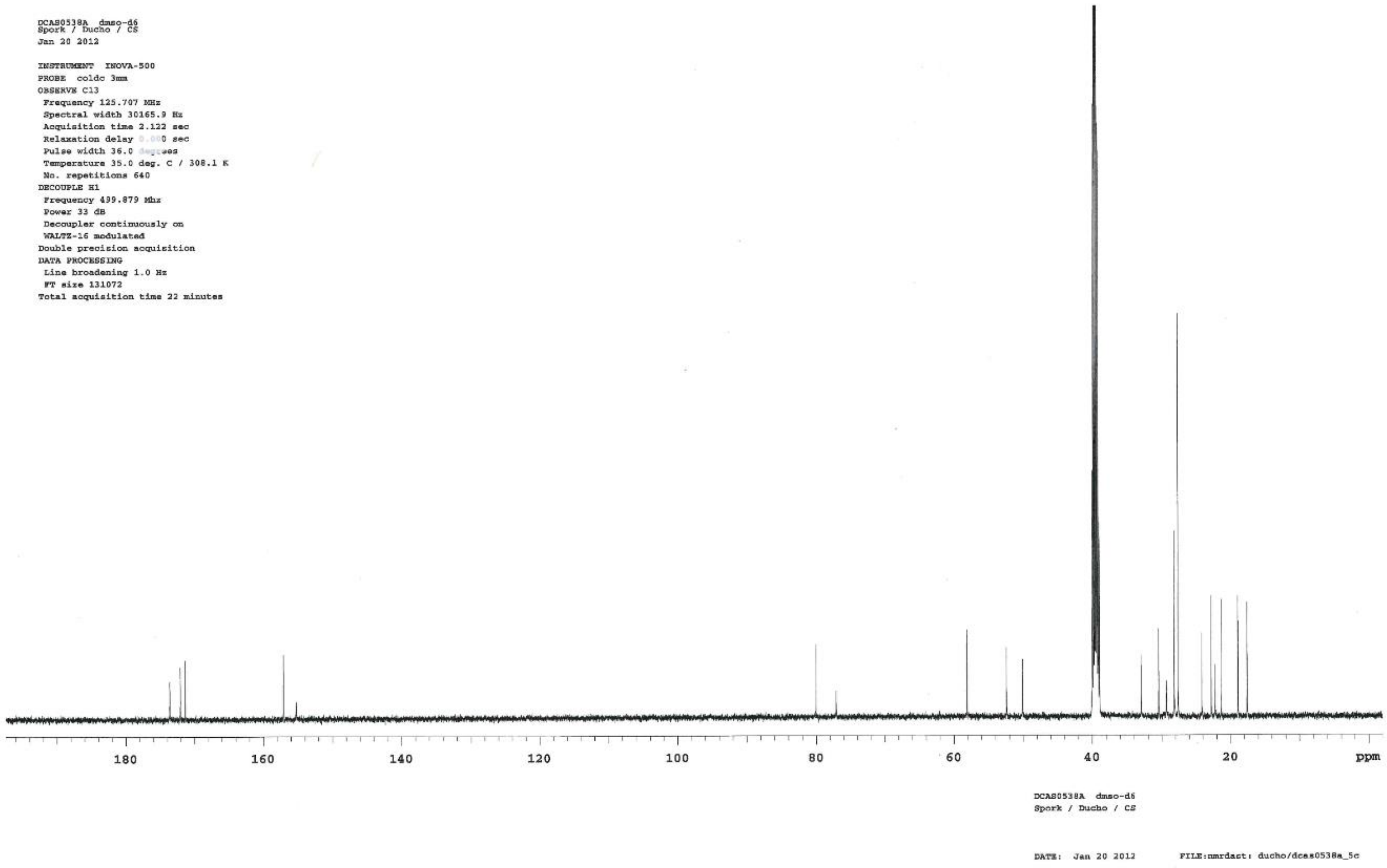

^{13}C NMR spectrum of **18** (126 MHz, DMSO-d_6, 35 °C)

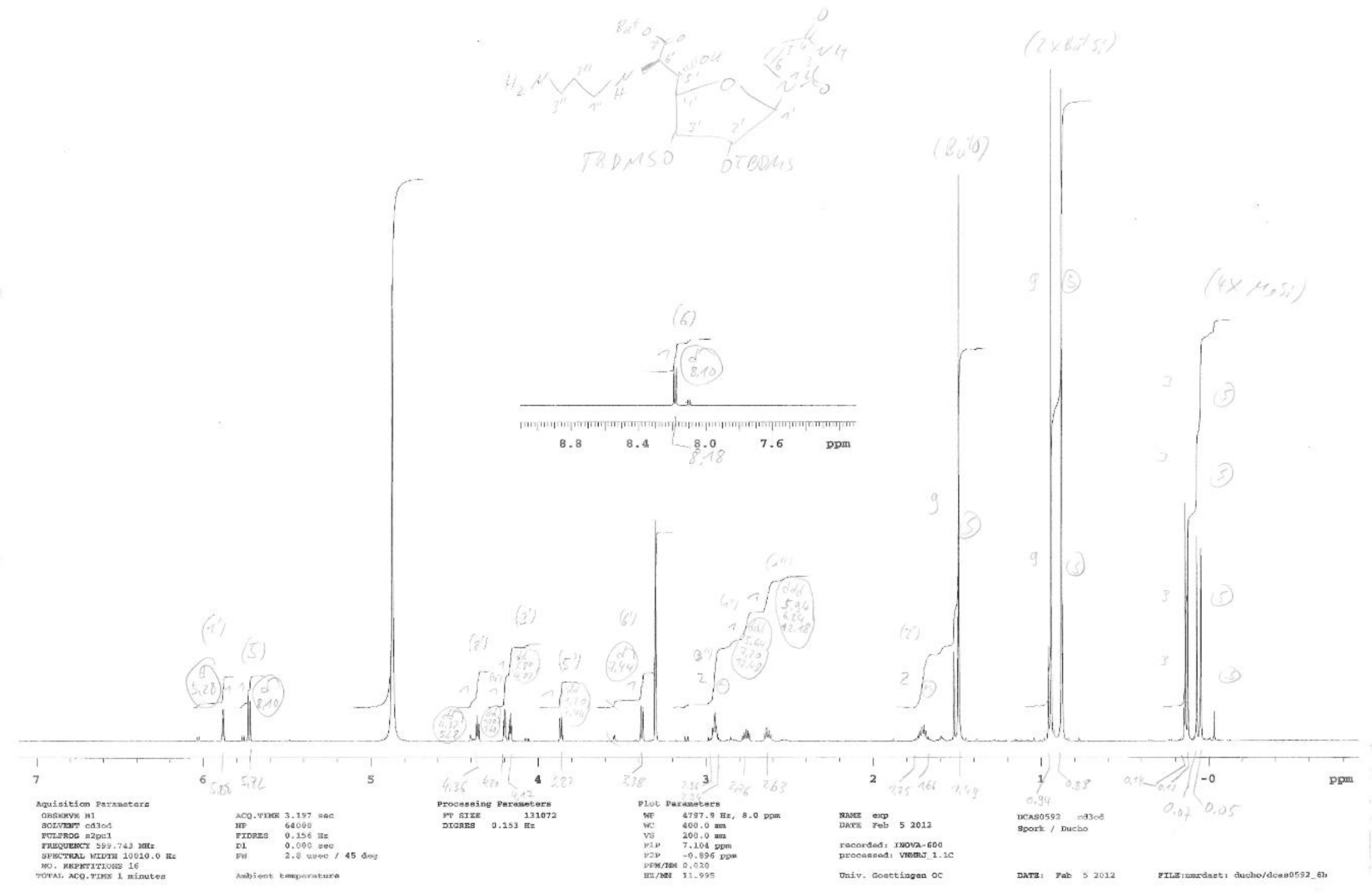

^{1}H NMR spectrum of **21** (600 MHz, CD_3OD)

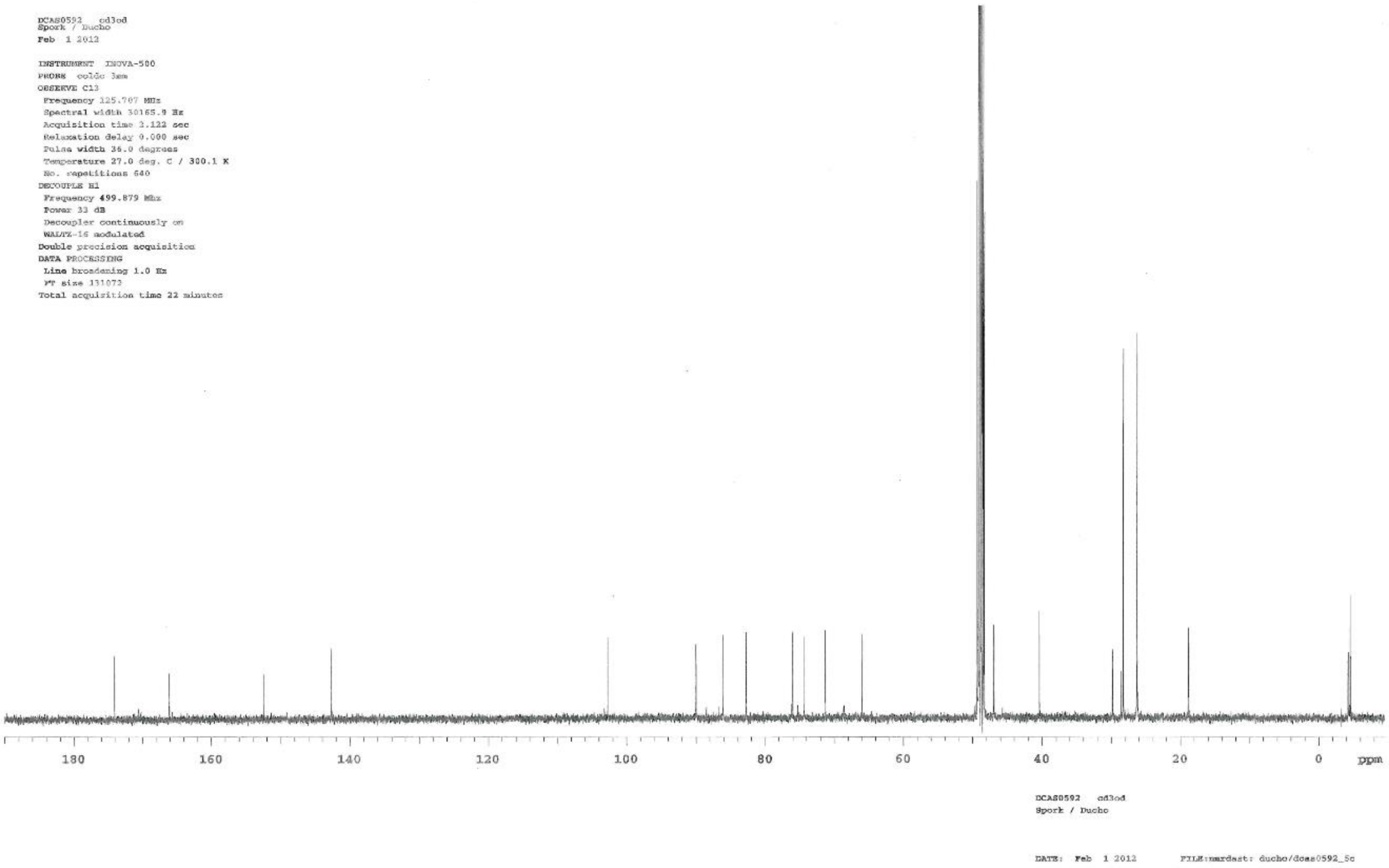

^{13}C NMR spectrum of **21** (126 MHz, CD_3OD)

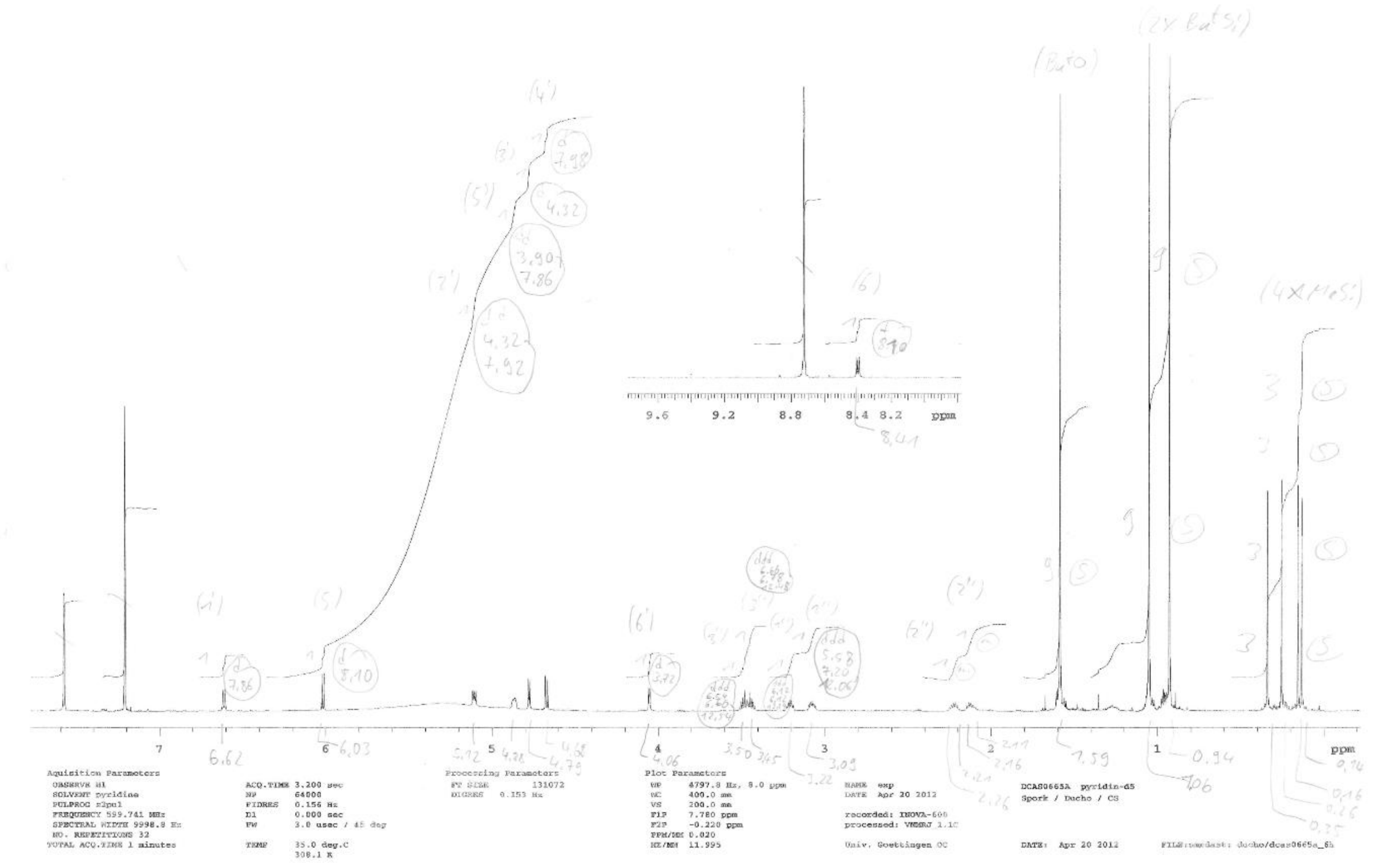

^{1}H NMR spectrum of **22** (600 MHz, pyridine-d$_5$, 35 °C)

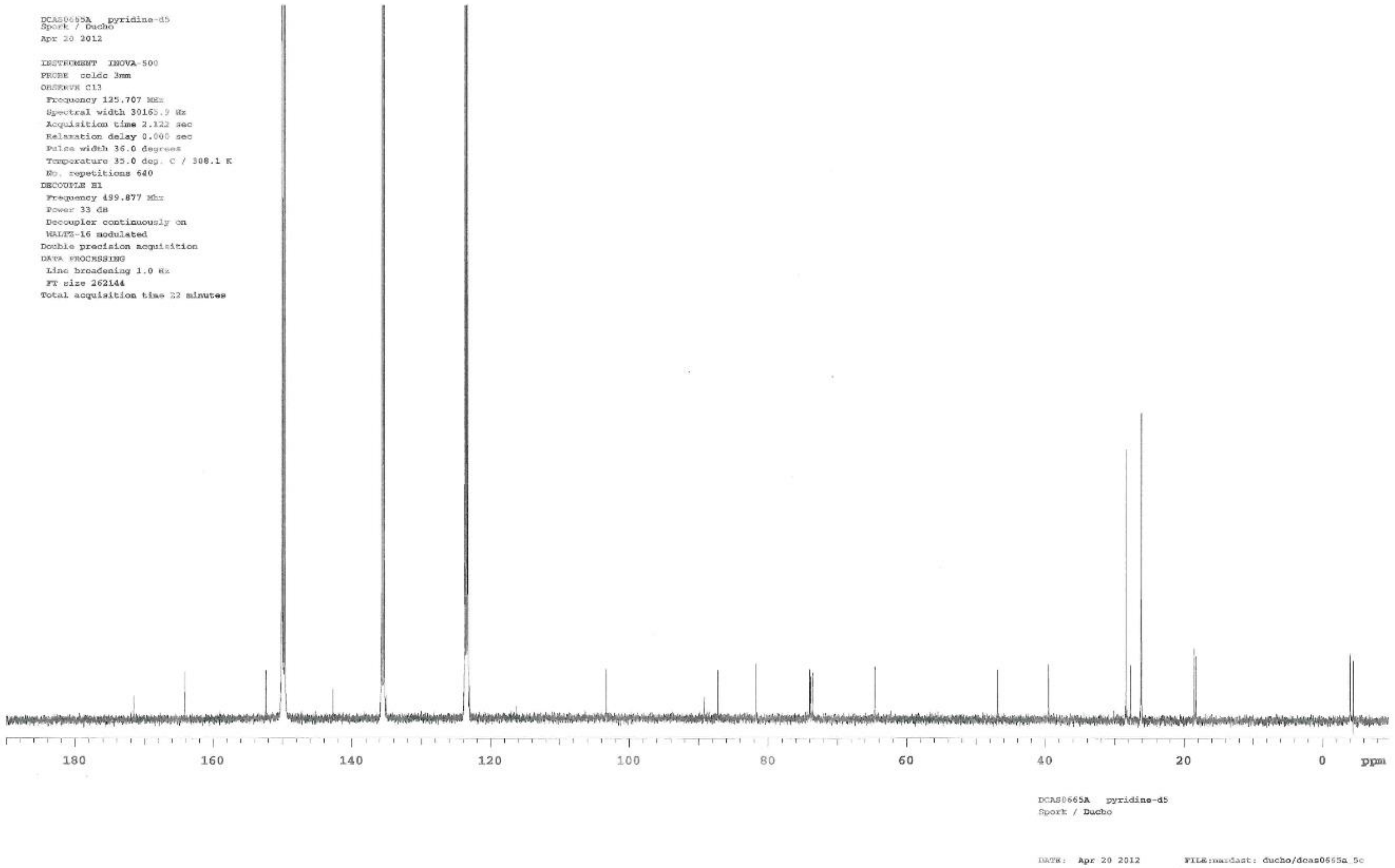

^{13}C NMR spectrum of **22** (126 MHz, pyridine-d_5, 35 °C)

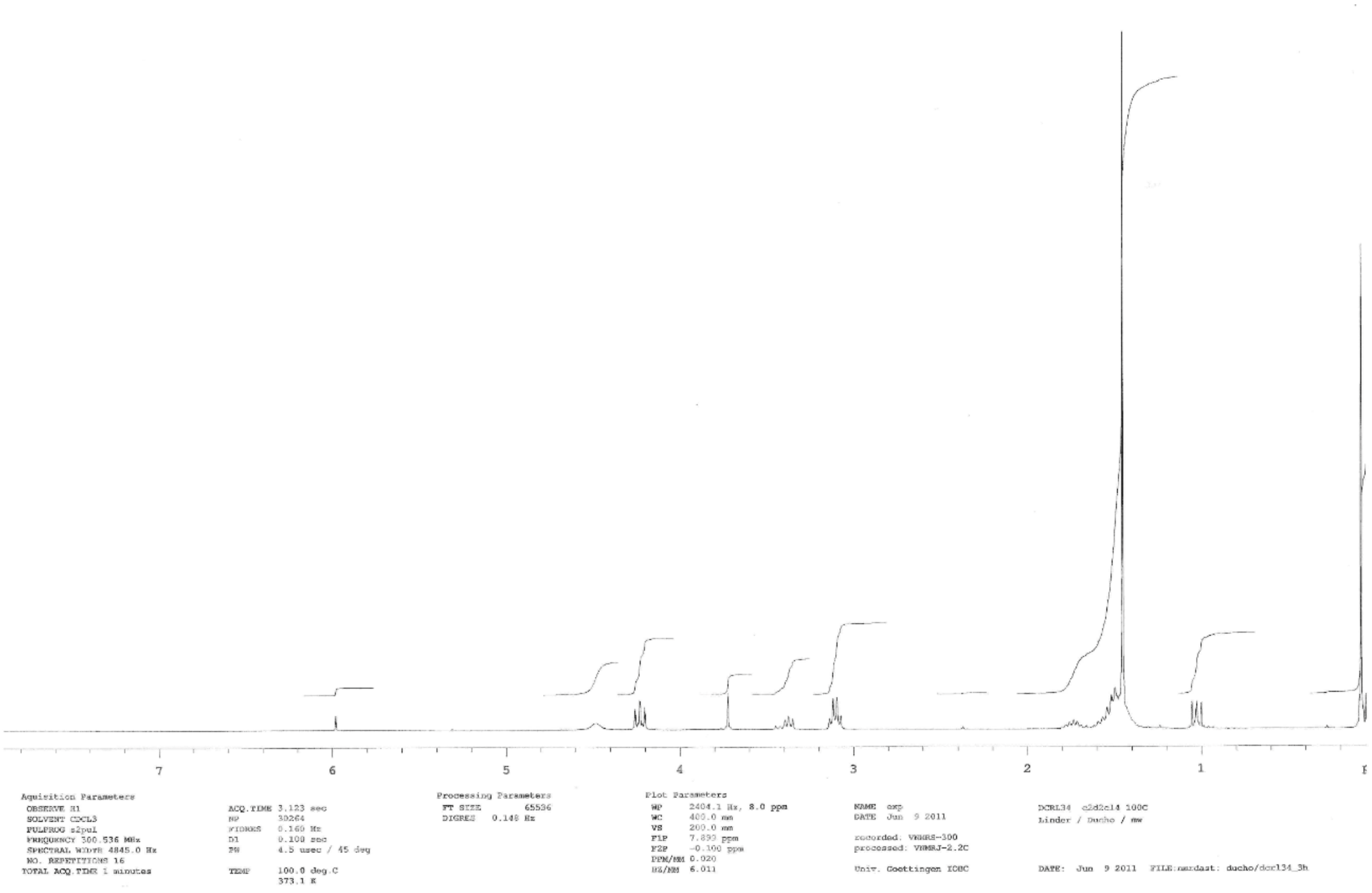

^{1}H-NMR of **24** (300 MHz, CD_2Cl_4, 100 °C)

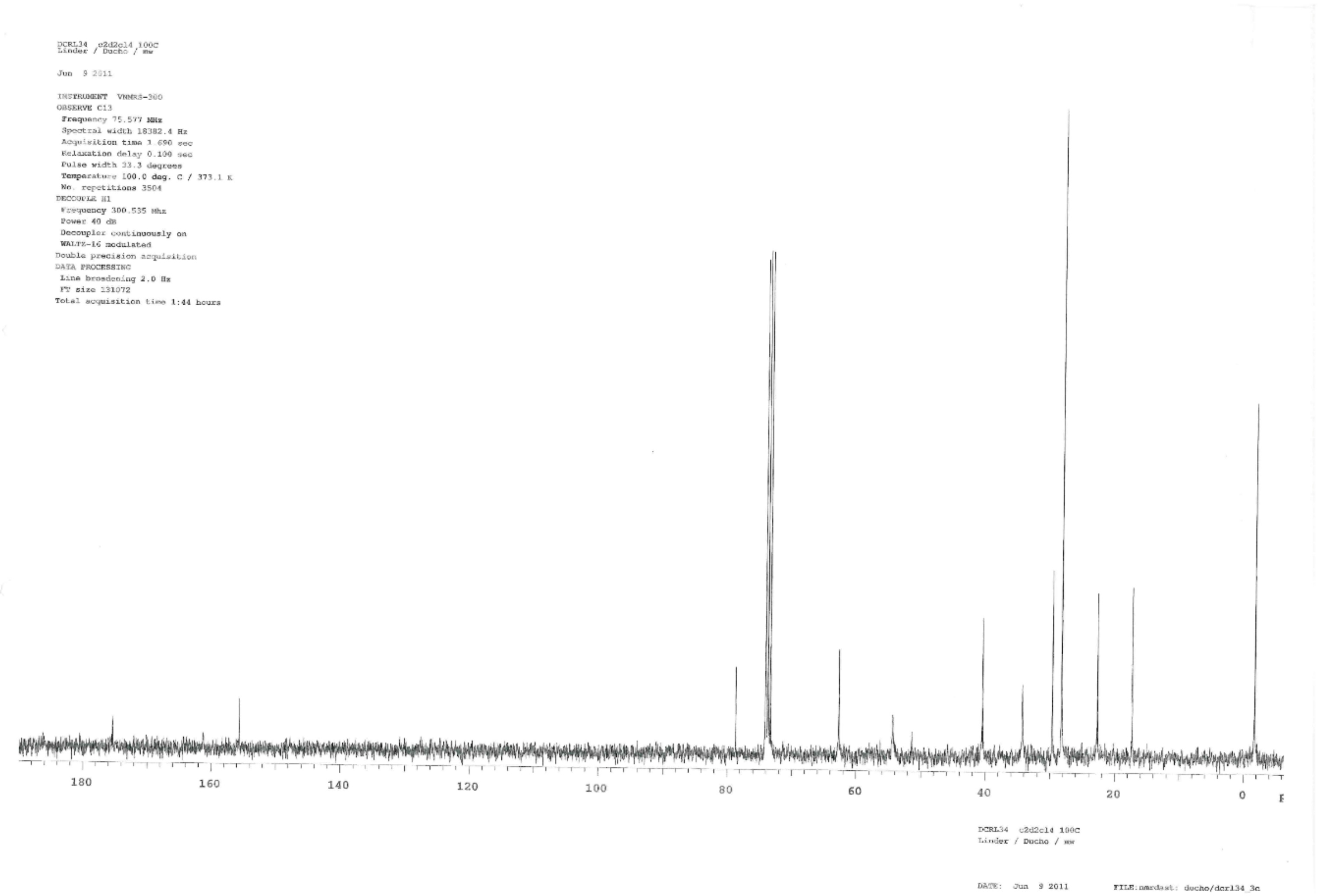

^{13}C NMR spectrum of **24** (75 MHz, CD_2Cl_4, 100 °C)

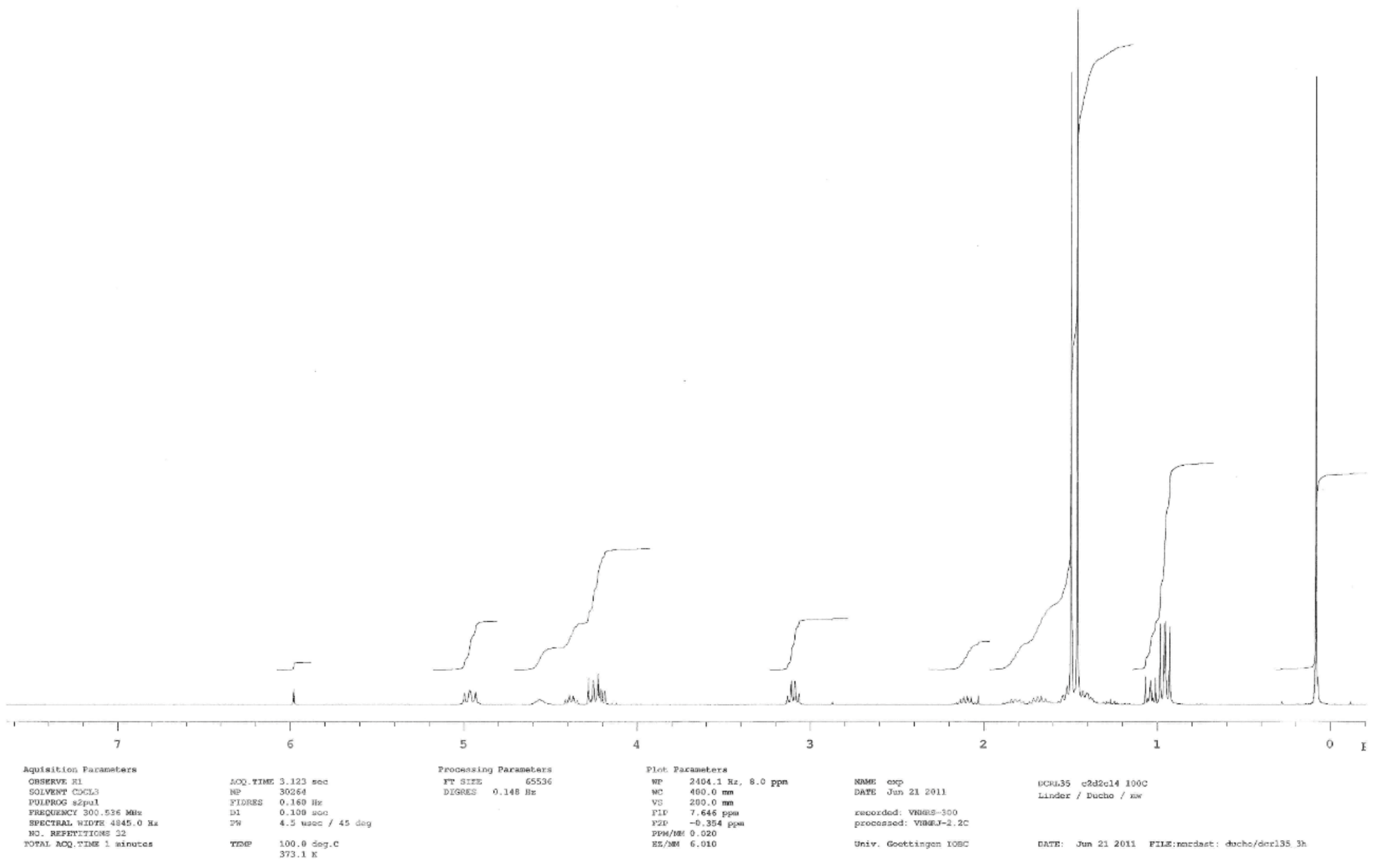

^{1}H NMR spectrum of **26** (300 MHz, CD_2Cl_4, 100 °C)

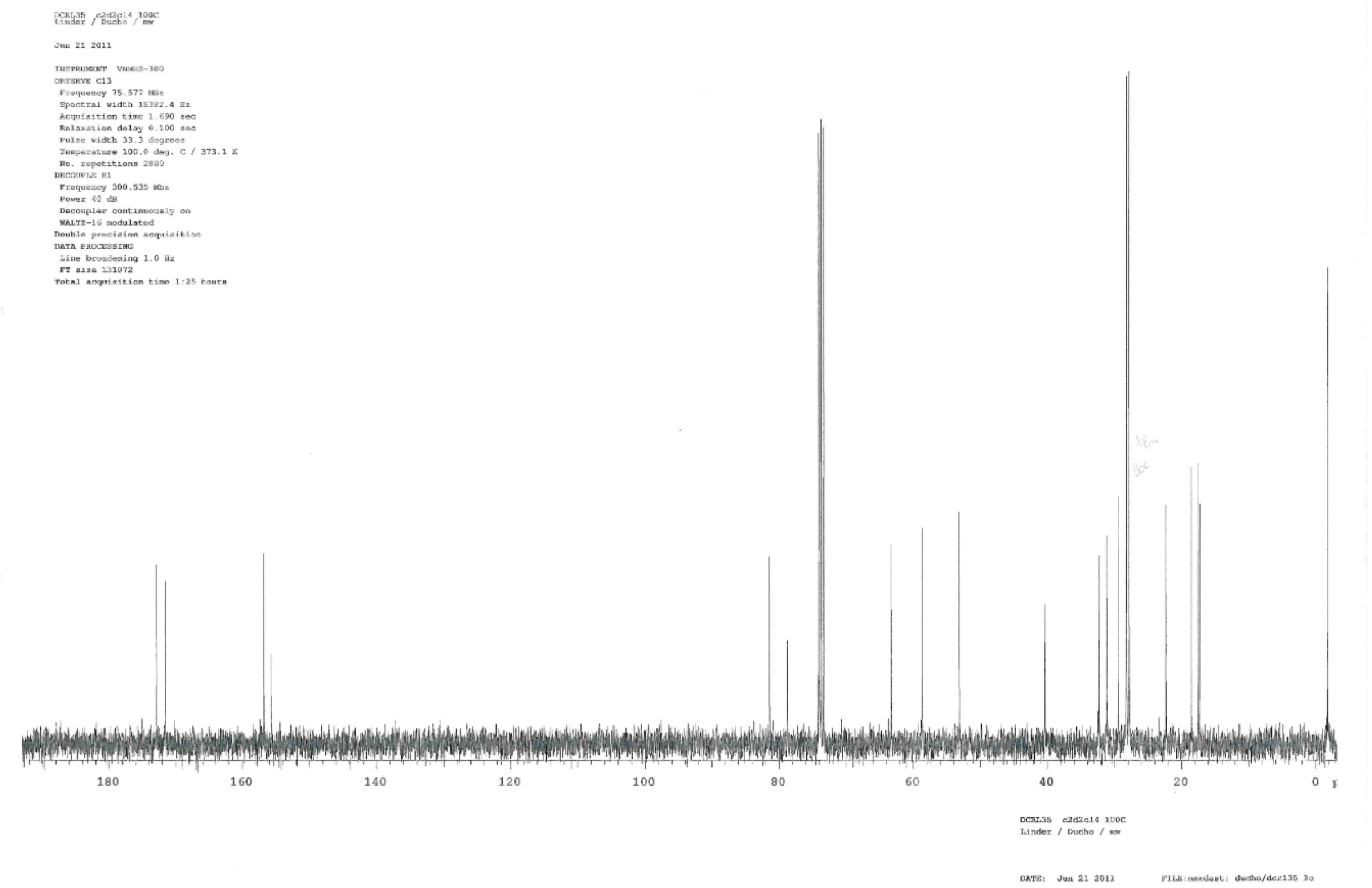

^{13}C NMR spectrum of **26** (75 MHz, CD_2Cl_4, 100 °C)

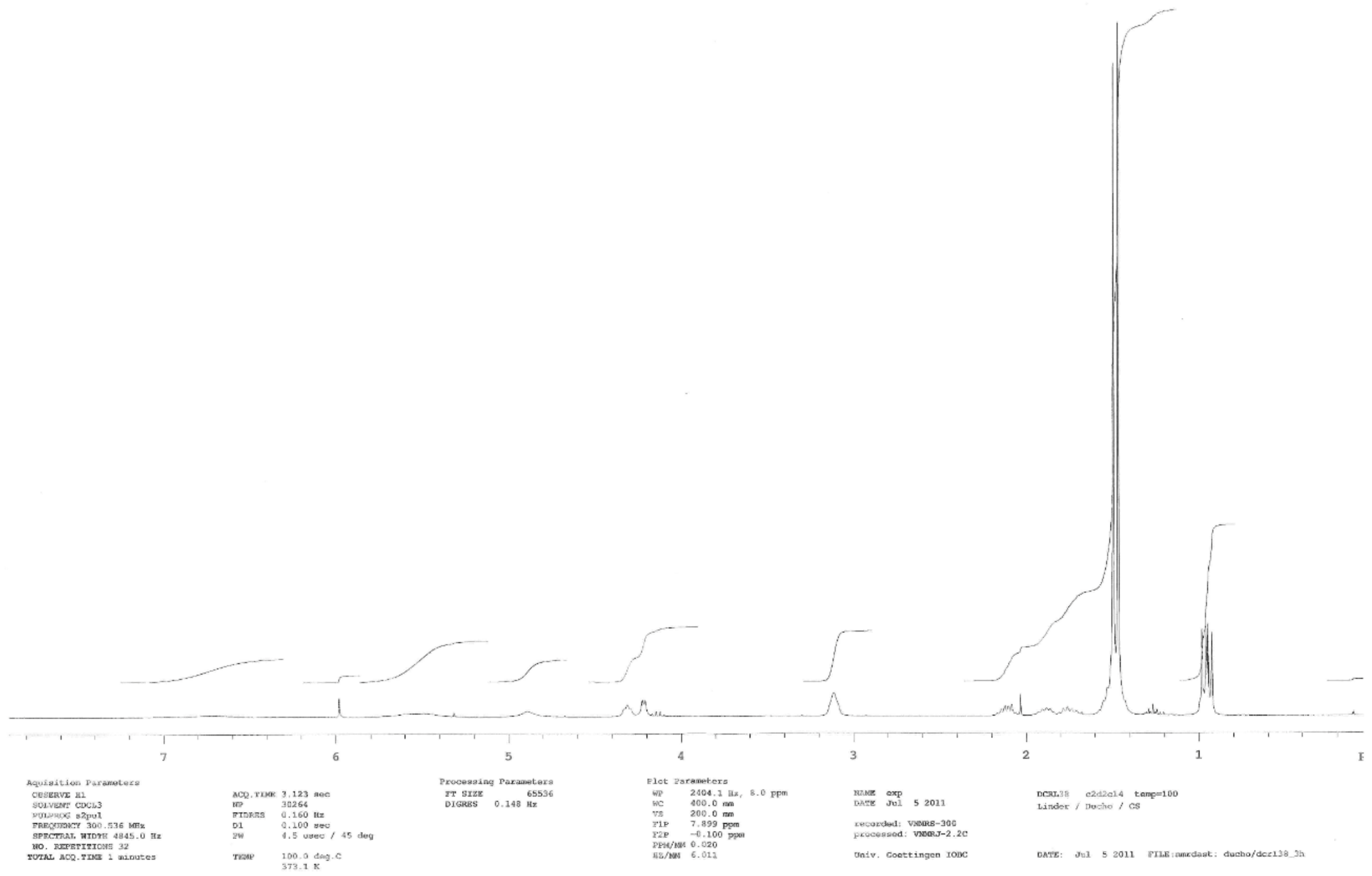

^{1}H NMR spectrum of **27** (300 MHz, CD_2Cl_4, 100 °C)

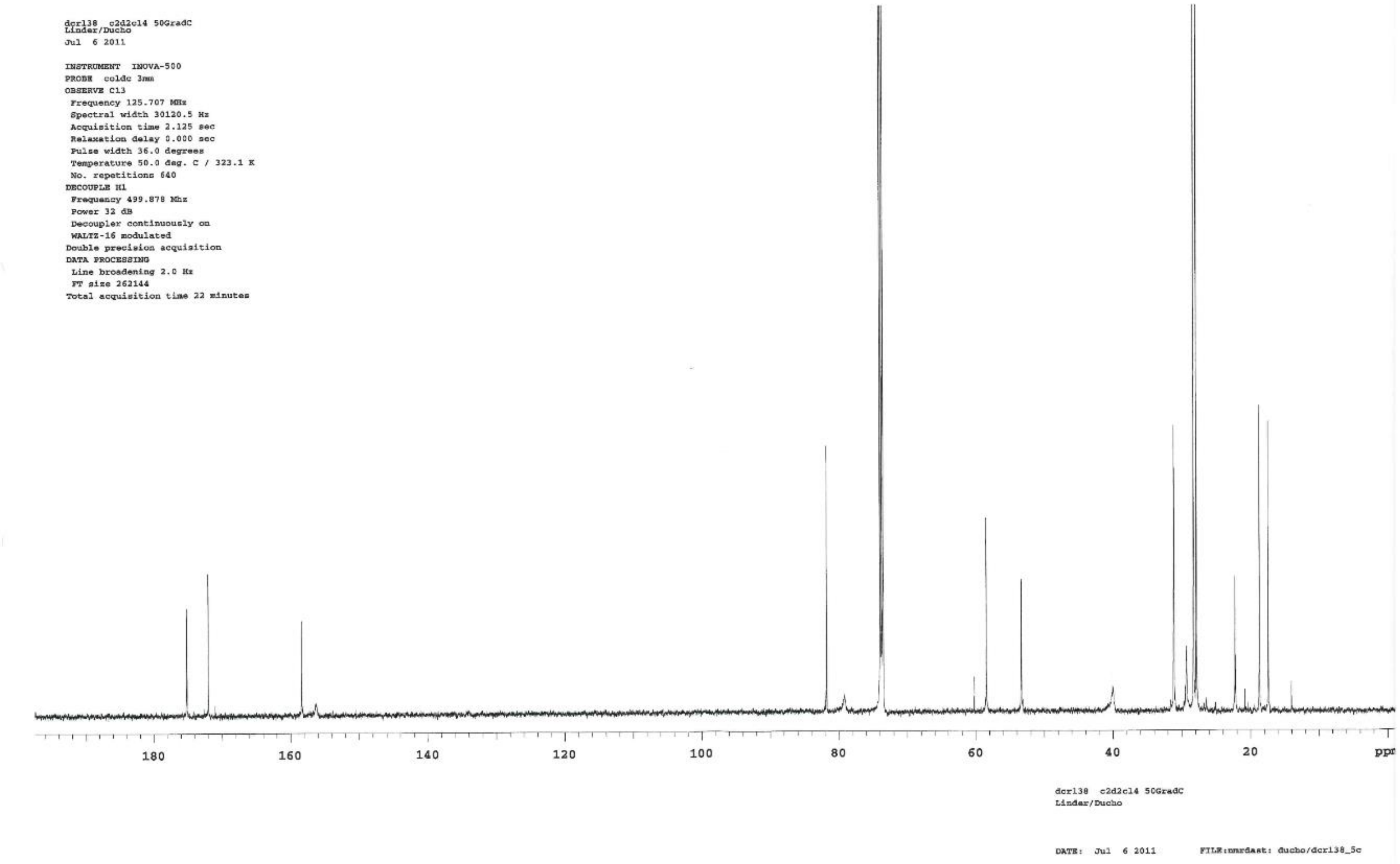

^{13}C NMR spectrum of **27** (75 MHz, CD_2Cl_4, 100 °C)

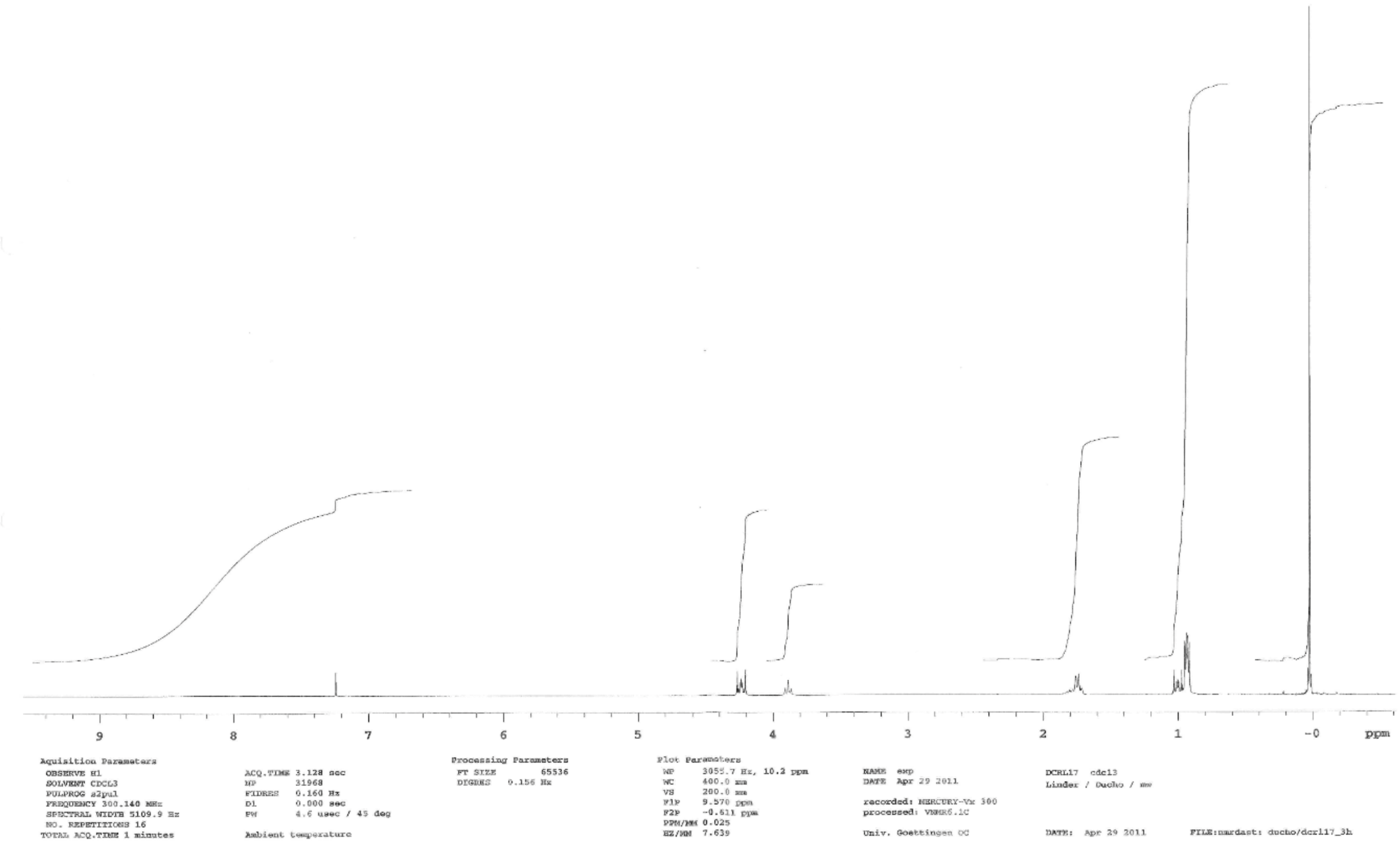

^{1}H NMR spectrum of **28** (300 MHz, $CDCl_3$)

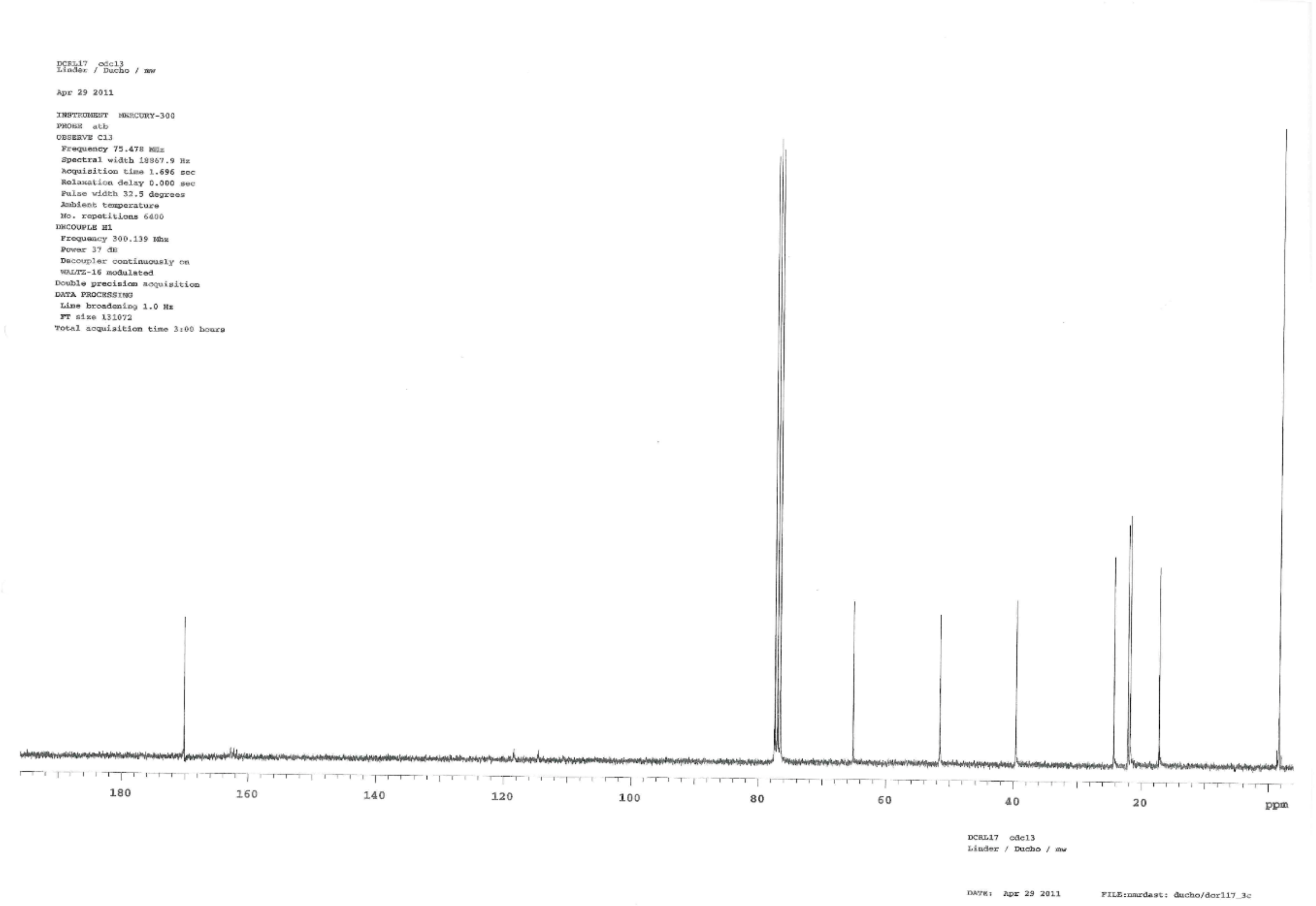

^{13}C NMR spectrum of **28** (75 MHz, $CDCl_3$)

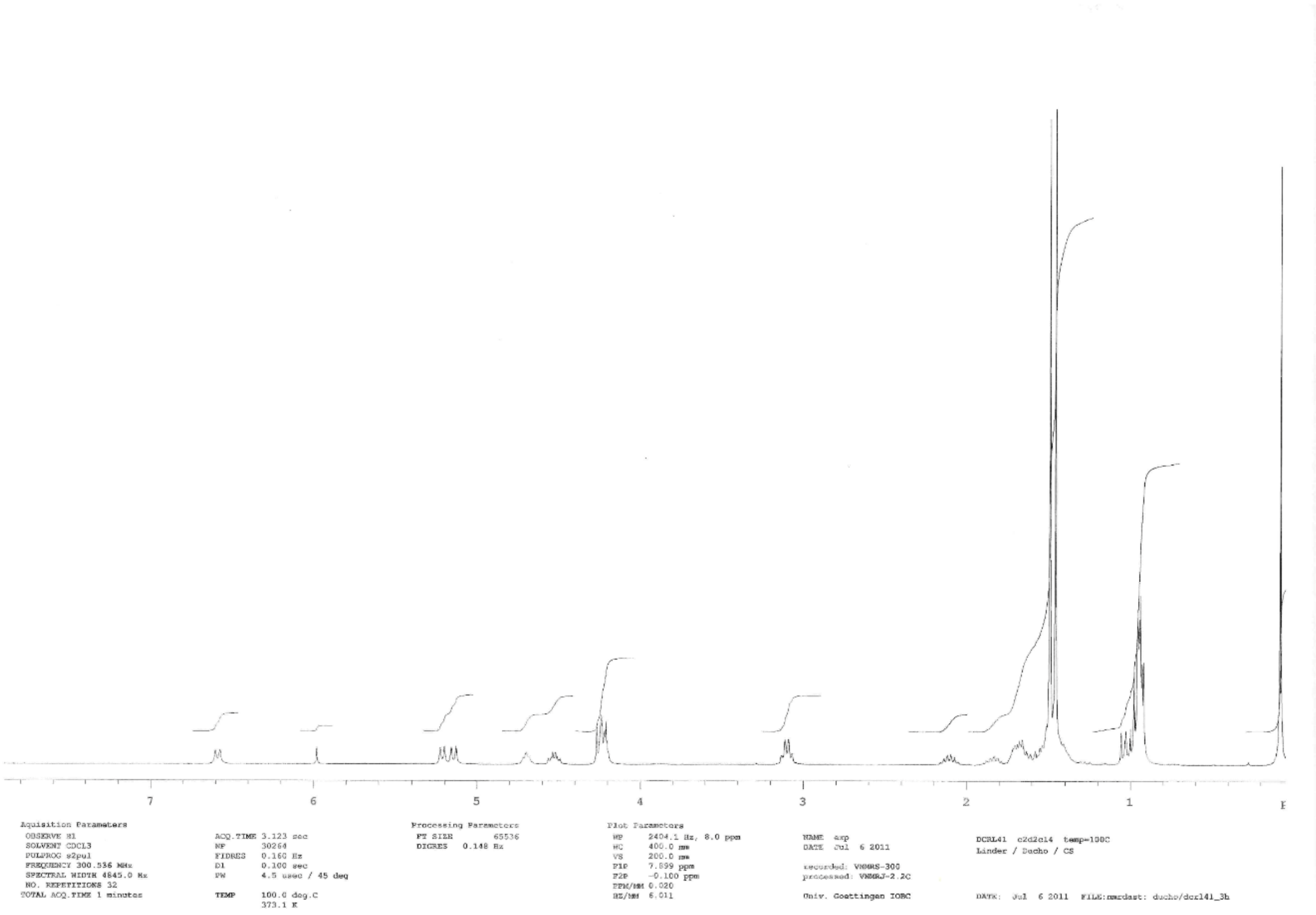

^{1}H NMR spectrum of **29** (300 MHz, CD_2Cl_4, 100 °C)

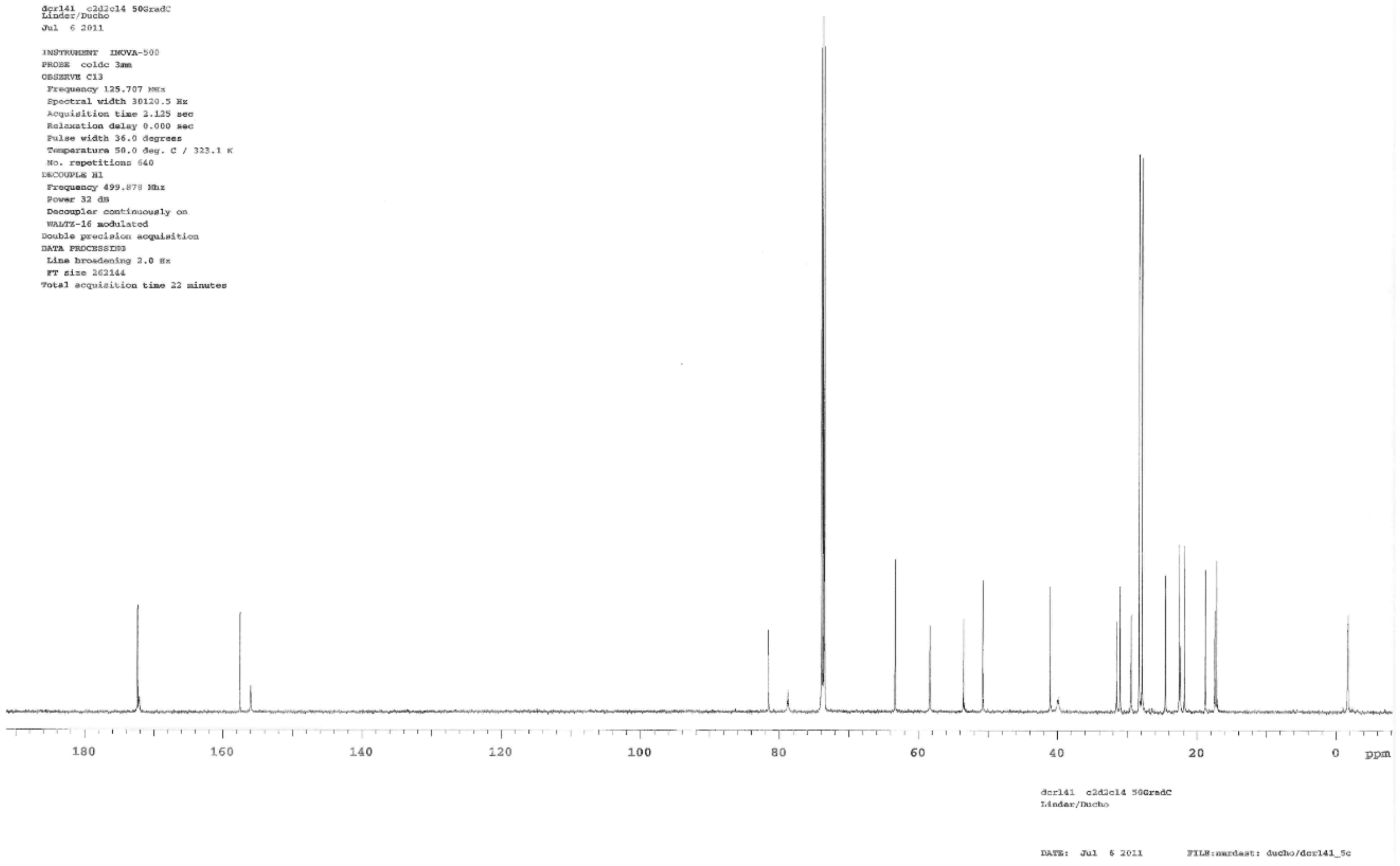

^{13}C NMR spectrum of **29** (75 MHz, CD_2Cl_4, 100 °C)

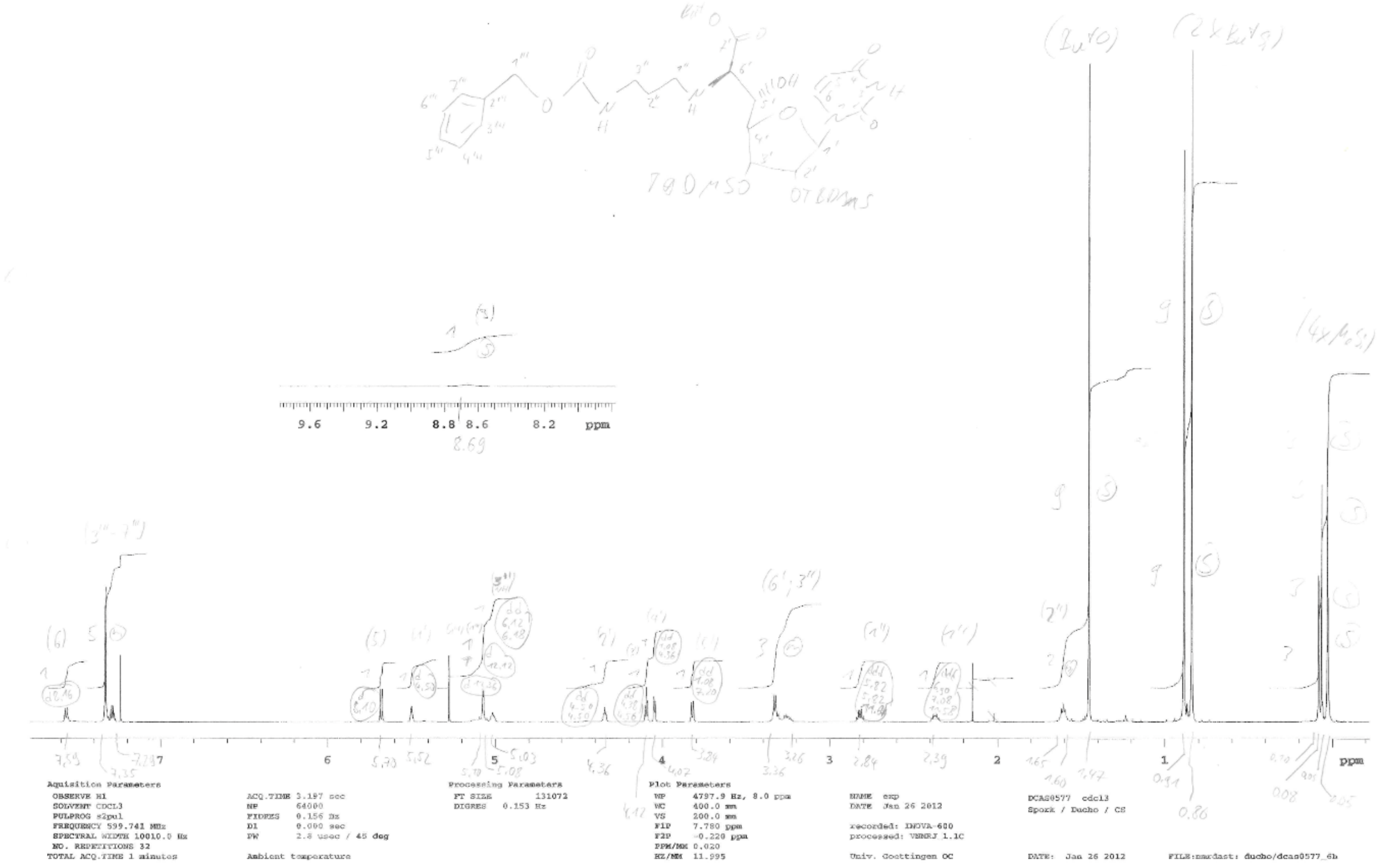

^{1}H NMR spectrum of **33** (600 MHz, $CDCl_3$)

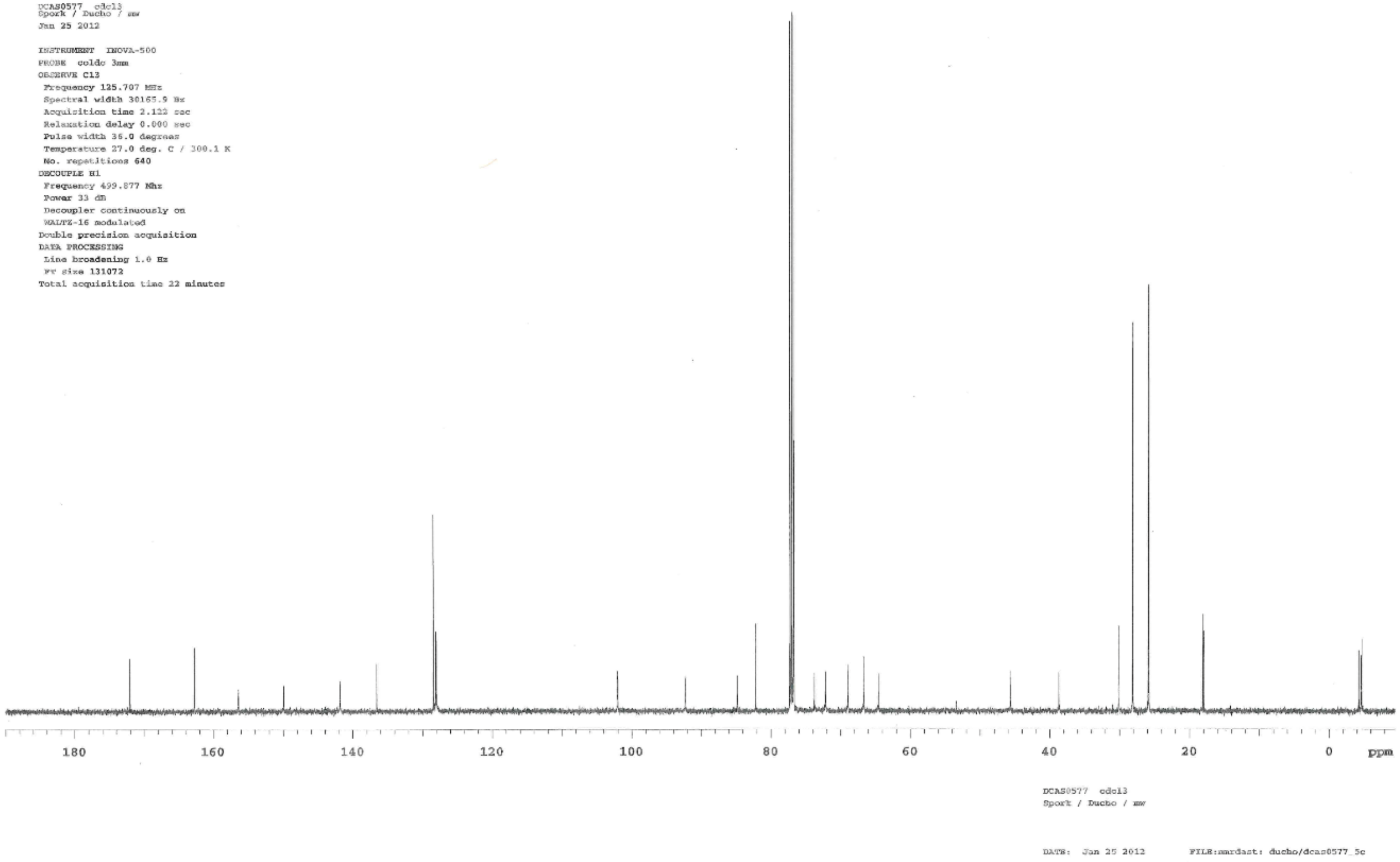

^{13}C NMR of **33** (126 MHz, $CDCl_3$)

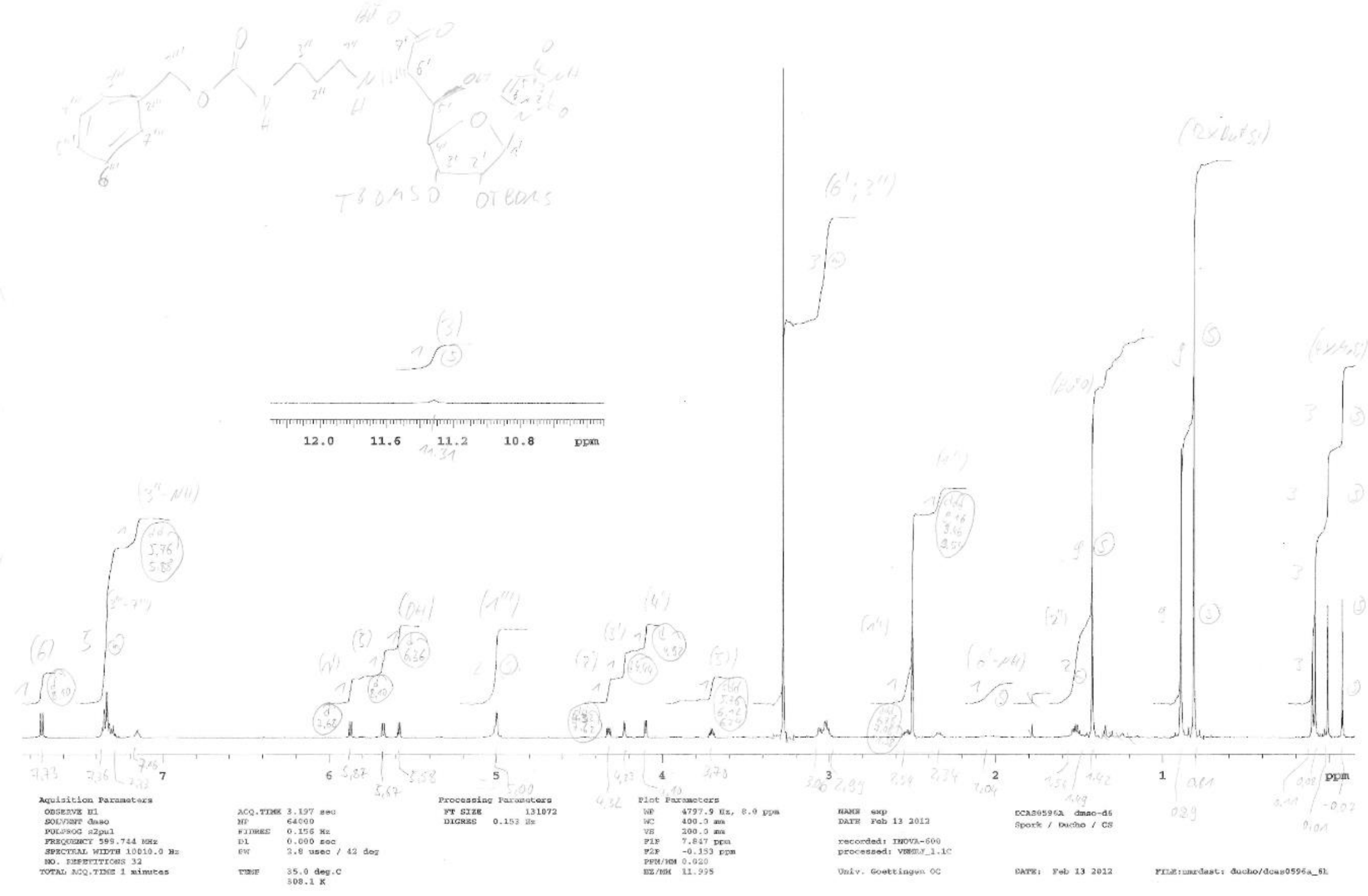

^{1}H NMR spectrum of **35** (600 MHz, DMSO-d_6, 35 °C)

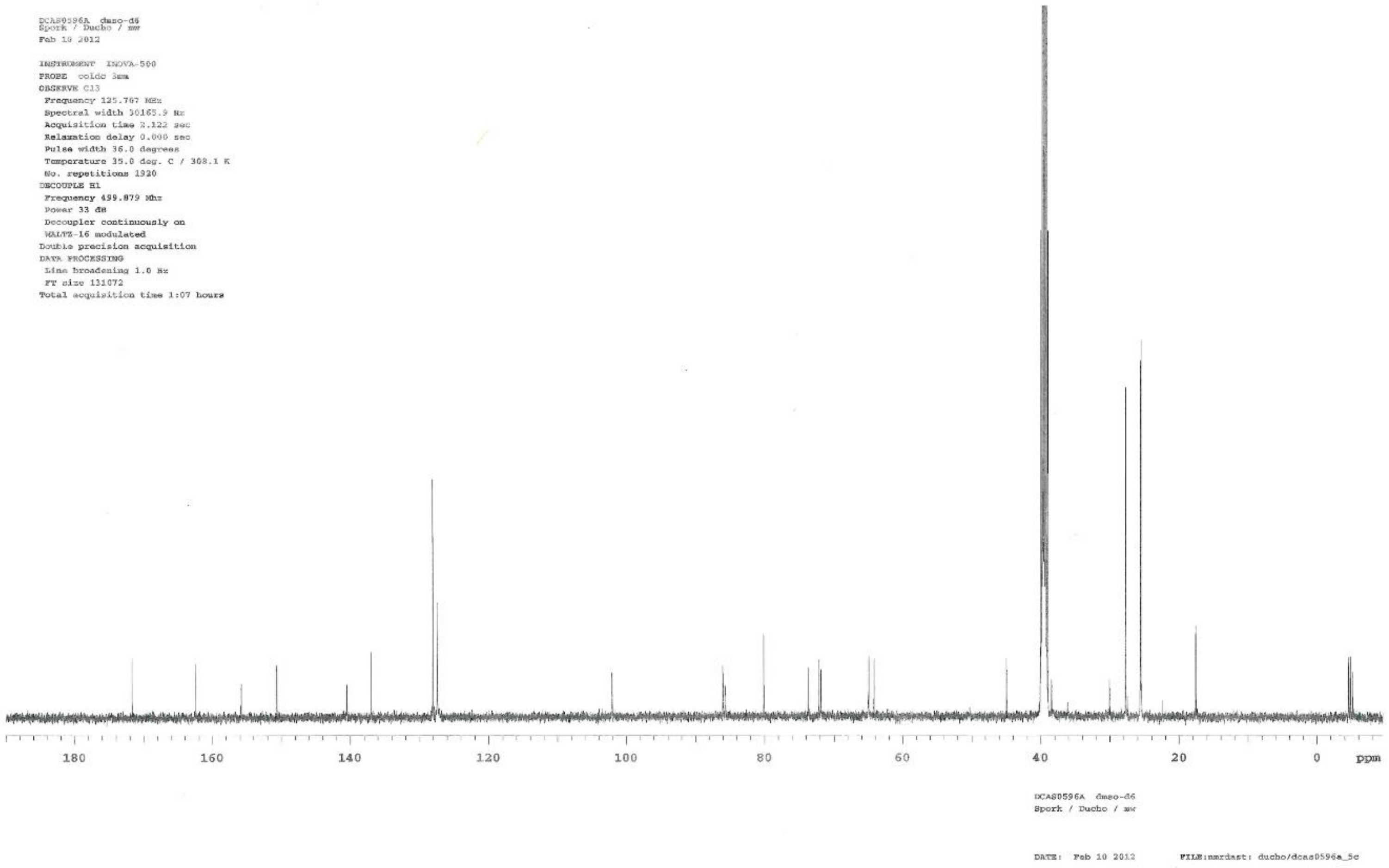

^{13}C NMR spectrum of **35** (126 MHz, DMSO-d_6, 35 °C)

References

1. Marfey, P. Determination of D-amino acids. II. Use of a bifunctional reagent, 1,5-difluoro-2,4-dinitrobenzene. *Carlsberg Res. Commun.* **1984**, *49*, 591, doi:10.1007/BF02908688.
2. Tanino, T.; Ichikawa, S.; Shiro, M.; Matsuda, A. Total Synthesis of (−)-Muraymycin D2 and Its Epimer. *J. Org. Chem.* **2010**, *75*, 1366-1377, doi:10.1021/jo9027193.
3. Bhushan, R.; Brückner, H. Marfey's reagent for chiral amino acid analysis: A review. *Amino Acids* **2004**, *27*, 231-247, doi:10.1007/s00726-004-0118-0.
